# Generation of allogeneic CAR-NKT cells from hematopoietic stem and progenitor cells using a clinically guided culture method

**DOI:** 10.1038/s41587-024-02226-y

**Published:** 2024-05-14

**Authors:** Yan-Ruide Li, Yang Zhou, Jiaji Yu, Yu Jeong Kim, Miao Li, Derek Lee, Kuangyi Zhou, Yuning Chen, Yichen Zhu, Yu-Chen Wang, Zhe Li, Yanqi Yu, Zachary Spencer Dunn, Wenbin Guo, Xinjian Cen, Tiffany Husman, Aarushi Bajpai, Adam Kramer, Matthew Wilson, Ying Fang, Jie Huang, Shuo Li, Yonggang Zhou, Yuchong Zhang, Zoe Hahn, Enbo Zhu, Feiyang Ma, Calvin Pan, Aldons J. Lusis, Jin J. Zhou, Christopher S. Seet, Donald B. Kohn, Pin Wang, Xianghong Jasmine Zhou, Matteo Pellegrini, Benjamin R. Puliafito, Sarah M. Larson, Lili Yang

**Affiliations:** 1 Department of Microbiology, Immunology and Molecular Genetics, University of California, Los Angeles, Los Angeles, CA USA; 2 Department of Medicine, Division of Cardiology, University of California, Los Angeles, Los Angeles, CA USA; 3 Mork Family Department of Chemical Engineering and Materials Science, University of Southern California, Los Angeles, CA USA; 4 Bioinformatics Interdepartmental Program, University of California, Los Angeles, Los Angeles, CA USA; 5 Department of Pathology and Laboratory Medicine, University of California, Los Angeles, Los Angeles, CA USA; 6 Department of Cell and Developmental Biology, Feinberg School of Medicine, Northwestern University, Chicago, IL USA; 7 Human Genetics, University of California, Los Angeles, Los Angeles, CA USA; 8 Department of Biostatistics, Fielding School of Public Health, University of California, Los Angeles, Los Angeles, CA USA; 9 Eli and Edythe Broad Centre of Regenerative Medicine and Stem Cell Research, University of California, Los Angeles, Los Angeles, CA USA; 10 Jonsson Comprehensive Cancer Centre, University of California, Los Angeles, Los Angeles, CA USA; 11 Department of Medicine, Division of Hematology/Oncology, University of California, Los Angeles, Los Angeles, CA USA; 12 Molecular Biology Institute, University of California, Los Angeles, Los Angeles, CA USA; 13 Department of Pediatrics, Division of Hematology/Oncology, University of California, Los Angeles, Los Angeles, CA USA; 14 Institute for Quantitative and Computational Biosciences—The Collaboratory, University of California, Los Angeles, Los Angeles, CA USA; 15 Department of Molecular, Cell, and Developmental Biology, University of California, Los Angeles, Los Angeles, CA USA; 16 Department of Hematology and Oncology, University of California, Los Angeles, Los Angeles, CA USA; 17 Department of Internal Medicine, University of California, Los Angeles, Los Angeles, CA USA

**Keywords:** Stem-cell biotechnology, Cancer immunotherapy

## Abstract

Cancer immunotherapy with autologous chimeric antigen receptor (CAR) T cells faces challenges in manufacturing and patient selection that could be avoided by using ‘off-the-shelf’ products, such as allogeneic CAR natural killer T (^Allo^CAR-NKT) cells. Previously, we reported a system for differentiating human hematopoietic stem and progenitor cells into ^Allo^CAR-NKT cells, but the use of three-dimensional culture and xenogeneic feeders precluded its clinical application. Here we describe a clinically guided method to differentiate and expand IL-15-enhanced ^Allo^CAR-NKT cells with high yield and purity. We generated ^Allo^CAR-NKT cells targeting seven cancers and, in a multiple myeloma model, demonstrated their antitumor efficacy, expansion and persistence. The cells also selectively depleted immunosuppressive cells in the tumor microenviroment and antagonized tumor immune evasion via triple targeting of CAR, TCR and NK receptors. They exhibited a stable hypoimmunogenic phenotype associated with epigenetic and signaling regulation and did not induce detectable graft versus host disease or cytokine release syndrome. These properties of ^Allo^CAR-NKT cells support their potential for clinical translation.

## Abstract (web summary)

Off-the-shelf CAR-NKT cells for cancer immunotherapy are advanced toward clinical translation.

## Main

CAR-T cell therapies^1^ have received Food and Drug Administration approval for the treatment of B cell malignancies and multiple myeloma (MM)^2^. However, autologous CAR-T cell products are associated with high costs, prolonged manufacturing time and limited accessibility to patients^3^. In particular, patients with progressive disease or who have received previous treatments may not have adequate or functional T cells for CAR-T cell production. To develop off-the-shelf cellular products, two approaches are being explored. One uses conventional αβ T cells with abrogation of endogenous T cell receptor (TCR) expression to minimize the risk of graft-versus-host disease (GvHD)^4^. The other uses cell types that inherently pose low GvHD risk, such as macrophages, NK cells and invariant natural killer T (iNKT or NKT) cells^5–7^.

NKT cells, characterized by an invariant TCR α chain (TRAV10-TRAJ18 in human and TRAV11-TRAJ18 in mice) and coexpression of NK markers, are a unique type of αβ T cell that is relatively rare in the human bloodstream, accounting for only 0.001–1% of circulating T cells^8–11^. Owing to their TCR recognition of the non-polymorphic major histocompatibility complex-like molecule CD1d (ref. ^12^), these cells are not expected to induce GvHD^13^. NKT cells have demonstrated promising characteristics for the development of off-the-shelf cell therapy. They possess potent tumor-killing activity and can infiltrate tumors, and they bridge innate and adaptive immune responses^14^. In clinical studies, autologous CAR-engineered NKT (CAR-NKT) cells have shown efficacy in treating relapsed or resistant neuroblastoma without causing noticeable toxicity or cytokine release syndromes (CRSs)^6,15^. However, the development of NKT cell therapies is limited by the scarcity of NKT cells in human blood and the difficulty of expanding them from peripheral blood mononuclear cells (PBMCs) while excluding conventional T cells, which could trigger GvHD^13^. Thus, identifying clinically adaptable methods to allogeneic NKT cells from sources other than PBMCs is of interest.

Hematopoietic stem and progenitor cells (HSPCs) have been genetically engineered and differentiated into various types of immune cell^16,17^, including NKT cells^18,19^. In a previous study, we differentiated engineered cord-blood HSPCs into NKT cells using an artificial thymic organoid culture method^18^. However, the use of three-dimensional (3D) culture and xenogeneic feeder cells precluded scale-up and clinical manufacturing^18^. To develop a clinically relevant system, here we describe a method for differentiating and expanding allogeneic CAR-NKT (^Allo^CAR-NKT) cells free of 3D culture and xenogeneic feeder cells. We generated ^Allo^CAR-NKT cells targeting two blood cancer markers (BCMA and CD19) and three solid tumor markers (GD2, GPC3 and EGFRvIII). We also engineered ^Allo^CAR-NKT cells with human IL-15, which is well recognized for its role in enhancing effector function, promoting memory formation and mitigating exhaustion in T, NK and NKT cells^20–22^. Finally, we conducted comprehensive preclinical studies of ^Allo^CAR-NKT cells to evaluate their manufacturing, pharmacology, efficacy, mechanism of action, pharmacokinetics and/or pharmacodynamics (PK–PD), safety and immunogenicity.

## Results

### Ex vivo generation of ^Allo^CAR-NKT cells

**Fig. 1:**
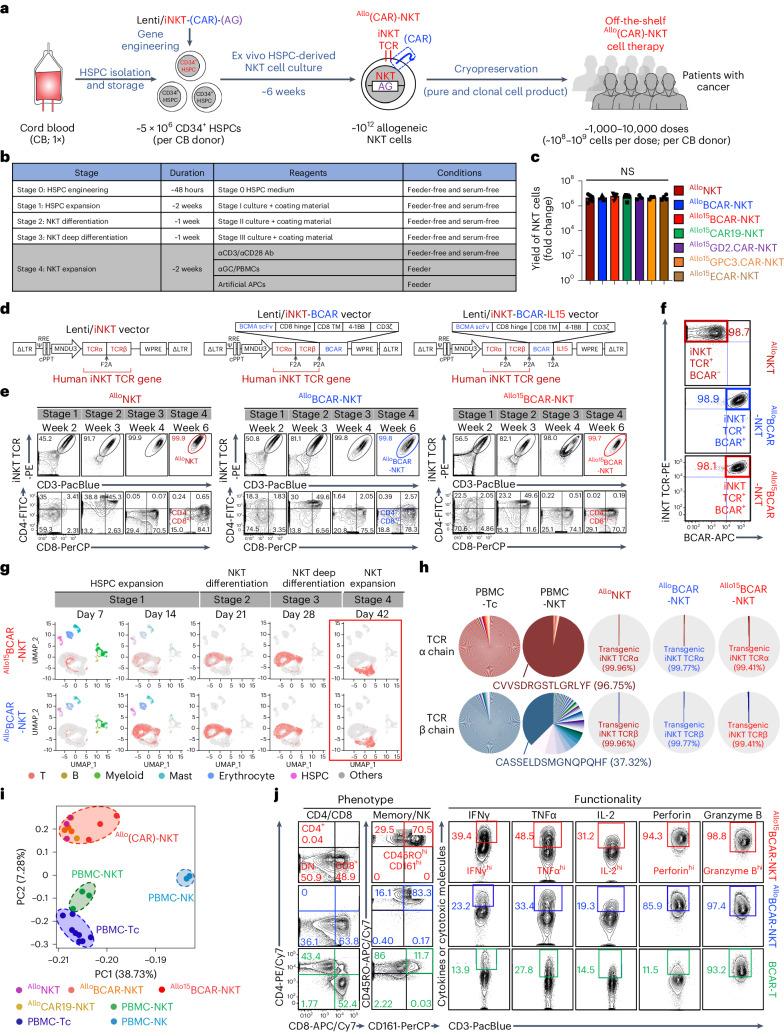
^Allo^NKT and ^Allo^CAR-NKT cells are produced at high yield, purity and robustness. **a**, Schematics showing the generation of ^Allo^(CAR)-NKT cells. AG, additional gene; CB, cord blood. **b**, Table showing the stages of ^Allo^(CAR)-NKT cell culture. **c**, Yield of ^Allo^(CAR)-NKT cells (^Allo^NKT and ^Allo^BCAR-NKT, *n* = 6; ^Allo15^BCAR-NKT and ^Allo15^CAR19-NKT, *n* = 9; ^Allo15^GD2.CAR-NKT, *n* = 3; ^Allo15^GPC3.CAR-NKT and ^Allo15^ECAR-NKT, *n* = 4; *n* indicates different donors). **d**, Schematics showing the design of indicated lentivectors. ΔLTR, self-inactivating long-term repeats; MNDU3, internal promoter derived from the MND retroviral LTR U3 region; φ, packaging signal with the splicing donor and splicing acceptor sites; RRE, reverse-responsive element; cPPT, central polypurine tract; WPRE, woodchuck responsive element. **e**, FACS monitoring of the generation of ^Allo^(CAR)-NKT cells. ^Allo^BCAR-NKT, ^Allo^NKT cells armed with BCAR; ^Allo15^BCAR-NKT, ^Allo^NKT cells armed with BCAR and IL-15. iNKT TCR was stained using a 6B11 monoclonal antibody. **f**, FACS detection of the iNKT TCR and BCAR on ^Allo^(CAR)-NKT cells. BCAR was stained using an antimouse IgG F(ab’)2 antibody. **g**, UMAP visualization of ^Allo/15^BCAR-NKT cell development. scRNA-seq data are presented. Each dot represents a single cell and is colored according to its cell cluster assignment. **h**, Single-cell TCR sequencing analyses of ^Allo^(CAR)-NKT cells. Healthy donor PBMC-derived conventional αβ T (PBMC-Tc), NKT (PBMC-NKT) and NK (PBMC-NK) cells were included as controls. **i**, Bulk RNA-seq analyses of ^Allo^(CAR)-NKT cells. ^Allo^NKT (*n* = 3), ^Allo^BCAR-NKT (*n* = 3), ^Allo15^BCAR-NKT (*n* = 3) and ^Allo^CAR19-NKT (*n* = 2) cells were analyzed. PBMC-Tc (*n* = 8), PBMC-NKT (*n* = 3) and PBMC-NK (*n* = 2) cells were included as controls. *n* indicates different donors. ^Allo^CAR19-NKT, ^Allo^NKT cells armed with CD19-targeting CAR. **j**, FACS detection of surface markers, intracellular cytokines and cytotoxic molecules in ^Allo/15^BCAR-NKT cells. PBMC-derived conventional T cells engineered with the same BCMA-targeting CAR (denoted as BCAR-T cells) were included as a control. Representative of one (**g**–**i**) and more than ten (**a**–**f** and **j**) experiments. Data are presented as the mean ± s.e.m. and were analyzed by one-way ANOVA (**c**). NS, not significant. Source data

Cryopreserved human cord-blood-derived CD34^+^ HSPCs were obtained from commercial vendors and used to generate NKT cells in a five-stage, 6-week process (Fig. 1a–c).

At stage 0, freeze–thawed CD34^+^ HSPCs were transduced with a lentivector codelivering a human iNKT TCR, a designated CAR, as well as additional genes, and cultured over 48 hours in a conventional X-VIVO 15-based HSPC medium (Fig. 1a,b,d and Supplementary Fig. 1a,b)^18,23^. All lentivectors tested showed robust HSPC transduction (Supplementary Fig. 2a). The iNKT TCR genes were cloned from a healthy donor and validated for its effective recognition of CD1d and lipid antigens^18,23^. CARs were studied that target various tumor antigens such as the B cell maturation antigen (BCMA), CD19, disialoganglioside GD2 (GD2), glypican-3 (GPC3) and epidermal growth factor receptor variant III (EGFRvIII), and contain different costimulatory domains including CD28 and 4-1BB (Fig. 1d and Supplementary Fig. 1a)^24–28^. An example of an additional gene (AG) is *IL-15*, which has been shown to augment the in vivo persistence and antitumor reactivity of NKT cells^6,26,29,30^.

Gene-engineered HSPCs were then cultured over roughly 6 weeks to generate a designated ^Allo^CAR-NKT cell product: stage 1 HSPC expansion (2 weeks), stage 2 NKT differentiation (1 week), stage 3 NKT deep differentiation (1 week) and stage 4 NKT expansion (2 weeks) (Fig.1a,b,e and Supplementary Fig. 1c). We implemented the entire culture in a feeder-free and serum-free manner for compatibility with clinical translation^31^. We also demonstrated two alternative strategies that use human feeder cells, either α-galactosylceramide (αGC)-loaded healthy donor PBMCs or K562-based artificial antigen-presenting cells (aAPCs), during stage 4 NKT expansion, as these human feeder cells have been validated for clinical development (Fig. 1b and Supplementary Fig. 2b)^15,31^.

The differentiation of ^Allo^CAR-NKT cells followed a typical T cell lineage commitment and NKT cell developmental path, from CD4^−^CD8^−^ double-negative to CD4^+^CD8^+^ double-positive and eventually to CD8^+^ single-positive or double-negative stage (Fig. 1e,g and Supplementary Fig. 1c)^32^. The final ^Allo^CAR-NKT cells lacked a CD4^+^ single-positive population, which is typically present in endogenous human NKT cells (Fig. 1e and Supplementary Fig. 1c)^32^. Further examination of CD8^+ Allo^CAR-NKT cells showed a predominant expression of the CD8α/α form (~80%) over the CD8α/β form (~20%), resembling the CD8α/α versus CD8α/β expression pattern in the endogenous NKT cells (Supplementary Fig. 3)^32^. In general, CD8α/α and double-negative human NKT cells are considered of similar functionality; these cells are proinflammatory and exhibit heightened cytotoxicity, making them desirable for cancer immunotherapy^32^. Incorporation of a CAR gene or CAR and IL-15 genes did not interfere with the differentiation of allogeneic NKT cells and yielded allogeneic NKT cells that universally coexpressed the CAR (and IL-15) alongside the iNKT TCR (Fig. 1f and Supplementary Fig. 1c).

The yield and purity of ^Allo^CAR-NKT cells were high. A more than 10^6^-fold expansion was achieved from the input CD34^+^ HSPCs to the output mature ^Allo^CAR-NKT cells (Fig. 1c). In our cultures, we routinely generated roughly 10^10^ mature ^Allo^CAR-NKT cells from 10^4^ input HSPCs (Supplementary Fig. 2c–f). Flow cytometry and single-cell TCR sequencing analyses revealed uniform expression of the transgenic iNKT TCRs in ^Allo^CAR-NKT cells (Fig. 1e,h and Supplementary Fig. 1c). Randomly recombined endogenous αβ TCRs were undetected, likely attributed to allelic exclusion induced by the overexpression of the transgenic TCRs (Fig. 1e,h)^33^.

The ^Allo^CAR-NKT cell production process was robust, spanning 15 cord-blood donors and various cargo genes (Fig. 1c–e and Supplementary Fig. 1). These cargo genes included CARs with different designs, as well as additional genes such as *IL-15* (Fig. 1d and Supplementary Fig. 1). The variability in cord-blood donors and cargo genes had no discernible impact on the high yield and purity of the final product cells (Fig. 1c).

In this study, we performed comprehensive preclinical characterization of ^Allo^CAR-NKT cells, with a primary focus on MM as the disease indication and its corresponding BCMA-targeting ^Allo^CAR-NKT cells, particularly those incorporating IL-15 enhancement (^Allo/15^BCAR-NKT cells).

### Phenotype and functionality of ^Allo^CAR-NKT cells

We analyzed the phenotype and functionality of ^Allo/15^BCAR-NKT cells in comparison with endogenous human PBMC-derived conventional αβ T, NK and NKT cells (denoted PBMC-Tc, PBMC-NK and PBMC-NKT cells, respectively) (Supplementary Fig. 4a). PBMC-Tc cells engineered with the same BCMA-targeting CAR (BCAR) (conventional BCAR-T cells) were also included as a benchmark control (Supplementary Fig. 4b,c)^24^.

Gene profiling using bulk RNA sequencing (RNA-seq) revealed that ^Allo/15^BCAR-NKT cells closely resembled PBMC-NKT cells, ranked second to PBMC-Tc cells and were largely different from PBMC-NK cells (Fig. 1i). To validate iNKT TCR function, we stimulated ^Allo/15^BCAR-NKT cells with an agonist glycolipid antigen αGC (Supplementary Fig. 5). ^Allo/15^BCAR-NKT cells proliferated robustly (Supplementary Fig. 5a–c) and secreted high levels of Th1 cytokines (that is, IFNγ and TNFα) while low levels of Th2/Th17 cytokines (that is, IL-4 and IL-17a) (Supplementary Fig. 5d), indicating a Th1-prone functionality of ^Allo/15^BCAR-NKT cells, which agrees with their CD8 single-positive and/or double-negative phenotype regardless of IL-15 engineering (Fig. 1e and Supplementary Fig. 5e)^32^.

Flow cytometry analysis of ^Allo/15^BCAR-NKT cells revealed a typical NKT cell phenotype (Fig. 1j). Compared with conventional BCAR-T cells, ^Allo/15^BCAR-NKT cells expressed high levels of NK receptors (NKRs) (for example, CD161) and central memory markers (for example, CD62L, CD134 and LEF1), and produced exceedingly high levels of effector cytokines (for example, IFNγ, IL-2 and TNFα) and cytotoxic molecules (for example, Granzyme B and Perforin), agreeing with their CD8 single-positive and/or double-negative phenotype (Fig. 1j and Supplementary Fig. 5f)^34,35^.

### In vitro antitumor efficacy and mechanism of action of ^Allo^CAR-NKT cells

**Fig. 2:**
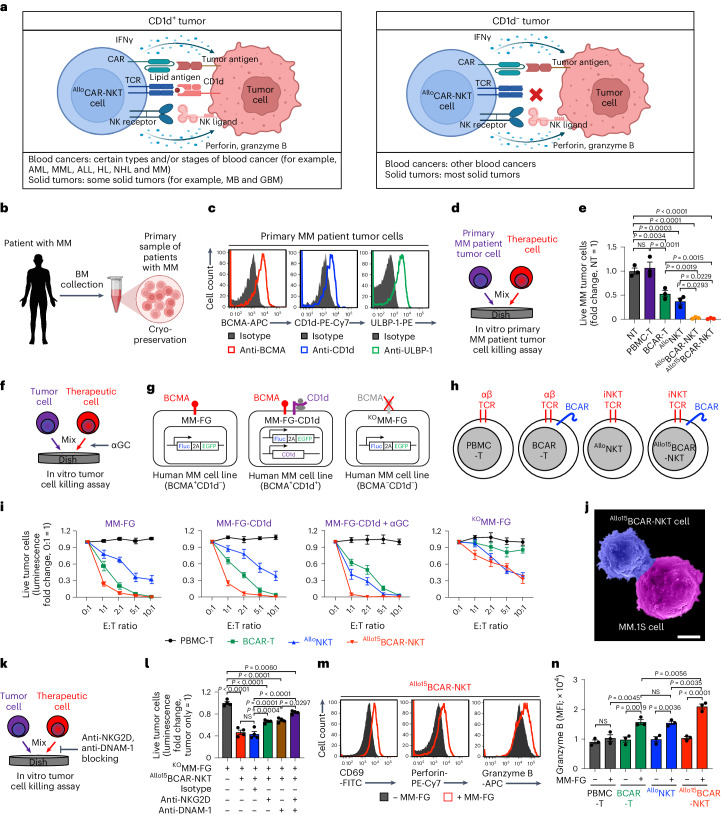
^Allo^CAR-NKT cells directly kill tumor cells and use multiple targeting mechanisms. **a**, Diagram showing the mechanisms used by ^Allo^CAR-NKT cells to kill CD1d^+^ or CD1d^−^ tumor cells. AML, acute myeloid leukemia; JMML, juvenile myelomonocytic leukemia; ALL, acute lymphoblastic leukemia; HL, Hodgkin lymphoma; NHL, non-Hodgkin lymphoma; MB, medulloblastoma; GBM, glioblastoma. **b**–**e**. Studying the antitumor efficacy of ^Allo/15^BCAR-NKT cells against primary samples from patients with MM. **b**, Diagram showing the collection of BM samples from patients with MM. **c**, FACS detection of CAR target (BCMA), iNKT TCR target (CD1d) and NKR target (ULBP-1) on primary patient-derived tumor cells. **d**, Experimental design to study the primary MM tumor cell killing by therapeutic cells. **e**, Tumor cell killing data at 24 h (*n* = 3). **f**–**j**. Studying the antitumor efficacy of ^Allo15^BCAR-NKT cells against human MM.1S cell lines. **f**, Experimental design. **g**, Schematics showing the indicated human MM.1S cell lines. MM-FG, MM.1S cell line engineered to express the firefly luciferase and enhanced green fluorescence protein (FG) dual reporters; MM-FG-CD1d, MM-FG cell line further engineered to overexpress human CD1d; ^KO^MM-FG, MM-FG cell line further engineered to knockout the BCMA encoding gene. **h**, Schematics showing the indicated therapeutic cells. **i**, Tumor cell killing data at 24 h (*n* = 4). **j**, SEM image showing that an ^Allo15^BCAR-NKT cell (blue) is attacking an MM.1S-FG tumor cell (purple). Scale bar, 5 μm. **k**–**l**, Studying the tumor-killing mechanism of ^Allo15^BCAR-NKT cells mediated by NK activating receptors (that is, NKG2D and DNAM-1). **k**, Experimental design. **l**, Tumor cell killing data at 24 h (E:T ratio 10:1; *n* = 4 from four different cell product donors). **m**,**n**, FACS characterization of ^Allo15^BCAR-NKT cells 24 h after coculturing with MM-FG. **m**, FACS detection of surface CD69 as well as intracellular Perforin and Granzyme B in ^Allo15^BCAR-NKT cells. **n**, Quantification of **m** (*n* = 3 from three different cell product donors). Representative of three experiments. Data are presented as the mean ± s.e.m. and were analyzed by one-way ANOVA (**e**, **l** and **n**). Source data

^Allo^CAR-NKT cells are expected to target tumor cells through multiple surface receptors, involving CAR recognition of CAR antigen, TCR recognition of CD1d and NKR recognition of NK ligands expressed on most tumor cells (Fig. 2a)^18^. ^Allo^CAR-NKT cells could deploy CAR/TCR/NKR triple-targeting mechanisms, suitable for CD1d^+^ tumors, such as certain types of blood cancer (for example, MM, acute myeloid leukemia and myelomonocytic leukemia) and some solid tumors (for example, medulloblastoma and glioblastoma) (Fig. 2a)^18^. For CD1d^−^ tumors, including other blood cancers and most solid tumors, ^Allo^CAR-NKT cells could still deploy CAR/NKR double-targeting mechanisms (Fig. 2a)^18^.

A cohort of primary bone marrow (BM) samples from patients with MM was collected and used to evaluate the tumor cell killing efficacy of ^Allo/15^BCAR-NKT cells (Fig. 2b). Coexpression of BCMA, CD1d and NK ligands was detected on MM cells from all samples (Fig. 2c and Supplementary Fig. 6). In an in vitro tumor cell killing assay, ^Allo/15^BCAR-NKT cells demonstrated superior effectiveness in eliminating primary MM cells compared with conventional BCAR-T cells (Fig. 2d,e). Even without CAR engineering, ^Allo^NKT cells displayed considerable tumor-killing capabilities, albeit slightly lower than that of ^Allo/15^BCAR-NKT cells (Fig. 2d,e), indicating the CAR-independent tumor-killing potential of ^Allo/15^BCAR-NKT cells.

The CAR/TCR/NKR triple-targeting mechanisms of ^Allo15^BCAR-NKT cells were further validated using a series of in vitro tumor cell killing assays (Fig. 2f–n), involving three human MM.1S-derived cell lines as targets (MM-FG-CD1d that was BCMA^+^CD1d^+^, MM-FG that was BCMA^+^CD1d^−^ and ^KO^MM-FG that was BCMA^−^CD1d^−^) and four therapeutic cells (^Allo15^BCAR-NKT cells, and three controls cells: PBMC-T, BCAR-T and ^Allo^NKT) (Fig. 2f–h and Supplementary Fig. 7a–c). BCAR-T cells solely relied on BCAR-BCMA recognition for tumor cell cytotoxicity, as evidenced by selective killing of BCMA^+^ tumor cells (Fig. 2i). In contrast, ^Allo15^BCAR-NKT cells exhibited cytotoxicity against both BCMA^+^ and BCMA^−^ tumor cells, indicating a less pronounced dependence on BCAR-BCMA recognition, albeit with a comparatively reduced killing capacity toward BCMA^−^ tumor cells (Fig. 2i). Meanwhile, ^Allo15^BCAR-NKT cells killed tumor cells more effectively in the presence of CD1d/αGC, indicating a TCR-directed targeting mechanism (Fig. 2i). Moreover, ^Allo15^BCAR-NKT cells could kill BCMA^−^CD1d^−^ tumor cells through NKR (that is, NKG2D and DNAM-1) recognition, confirming an NKR-mediated targeting mechanism (Fig. 2k,l and Supplementary Fig. 7d–f).

We visualized the direct recognition and attack of MM.1S tumor cells by ^Allo15^BCAR-NKT cells by scanning electron microscopy (SEM) (Fig. 2j). Corresponding to their multiple targeting mechanisms (Fig. 2f–l) and strong cytotoxic functions (Fig. 1j), ^Allo15^BCAR-NKT cells displayed superior tumor cell killing efficacy compared with BCAR-T cells against both primary patient samples (Fig. 2e) and tumor cell lines (Fig. 2i), which was associated with upregulation of activation markers (that is, CD69) and production of cytotoxic molecules (that is, Perforin and Granzyme B) and effector cytokines (that is, IFNγ) (Fig. 2m,n and Supplementary Fig. 7g,h).

### In vivo antitumor efficacy and PK–PD of ^Allo^CAR-NKT cells

**Fig. 3:**
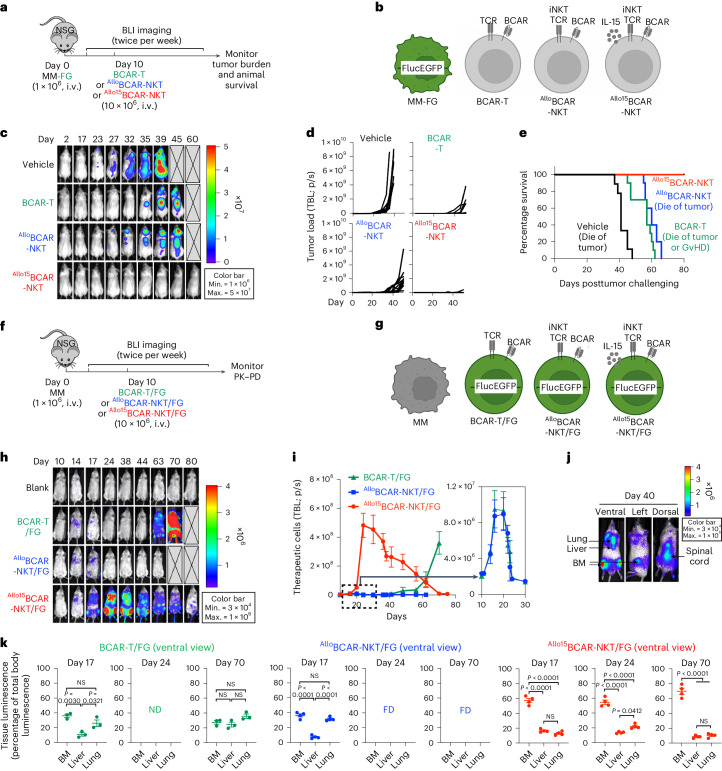
^Allo^CAR-NKT cells exhibit potent antitumor efficacy in vivo, associated with effective tumor homing, expansion and long-term persistence. **a**–**e**, Studying the in vivo antitumor efficacy of ^Allo15^BCAR-NKT cells in a human MM xenograft NSG mouse model. MM-FG indicates MM.1S tumor cell line labeled with the FG dual reporters. **a**, Experimental design. **b**, Schematics showing the tumor cell line and the therapeutic cells used in the study. **c**, BLI images showing the presence of tumor cells in experimental mice over time. **d**, Quantification of **c** (Vehicle, *n* = 9; BCAR-T and ^Allo/15^BCAR-NKT, *n* = 10). TBL, total body luminescence. p/s, photons per second. **e**, Kaplan–Meier survival curves of experimental mice over time (Vehicle, *n* = 9; BCAR-T and ^Allo/15^BCAR-NKT, *n* = 10). **f**–**k**, Studying the in vivo PK–PD of ^Allo15^BCAR-NKT cells in a human MM xenograft NSG mouse model. MM indicates MM.1S tumor cell line. Note the therapeutic cells, but not the MM.1S tumor cell line, were labeled with the FG dual reporters. **f**, Experimental design. **g**, Schematics showing the tumor cell lines and the therapeutic cells used in the PK–PD study. **h**, BLI images showing the presence of therapeutic cells in experimental mice over time. **i**, Quantification of **h** (BCAR-T/FG, *n* = 3; ^Allo/15^BCAR-NKT/FG, *n* = 4). **j**, BLI images showing the biodistribution of ^Allo15^BCAR-NKT cells in a representative experimental mouse. Ventral, left and dorsal views are shown. **k**, Quantification of therapeutic cell tissue distribution (BCAR-T/FG, *n* = 3; ^Allo/15^BCAR-NKT/FG, *n* = 4). Representative of three experiments. Data are presented as the mean ± s.e.m. and were analyzed by one-way ANOVA (**k**). min., minimum; max., maximum; ND, non-detectable; FD, found dead. Source data

To investigate the in vivo antitumor efficacy and PK–PD of ^Allo/15^BCAR-NKT cells, we conducted a pair of mirror-imaging experiments using a human MM xenograft NSG (NOD scid gamma) mouse model (Fig. 3)^18^. Conventional BCAR-T cells were included as a benchmark control. In the efficacy experiment, MM tumor cells were labeled with firefly luciferase and enhanced green fluorescence protein (FG) dual reporters (Fig. 3a-e), while in the PK–PD experiment, therapeutic cells were labeled with the FG dual reporters (Fig. 3f–k and Supplementary Fig. 8a,b). This experimental setup enabled simultaneous visualization of the in vivo dynamics and biodistribution of both tumor cells and therapeutic cells during the same antitumor response.

Under a heavy tumor load condition^18^, a single administration of ^Allo15^BCAR-NKT cells achieved tumor elimination in most (nine out of ten) experimental mice and long-term (>80 days) survival of all experimental mice (Fig. 3c–e). In contrast, both BCAR-T and ^Allo^BCAR-NKT cells only achieved partial suppression of tumor growth and limited improvement of survival (Fig. 3c–e). The experimental mice treated with BCAR-T cells died of a combination of tumor growth and GvHD (Fig. 3e)^18^.

In the PK–PD study under the same heavy tumor load condition, ^Allo15^BCAR-NKT/FG cells expanded more than 200-fold, peaking around week 2, followed by a gradual decline and a long-term persistence greater than 80 days (Fig. 3h,i). Additionally, these cells also exhibited strong tumor homing capacity, effectively and preferentially homing to the major MM tumor site, the BM, during both their peak response and extended persistence (Fig. 3j,k). This homing capacity likely contributes to their superior response rate (Fig. 3c–e). In the absence of IL-15 enhancement, the ^Allo^BCAR-NKT/FG cells showed a moderate fivefold expansion, peaking around week 1, followed by a rapid decline and a greatly shortened persistence of around 10 days (Fig. 3h,i,k). Conventional BCAR-T/FG cells displayed a moderate expansion and a rapid decline similar to those of ^Allo^BCAR-NKT/FG cells in the first 40 days (Fig. 3h,i), which is likely attributed to the stimulation driven by tumor antigens^2^. However, after 40 days (about 1 month after BCAR-T/FG adoptive transfer), BCAR-T/FG cells underwent a swift expansion and infiltrated into various major organs, leading to serious GvHD and mouse demise (Fig. 3h–k), consistent with observations in many CAR-T cell xenograft models^18,36,37^.

### In vivo gene profile of ^Allo^CAR-NKT cells

**Fig. 4:**
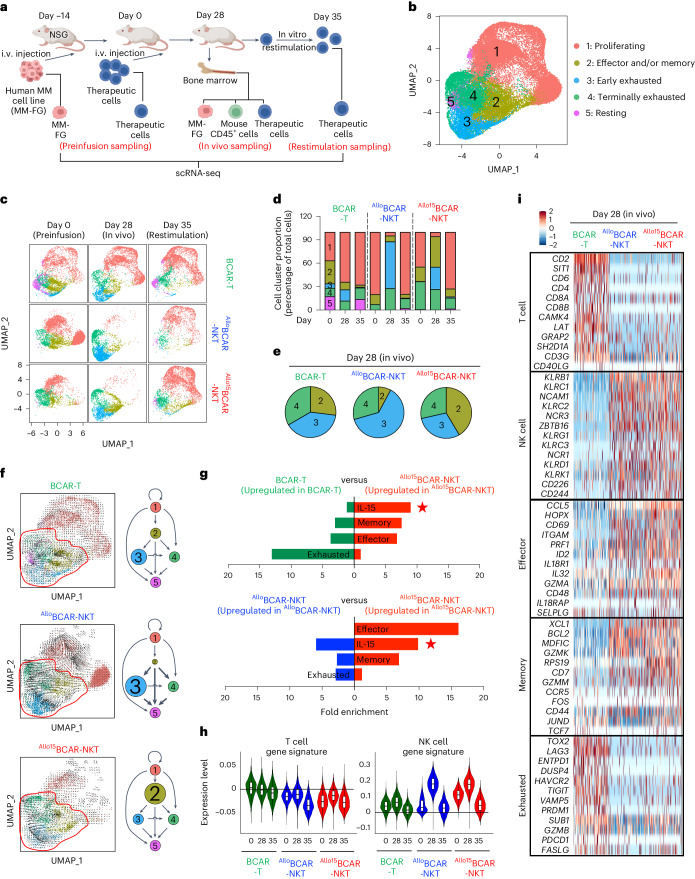
^Allo^CAR-NKT cells display an in vivo gene profile associated with mixed T/NK cell features, strong effector and/or memory function and attenuated exhaustion property. **a**, Schematic showing the experimental design to study the gene profiling of ^Allo15^BCAR-NKT cells using scRNA-seq. Conventional BCAR-T cells and non-IL-15 engineered ^Allo^BCAR-NKT were included as two controls. Therapeutic cell samples were collected at three time points: day 0 (preinfusion samples), day 28 (in vivo samples) and day 35 (in vitro restimulated samples), followed by FACS and scRNA-seq analyses. **b**, Combined UMAP plot showing the formation of five major cell clusters. Total cells combined from all samples are included. Each dot represents a single cell and is colored according to its cell cluster assignment. **c**, Individual UMAP plots showing cell cluster composition of the indicated samples. Each dot represents a single cell and is colored according to its cell cluster assignment. **d**, Bar graphs showing the cell cluster proportions of the indicated samples. **e**, Pie charts showing the cell cluster proportions of the day 28 samples, focusing on cell clusters 2, 3 and 4. **f**, RNA-velocity projected on UMAP plots. Arrows represent the estimates of local average velocity, showing the path (indicated by arrow orientation) and pace (indicated by arrow length) of cell transition. Diagrams summarizing the UMAP cell clustering and RNA-velocity analysis are also presented. The cell cluster proportions are represented by the sizes of circles, while the transitions between cell clusters are symbolized using connecting arrows. **g**, Pathway analyses of differentially expressed genes comparing ^Allo15^BCAR-NKT cells with BCAR-T cells (top) or ^Allo^BCAR-NKT cells (bottom). **h**, Violin plots showing the expression distribution of T and NK cell signature genes in the indicated therapeutic cell samples. **i**, Heatmap showing the expression of representative signature genes. Each column indicates an individual cell. Each row indicates an individual gene. The experiment was performed once; cells isolated from ten mice of each experimental group were combined for analyses. Source data

To study the genomic and molecular regulation of the in vivo antitumor performance of ^Allo/15^BCAR-NKT cells, we used a human MM xenograft NSG mouse model in combination with single-cell RNA sequencing (scRNA-seq) analyses (Fig. 4a). Conventional BCAR-T cells were included as a benchmark control. Three types of therapeutic cell (^Allo15^BCAR-NKT, ^Allo^BCAR-NKT and BCAR-T cells) were collected at three time points (days 0, 28 and 35) followed by scRNA-seq analysis (Fig. 4a). Day 0 samples were collected before infusion, representing the starting status of the therapeutic cells. Day 28 samples were collected from the BM tumor sites of the experimental mice, representing the in vivo tumor-responding status of the therapeutic cells. Day 35 samples were generated by stimulating day 28 samples in vitro with BCMA-expressing aAPCs for 1 week, representing the reactivation potential of the therapeutic cells posttumor exposure (Fig. 4a).

Uniform manifold approximation and projection (UMAP) analysis of combined samples revealed the formation of five major cell clusters (Fig. 4b). The signature gene profiling and gene set enrichment analysis identified cluster 1 cells as proliferating cells, cluster 2 cells as effector and/or memory cells, cluster 3 cells as early exhausted cells, cluster 4 cells as terminally exhausted cells and cluster 5 cells as resting cells (Fig. 4b and Supplementary Fig. 9a,b)^38–45^.

All three types of therapeutic cell displayed large gene profile changes when comparing their day 0 preinfusion samples with their day 28 in vivo samples, suggesting an in vivo genomic and/or molecular signature of a cell product that cannot be fully captured by analyzing its preinfusion product (Fig. 4c,d), consistent with multiple preclinical and clinical studies^46,47^. Day 35 restimulated ^Allo/15^BCAR-NKT cells exhibited a tumor cell killing capacity similar to their day 0 preinfusion counterparts, in contrast to the reduced tumor cell killing capability observed in day 35 restimulated BCAR-T cells (Fig. 2i left panel and Supplementary Fig. 10a,b). This disparity aligns with a better-preserved effector and/or memory cell population (cluster 2) observed in restimulated ^Allo/15^BCAR-NKT cells, compared with that observed in the restimulated BCAR-T cells (Fig. 4c,d). These results indicate that allogeneic CAR-NKT cells, despite their prolonged in vivo exposure to tumors, retain substantial potential for reactivation that may result in sustained therapeutic benefits^48^.

We conducted detailed analyses of the day 28 in vivo samples. Our PK–PD study indicated that day 28 falls in the declining phase of ^Allo/15^BCAR-NKT cell expansion and in the initiation phase of the second BCAR-T cell expansion, likely due to GvHD (Fig. 3h,i). Accordingly, BCAR-T cells contained a notable population of proliferating cells (cluster 1), which was less prevalent in ^Allo/15^BCAR-NKT cells (Fig. 4c,d). When we focused on analyzing the non-proliferating cell populations (clusters 2, 3 and 4), compared with BCAR-T and ^Allo^BCAR-NKT cells, ^Allo15^BCAR-NKT cells contained more effector and memory cells (cluster 2), fewer early exhausted cells (cluster 3) and a similar fraction of terminally exhausted cells (cluster 4) (Fig. 4e). In support of this notion, RNA-velocity analysis revealed the accumulation of ^Allo15^BCAR-NKT cells at the effector and/or memory stage, while BCAR-T and particularly ^Allo^BCAR-NKT cells showed retention at the early exhausted stage (Fig. 4f).

Pathway and gene signature analyses showed that compared with BCAR-T cells, ^Allo15^BCAR-NKT cells displayed enhanced effector and memory, and attenuated exhaustion gene profiles (Fig. 4g,i), aligning with their superior in vivo antitumor efficacy (Fig. 3c–e). These features are likely attributed to IL-15 expression, as they were absent from non-IL-15-engineered ^Allo^BCAR-NKT cells (Figs. 3c–e and 4g,i)^15,22,30,49^. Consequently, an augmented IL-15 signaling pathway was observed in ^Allo15^BCAR-NKT cells but not in BCAR-T or ^Allo^BCAR-NKT cells (Fig. 4g).

Nevertheless, there are fundamental differences between the allogeneic CAR-NKT and conventional CAR-T cell platforms. In comparison to BCAR-T cells, both ^Allo^BCAR-NKT and ^Allo15^BCAR-NKT cells exhibited fewer T cell features and markedly elevated NK cell features, particularly in the in vivo day 28 samples (Fig. 4h,i). The NK cell features of ^Allo/15^BCAR-NKT cells were reflected in their heightened expression of NKRs and robust NKR-mediated tumor cell killing (Fig. 2k,l and Supplementary Fig. 7d,f). BCAR-T cells acquired certain NK cell feature in vivo (Fig. 4h), consistent with a previous study reporting an NK-like transition and dysfunction in CAR-T cells subjected to repetitive tumor stimulation^46^. Conversely, in vivo augmentation of NK features in ^**Allo**^CAR-NKT cells did not correlate with signs of exhaustion (Fig. 4e,g,i).

### Antagonize tumor immune evasion by ^Allo^CAR-NKT cells

**Fig. 5:**
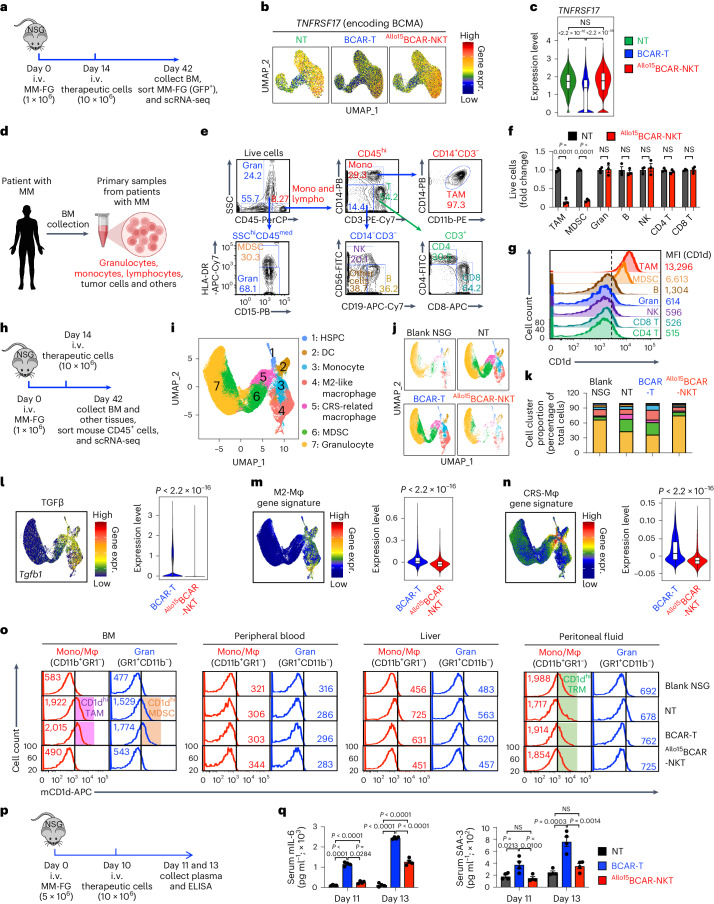
^Allo^CAR-NKT cells block tumor antigen escape, alter TME and exhibit low CRS characteristics. **a**–**c**, Studying the in vivo gene profile of MM tumor cells using scRNA-seq. **a**, Experimental design. **b**, UMAP plots showing the expression distribution of *TNFRSF17* gene encoding BCMA in the indicated samples. NT, no therapeutic cell treatment. **c**, Violin plot showing *TNFRSF17* gene expression. **d**–**g**, Studying the TME targeting using primary samples from patients with MM. **d**, Schematics showing the collection of primary samples from patients with MM. **e**, FACS analysis of immune cell composition. Gran, granulocyte; Mono, monocytes; Lympho, lymphocytes. **f**,**g**, In vitro culturing of samples from patients with MM with or without ^Allo15^BCAR-NKT cells for 24 h. NT, no ^Allo15^BCAR-NKT cell coculture. **f**, Killing data of the indicated TME cell component (*n* = 3). **g**, FACS measurement of surface CD1d expression in the indicated TME cell component. **h**–**o**, Studying the TME targeting using an in vivo human MM xenograft mouse model. **h**, Experimental design. **i**, Combined UMAP plot showing the formation of seven major cell clusters. DC, dendritic cell. **j**, Individual UMAP plots showing cell clusters of the indicated BM samples. **k**, Bar graphs showing the cell cluster proportions of the indicated samples. **l**–**n**, Expression (expr.) of *Tgfb1* (**l**), M2-like Mφ gene signature (**m**) and CRS-related Mφ gene signature (**n**) in the indicated samples. **o**, FACS analyses of CD1d expression on mouse granulocytes and monocytes/macrophages. TRM, tissue-resident macrophage. **p**,**q**, Studying CRS response using an in vivo human MM xenograft mouse model. **p**, Experimental design. **q**, ELISA analyses of mouse IL-6 and SAA-3 in mouse serum (*n* = 4). SAA-3, Serum amyloid A-3. Representative of one (**a**–**c** and **h**–**n**) and three (**d**–**g** and **o**–**q**) experiments. For scRNA-seq, cells isolated from ten mice were combined for analyses. Data are presented as the mean ± s.e.m. and were analyzed by two-tailed Student’s *t*-test (**f**) or one-way ANOVA (**q**). *P* values of violin plots were determined by two-tailed Wilcoxon rank sum test (**l**–**n**) or Kruskal–Wallis test for the overall comparison and Dunn’s test for post hoc pairwise comparisons between groups (**c**). In the violin plots, box and whisker plots exhibit the minimum, lower quartile, median, upper quartile and maximum expression levels of each type of cell. Source data

In addition to analyzing the in vivo gene profiles of therapeutic cells, we conducted scRNA-seq analyses of the MM tumor cells collected from the same in vivo experiment (Figs. 4a and 5a). Our primary focus was to investigate tumor immune evasion, in particular the loss of CAR antigen, which is considered an obstacle for CAR-directed cell therapy^50^.

We observed a reduction of BCMA antigen expression in tumors treated with BCAR-T cells (Fig. 5b,c and Supplementary Fig. 11), a phenomenon that has been reported in patients with MM under BCAR-T cell therapy^51^. In contrast, no BCMA antigen loss was observed in tumors treated with ^Allo15^BCAR-NKT cells (Fig. 5b,c and Supplementary Fig. 11), which could be attributed to the multiple tumor-targeting mechanisms employed by ^Allo15^BCAR-NKT cells (Fig. 2).

### ^Allo^CAR-NKT cells target the TME

The immunosuppressive tumor microenvironment (TME) is considered a notable barrier hindering cancer immunotherapy, particularly in the context of solid tumors, but also for certain blood cancers such as MM, especially when the disease involves BM sites^52–54^. Tumor-associated macrophages and myeloid-derived suppressor cells (MDSCs), two major components of the immunosuppressive TME^54^, both express high levels of CD1d, making them direct targets of NKT cells^54^. We studied the interplay between ^Allo15^BCAR-NKT cells and the MM TME using primary samples from patients with MM (Fig. 5d) and an in vivo MM NSG xenograft model (Fig. 5h).

In the first study, we cocultured ^Allo15^BCAR-NKT cells with primary BM samples from patients with MM (Fig. 5d,e). ^Allo15^BCAR-NKT cells effectively and selectively depleted tumor-associated macrophages and MDSCs, corresponding to the high CD1d expression on these cells (Fig. 5f,g). ^Allo15^BCAR-NKT cells spared other immune cell populations expressing no or low levels of CD1d, including granulocytes, T cells, B cells, NK cells and HSPCs (Fig. 5f,g). Further studies focused on the subpopulations of HSPCs isolated from either BM samples from patients with MM or G-CSF-mobilized healthy donor leukopaks, including long-term HSPCs, short-term HSPCs and multi-potent progenitor cells, showing that all three HSPC subpopulations expressed undetectable CD1d and were not subjected to ^Allo15^BCAR-NKT cell killing (Supplementary Fig. 12a–j).

In the second study, we administered ^Allo15^BCAR-NKT cells into NSG mice bearing human MM xenografts, and collected BM cells and other tissues after 28 days for analysis (Fig. 5h). BM cells collected from tumor-free NSG mice (blank NSG), non-treated tumor-bearing NSG mice (NT) and BCAR-T cell-treated tumor-bearing NSG mice (BCAR-T) were included as controls (Fig. 5h). In this xenograft mouse model, the presence of cross-species reactivity between the human iNKT TCR and mouse CD1d allowed us to study the ^Allo15^BCAR-NKT cell in vivo targeting of mouse CD1d-positive cells within the TME as well as across other tissues^55^. scRNA-seq analysis of BM samples revealed the formation of seven major mouse cell clusters, encompassing HSPCs, dendritic cells (DCs), monocytes, M2-like macrophages, CRS-related macrophages, MDSCs and granulocytes (Fig. 5i and Supplementary Fig. 13). Engraftment of human MM cells led to an increase in the proportion of TME signature cells, such as the M2-like macrophages, CRS-related macrophages and MDSCs (Fig. 5j,k)^54,56–58^. ^Allo15^BCAR-NKT cell treatment altered the TME cell composition, selectively depleting TME signature cells while sparing other cells (that is, HSPCs, DCs, monocytes and granulocytes) (Fig. 5j–n). All three mouse HSPC subpopulations were preserved, including long- and short-term HSPCs and multi-potent progenitor cells (Supplementary Fig. 12k–m). In contrast, the BCAR-T cell treatment exacerbated a more hostile TME, reflected by a further enrichment of TME signature cells (Fig. 5j–n).

The selective ^Allo15^BCAR-NKT cell targeting of BM TME signature cells (M2-like macrophages, CRS-related macrophages and MDSCs) was correlated with their high expression of CD1d, as evidenced by flow cytometry analyses (Fig. 5o). ^Allo15^BCAR-NKT cells did not deplete residential monocytes and/or macrophages and granulocytes from other tissues (that is, peripheral blood, liver and peritoneal fluid), including the CD1d^hi^ tissue-resident macrophages found in the peritoneal fluid (Fig. 5o). This feature may arise from the preferential localization of ^Allo15^BCAR-NKT cells to tumor sites (Fig. 3h,j,k), along with the absence of pronounced CD1d expression on quiescent monocytes, macrophages and/or granulocytes^52^.

### Safety of ^Allo^CAR-NKT cells

CRS-related macrophages have been reported to exacerbate CRS^59,60^. Our scRNA-seq study identified a CRS-related macrophage population (cluster 5) in the BM of MM-engrafted mice (Fig. 5i). Treatment with ^Allo15^BCAR-NKT cells, but not BCAR-T cells, effectively depleted this CRS-related macrophage population in the BM (Fig. 5j,k,n) and reduced CRS-related biomarker (that is, IL-6 and SAA-3) measurements in the serum (Fig. 5p,q)^59,60^.

**Fig. 6:**
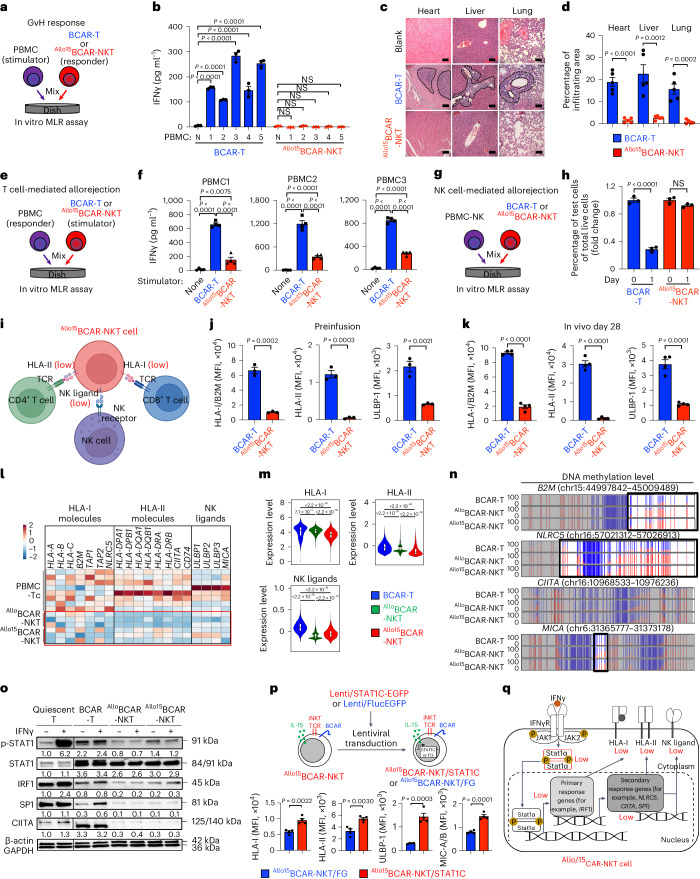
^Allo^CAR-NKT cells exhibit low GvHD risk and high resistance to allorejection. **a**,**b**, Studying the GvH response using an in vitro MLR assay. **a**, Experimental design. **b**, ELISA analyses of IFNγ production on day 4. N, no addition of stimulator PBMCs. **c**,**d**, Studying the GvHD risk of ^Allo15^BCAR-NKT cells using a human MM xenograft mouse model. Experimental design is shown in Fig. 3a. **c**, Hematoxylin and eosin-stained tissue sections. Tissues were collected on day 60. Scale bars, 100 μm. **d**, Quantification of **c** (*n* = 5). **e**,**f**, Studying the T cell-mediated allorejection. **e**, Experimental design. **f**, ELISA analyses of IFNγ production on day 4 (*n* = 4). **g**,**h**, Studying the NK cell-mediated allorejection. **g**, Experimental design. **h**, FACS quantification of the indicated live cells (*n* = 3). **i**–**q**, Studying the molecular mechanisms underlying the allorejection resistance of ^Allo15^BCAR-NKT cells. **i**, Illustration depicting the hypoimmunogenecity working model. **j**–**k**, FACS measurements of surface HLA-I/II and ULBP-1 on ^Allo15^BCAR-NKT cells before infusion (**j**; *n* = 3) and post-in vivo antitumor response as described in Fig. 4a (**k**; *n* = 4). **l**, Bulk RNA-seq analyses of the cells before infusion (*n* = 3). **m**, scRNA-seq analysis of the cells collected from MM xenograft mice on day 28 as described in Fig. 4a. **n**, Methyl-seq analyses of the indicated cells. Vertical lines denote individual CpG sites, wherein red signifies methylation and blue signifies unmethylation. Differentially methylated regions are framed with black boxes. **o**, Western blot analysis of key molecules involved in the IFNγ signaling pathway. **p**, Rescue experiment by overexpressing constitutively active STAT1 (STAT1C) in ^Allo15^BCAR-NKT cells and detecting their surface HLA-I/II and NK ligand expression (*n* = 4). **q**, Schematic illustrating a desensitized IFNγ signaling network within ^Allo15^BCAR-NKT cells. Representative of one (**l**–**n**) or three (**a**–**k** and **o**–**q**) experiments. Data are presented as the mean ± s.e.m. and were analyzed by two-tailed Student’s *t*-test (**d**, **h**, **j**, **k** and **p**) or one-way ANOVA (**b** and **f**). *P* values of violin plots were determined by the Kruskal–Wallis test for the overall comparison, and Dunn’s test for post hoc pairwise comparisons between groups (**m**). In the violin plots, box and whisker plots exhibit the minimum, lower quartile, median, upper quartile and maximum expression levels of each type of cell. Source data

To evaluate GvHD risk, we used a traditional in vitro mixed lymphocyte reaction (MLR) assay (Fig. 6a) as well as an in vivo MM xenograft model (Fig. 3a). In the in vitro MLR assay, ^Allo15^BCAR-NKT cells were stimulated with irradiated PBMCs from 15 mismatched healthy donors (Fig. 6a,b). Minimal IFNγ production was detected, whereas BCAR-T cells vigorously produced IFNγ (Fig. 6b).

In the in vivo MM NSG xenograft model, BCAR-T cells elicited severe GvHD, leading to the death of experimental animals associated with the pronounced immune cell infiltration of vital organs such as the heart, liver and lung (Figs. 3e and 6c,d). In contrast, ^Allo15^BCAR-NKT cell treatment achieved GvHD-free, long-term survival of experimental animals, with no signs of immune cell infiltration in vital organs (Figs. 3e and 6c,d).

### Hypoimmunogenicity of ^Allo^CAR-NKT cells

To evaluate allorejection of ^Allo15^BCAR-NKT cells, we conducted two in vitro MLR assays (Fig. 6e,g). The first MLR assay was designed to study T cell-mediated allorejection, wherein irradiated ^Allo15^BCAR-NKT cells were cocultured with PBMCs from 15 mismatched healthy donors, followed by measuring IFNγ production (Fig. 6e). Compared with BCAR-T cells, ^Allo15^BCAR-NKT cells induced lower production of IFNγ (Fig. 6f). The second MLR assay was designed to study NK cell-mediated allorejection, wherein ^Allo15^BCAR-NKT cells were cocultured with PBMC-NK cells from ten mismatched healthy donors, followed by quantifying viable ^Allo15^BCAR-NKT cells (Fig. 6g). Compared with BCAR-T cells, ^Allo15^BCAR-NKT cells showed greatly improved survival (Fig. 6h). Notably, while HSPC-engineered NKT cells were similar to endogenous PBMC-NKT cells in terms of displaying low GvHD risk, their distinctive characteristic of resistance to allorejection is unique, because the endogenous PBMC-NKT cells induced high production of IFNγ by donor-mismatched PBMCs in the first MLR assay, and were killed by donor-mismatched PBMC-NK cells in the second MLR assay (Supplementary Fig. 14).

To investigate the mechanisms responsible for the unique allorejection resistance of ^Allo15^BCAR-NKT cells, we started by examining cell surface molecules associated with allorejection^18^. Compared with BCAR-T cells, ^Allo15^BCAR-NKT cells expressed lower levels of surface HLA-I, HLA-II and NK ligands (that is, ULBP-1) in both in vitro cell cultures and in vivo antitumor studies (Fig. 6i–k). RNA-seq analyses revealed a sustained low expression of gene sets encoding HLA-I/II molecules and NK ligands (Fig. 6l,m), indicating an intrinsic and stable hypoimmunogenic phenotype of ^Allo15^BCAR-NKT cells that is regulated at the transcriptional level. Next, we studied epigenetic regulation and signaling networks that govern transcription of the pertinent genes.

Chromatin remodeling processes regulate transcription of HLA and NK ligand genes^61,62^. Genome-wide methylation sequencing (methyl-seq) analysis of ^Allo15^BCAR-NKT cells revealed hypermethylation in the promoter regions of many HLA and NK ligand genes (Fig. 6n), suggesting that epigenetic regulation may contribute to the hypoimmunogenic phenotype of ^Allo15^BCAR-NKT cells.

We examined the IFNγ-JAK-STAT1 signaling pathway, one of the best-characterized pathways regulating HLA and NK ligand gene expression^63,64^. Compared with PBMC-T and BCAR-T cells, ^Allo15^BCAR-NKT cells exhibited a desensitized IFNγ-JAK-STAT1 signaling, characterized by low STAT1 phosphorylation at both basal level and after IFNγ stimulation (Fig. 6o). Correspondingly, transcription factors encoded by IFNγ primary responsive genes (for example, *IRF1*) and secondary responsive genes (for example, *CIITA* and *SP-1*) were also maintained at low levels (Fig. 6o). IRF1 regulates *HLA-I* gene expression^65^, CIITA regulates *HLA-II* expression^66^ and SP-1 regulates NK ligands (for example, ULBP)^67^. Overexpression of a constitutively active STAT1 molecule^68^ partially reversed the hypoimmunogenic phenotype of ^Allo15^BCAR-NKT cells, evidenced by their upregulation of surface HLA-I/II and NK ligands (Fig. 6p).

Collectively, these findings reveal a hypoimmunogenic phenotype that is regulated by epigenetic and signaling network mechanisms (Fig. 6q). This hypoimmunogenic phenotype is intrinsic to our allogeneic HSPC-engineered NKT cells, and is stable throughout inflammation (for example, IFNγ stimulation) and in vivo antitumor responses.

### Comparison of HSPC- and PBMC-derived CAR-NKT cells

**Fig. 7:**
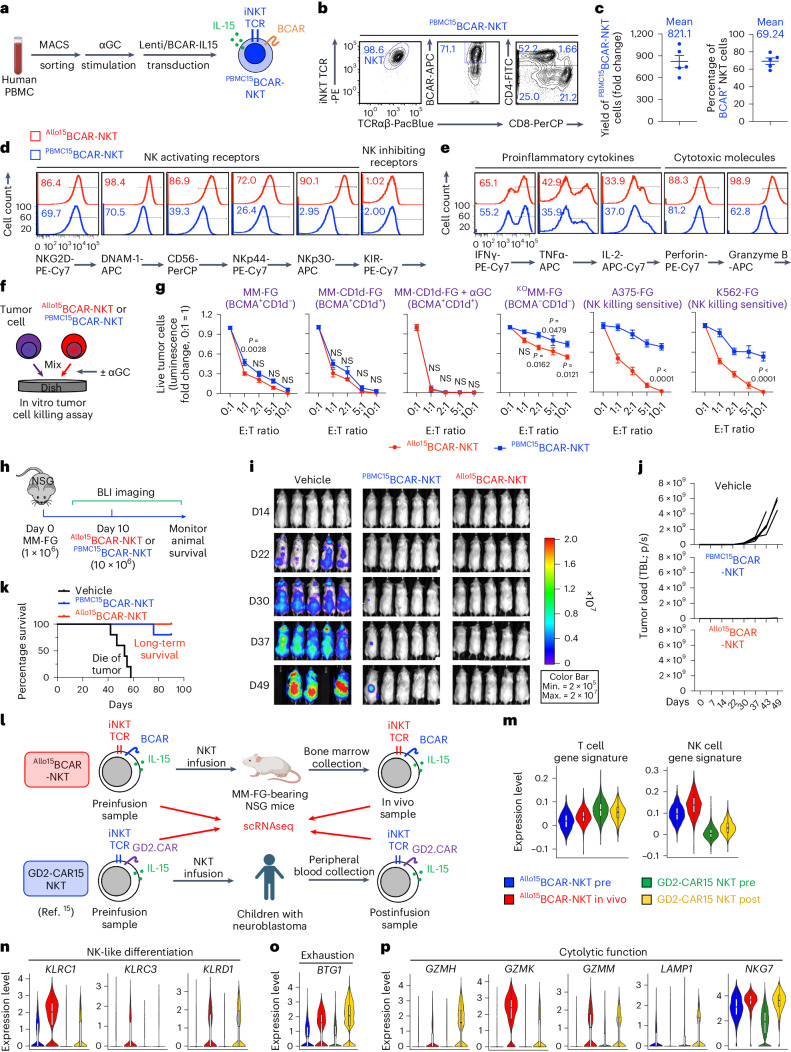
^Allo^CAR-NKT cells demonstrate similar antitumor capacity to PBMC-derived CAR-NKT cells but with heightened NK attributes. **a**–**j**, Comparison between HSPC-derived ^Allo15^BCAR-NKT cells and PBMC-derived IL-15-enhanced BCAR-NKT (^PBMC15^BCAR-NKT) cells. **a**, Schematics showing the generation of ^PBMC15^BCAR-NKT cells. **b**, FACS plots showing the purity, CAR expression and CD4/CD8 coreceptor expression of ^PBMC15^BCAR-NKT cells. **c**, Yield and CAR expression of ^PBMC15^BCAR-NKT cells (*n* = 5; *n* indicates different donors). **d**, FACS plots showing the surface expression of NKRs on ^Allo15^BCAR-NKT and ^PBMC15^BCAR-NKT cells. **e**, FACS plots showing the intracellular production of cytokines and cytotoxic molecules in ^Allo15^BCAR-NKT and ^PBMC15^BCAR-NKT cells. **f**,**g**, Comparing the in vitro antitumor efficacy of ^Allo15^BCAR-NKT and ^PBMC15^BCAR-NKT cells. **f**, Experimental design. **g**, Tumor cell killing data at 24 h (*n* = 4). **h**–**k**, Comparing the in vivo antitumor efficacy of ^Allo15^BCAR-NKT and ^PBMC15^BCAR-NKT cells in a human MM xenograft NSG mouse model. **h**, Experimental design. **i**, BLI images showing the tumor loads in experimental mice over time. **j**, Quantification of **i** (*n* = 5). **k**, Kaplan–Meier survival curves of experimental mice over time (*n* = 5). **l**–**p**, scRNA-seq comparison between ^Allo15^BCAR-NKT cells and PBMC-derived IL-15-enhanced GD2CAR-engineered NKT (GD2-CAR15 NKT) cells. **l**, Schematics showing the experimental design to compare the gene profiling of ^Allo15^BCAR-NKT and GD2-CAR15 NKT cells using scRNA-seq. GD2-CAR15 NKT cell data are obtained from GSE223071. **m**, Violin plots showing the expression distribution of T and NK cell gene signatures in the indicated CAR-NKT cells. **n**–**p**. Violin plots showing the expression distribution of genes involved in NK-like differentiation (**n**), exhaustion (**o**) and cytolytic function (**p**), in the indicated CAR-NKT cells. Representative of three (**a**–**g**), two (**h**–**k**) and one (**l**–**p**) experiments. Data are presented as the mean ± s.e.m. and were analyzed by two-tailed Student’s *t*-test (**g**). In the violin plots, box and whisker plots exhibit the minimum, lower quartile, median, upper quartile and maximum expression levels of each type of cell. Source data

We performed side-by-side comparison of ^Allo15^BCAR-NKT cells and PBMC-derived IL-15-enhanced BCAR-NKT (^PBMC15^CAR-NKT) cells (Figs. 7a–j). Flow cytometry analysis revealed substantial similarities between the two types of BCAR-NKT cell, including their capacity to produce high levels of effector cytokines (that is, IFNγ, TNFα and IL-2) and cytotoxic molecules (that is, Granzyme B and Perforin) (Fig. 7e). However, compared with ^PBMC15^CAR-NKT cells, ^Allo15^BCAR-NKT cells expressed higher levels of NKRs (for example, DNAM-1, NKp44 and NKp30), indicating their enhanced NK feature (Fig. 7d). The antitumor efficacy of both BCAR-NKT cells was assessed using a series of in vitro tumor cell killing assays, as well as a human MM xenograft NSG mouse model (Fig. 7f,h). Both BCAR-NKT cells demonstrated similar antitumor efficacy in killing BCMA^+^ MM tumor cells, both in vitro and in vivo (Fig. 7g–k). Compared with ^PBMC15^CAR-NKT cells, ^Allo15^BCAR-NKT cells exhibited enhanced killing of BCMA^−^ tumor cells, particularly those sensitive to NKR-mediated cytotoxicity (for example, human melanoma cell line A375 and leukemia line K562), aligning with their heightened NKR expressions (Fig. 7d,g).

We integrated the scRNA-seq datasets reported from a recent clinical trial of autologous PBMC-derived GD2.CAR-engineered IL-15-enhanced NKT cells^15^ with those generated from our preclinical animal study evaluating ^Allo15^BCAR-NKT cells (Fig. 4) and compared the gene profiles of these two types of CAR-NKT cell (Fig. 7l). Both populations expressed genes indicative of a mixed T/NK signature (Fig. 7m,n), exhaustion (for example, *BTG1*; Fig. 7o) and cytolytic function (for example, GZMH, *GZMK*, *GZMM*, *LAMP1* and *NKG7*; Fig. 7p), all of which were upregulated after adoptive transfer (Fig. 7m–p). In agreement with the comparison to ^PBMC15^BCAR-NKT cells (Fig. 7a–j), ^Allo15^BCAR-NKT cells demonstrated overall heightened NK gene expression compared with GD2-CAR15 NKT cells (Fig. 7m,n).

## Discussion

To generate ^Allo^CAR-NKT cells compatible with clinical development, we provide a culture system for differentiation and expansion that does not use 3D culture or xenogeneic feeder cells. This technology is versatile, as shown by the successful generation of CAR-NKT cells targeting five different tumor antigens (Fig. 1 and Supplementary Figs. 1, 15 and 16). In addition to CARs, we incorporated other genetic elements into the cells, such as those encoding immune-enhancement molecules (for example, IL-15; Fig. 1d and Supplementary Fig. 1), safety controls (for example, sr39TK; Supplementary Fig. 17)^18,23^ and reporters (for example, Fluc and EGFP; Fig. 3g). None of the evaluated cargo genes appeared to disrupt cell manufacture, yield or quality, suggesting a potential broad application of the technology to target diverse blood cancers, solid tumors and possibly other diseases.

Numerous allogeneic cell therapies have been developed based either on conventional αβ T cells or NK cells, many equipped with CARs^5,6,18,69,70^. In the case of the conventional αβ T cell platform, multiplex gene editing is essential to disrupt the native αβ TCR, involving the disruption of *TRAC* and/or *TRBC* loci, to prevent GvHD induced by HLA incompatibility^4^. Additionally, HLA-I and/or HLA-II molecules are ablated to mitigate rejection by host T cells^4^, and CD52 is disrupted to render these allogeneic T cells resistant to lymphodepleting drugs such as alemtuzumab^71^. The multiplex gene editing increases the risk of aneuploidy and tumorigenicity, and diminishes the cells’ efficacy and persistence in vivo^72,73^. Moreover, ablation of TCR expression compromises cytokine production and long-term persistence compared with CAR-T cells bearing intact TCRs^74–76^. On the other hand, NK cell-based allogeneic cell products are thought to carry a lower risk of GvHD, simplifying the cell engineering^5,77^. Nevertheless, their expansion and effectiveness against tumors in vivo might be more restricted compared with conventional αβ T cells^5,77^. Various allogeneic cell products have undergone phase I clinical trials targeting B cell malignancies, acute myeloid leukemia, MM, as well as certain solid tumors^4,5,78,79^, with promising initial results.

A recent phase I clinical trial evaluated an autologous PBMC-derived GD2.CAR-engineered IL-15-enhanced NKT (GD2-CAR15 NKT) cell product in the treatment of relapsed or refractory neuroblastoma^6,15^. The trial outcomes supported the cells’ safety and efficacy^6,15^. Our finding that ^Allo^CAR-NKT cells have similar phenotype, functionality and molecular characteristics as their PBMC-derived CAR-NKT cell counterparts underscore the clinical potential of ^Allo^CAR-NKT cell therapy (Fig. 7). The heightened NK attributes of ^Allo^CAR-NKT cells compared with PBMC-derived CAR-NKT cells are of interest, particularly for treating solid tumors characterized by loss or heterogeneous expression of the CAR antigen.

Our ^Allo^CAR-NKT cells are free of bystander conventional αβ T cells (Fig. 1e,g), which reduces the risk of GvHD and enables a streamlined manufacturing process without additional purification steps^16^. Because NKT cells do not recognize mismatched HLAs, knockout of the endogenous TCR is unnecessary, thereby preserving its benefits for CAR-NKT cells^74^. Fifteen cord-blood donors of diverse genetic backgrounds were successfully used to generate CAR-NKT cells (Fig. 1 and Supplementary Fig. 18), avoiding the need for donor selections. A more than 10^6^-fold expansion was achieved from the input HSPCs to the output mature NKT cells. Assuming a linear extrapolation during the scale-up production, we estimate that from a single cord-blood donor (typically ~1–10 × 10^6^ CD34^+^ HSPCs), roughly 10^12^ mature ^Allo^CAR-NKT cells could be generated, which could potentially be formulated into 1,000–10,000 doses (of ~10^8^–10^9^ cells per dose following current CAR-T cell therapy dosing practices) (Fig. 1a,c and Supplementary Fig. 2c–e)^80^. Note that the high cell yield was only attained when culturing TCR-engineered HSPCs but not non-TCR-engineered HSPCs, underscoring a pivotal role of TCR engineering in the reported technology (Supplementary Fig. 2f)^81,82^.

An additional concern with allogeneic cell products is host cell-mediated allorejection. We showed that ^Allo^CAR-NKT cells exhibit an intrinsic, stable hypoimmunogenic phenotype, which is underpinned by epigenetic and signaling network regulation (Fig. 6i–q). Surface HLA-I molecules are only around 20–50% of the levels observed on conventional CAR-T cells (Fig. 6j,k). A recent study of HLA-I knockdown in allogeneic CAR-T cell products^74^ suggests that this level of HLA-I reduction confers resistance against both host T and NK cell-mediated allorejection. It is important to note that in vitro MLR assays are models that may not predict allorejection in the clinic. Therefore, alternative strategies, such as humanized in vivo models, are needed to better evaluate allorejection, although definitive evaluation of allorejection can only be accomplished through clinical trials.

Our current ^Allo^CAR-NKT cells comprise mainly CD8 single-positive and/or double-negative cells while missing a CD4 single-positive population that may be of special therapeutic value (Fig. 1e and Supplementary Fig. 1c)^6,15^; future modification of the culture protocol may generate a CD4 single-positive NKT population. Antitumor potency could be improved using genes encoding other immune-enhancing molecules (for example, IL-7, IL-18 and IL-21) and immunosuppression-resistant factors (for example, immune checkpoint inhibitors such as anti-PD-1 antibody, dominant-negative TGFβ receptor)^2^. The safety profile could be further enhanced with suicide switch systems (for example, inducible Cas9 and truncated EGFR)^83,84^. In vivo performance may also be improved through synergism with other therapeutic modalities (for example, checkpoint blockade therapy and preconditioning regimens)^73^. Overall, clinical investigation is needed to fully assess the potential of ^Allo^CAR-NKT cells as allogeneic candidates for off-the-shelf cancer therapy.

## Methods

### Mice

NOD.Cg-Prkdc^SCID^Il2rg^tm1Wjl^/SzJ (NOD/SCID/IL-2Rγ^−/−^, NSG) mice were maintained in the animal facilities of University of California, Los Angeles (UCLA). Here 6–10 week-old female mice were used for all experiments unless otherwise indicated. All animal experiments were approved by the Institutional Animal Care and Use Committee of UCLA. All mice were bred and maintained under specific pathogen-free conditions, and all experiments were conducted in accordance with the animal care and use regulations of the Division of Laboratory Animal Medicine at the UCLA.

### Media and reagents

The X-VIVO 15 Serum-Free Hematopoietic Cell Medium was purchased from Lonza. The StemSpan T Cell Generation Kit, comprising the StemSpan SFEM II Medium, the StemSpan Lymphoid Progenitor Expansion Supplement, the StemSpan Lymphoid Progenitor Maturation Supplement, the StemSpan Lymphoid Progenitor Differentiation Coating Material and the ImmunoCult Human CD3/CD28/CD2 T Cell Activator, was purchased from StemCell Technologies. The CTS OpTmizer T Cell Expansion SFM (no phenol red, bottle format), the Roswell Park Memorial Institute (RPMI) 1640 cell culture medium and the DMEM cell culture medium were purchased from Thermo Fisher Scientific. The CryoStor Cell Cryopreservation Media CS10 was purchased from MilliporeSigma.

α-Galactosylceramide (αGC, KRN7000) was purchased from Avanti Polar Lipids. Recombinant human IL-2, IL-3, IL-7, IL-15, IL-21, IFNγ, Flt3 ligand (Flt3L), stem cell factor (SCF) and thrombopoietin (TPO) were purchased from Peprotech. Ganciclovir, fetal bovine serum (FBS) and beta-mercaptoethanol (β-ME) were purchased from Sigma. Penicillin-streptomycin-glutamine (PSG), MEM non-essential amino acids, HEPES buffer solution and sodium pyruvate were purchased from Gibco. Normocin was purchased from Invivogen.

The homemade C10 medium was made of RPMI 1640 cell culture medium, supplemented with FBS (10% vol/vol), PSG (1% vol/vol), MEM non-essential amino acids (1% vol/vol), HEPES (10 mM), sodium pyruvate (1 mM), β-ME (50 mM) and normocin (100 mg ml^−1^). The homemade D10 medium was made of DMEM supplemented with FBS (10% vol/vol), PSG (1% vol/vol) and normocin (100 mg ml^−1^). The homemade R10 medium was made of RPMI supplemented with FBS (10% vol/vol), PSG (1% vol/vol) and normocin (100 mg ml^−1^).

### Lentiviral vectors

Lentiviral vectors used in this study were all constructed from a parental lentivector pMNDW^18^^,^^23^^,^^33^. The 2A sequences derived from foot-and-mouth disease virus (F2A), porcine teschovirus-1 (P2A) and thosea asigna virus (T2A) were used to link the inserted genes to achieve coexpression.

The Lenti/iNKT vector was constructed by inserting into the pMNDW parental vector a synthetic bicistronic gene encoding human iNKT TCRα-F2A-TCRβ (refs. ^18,23^). The Lenti/iNKT-sr39TK vector was constructed by inserting a synthetic tricistronic gene encoding human iNKT TCRα-F2A-TCRβ-P2A-sr39TK. The Lenti/iNKT-BCAR vector was constructed by inserting a synthetic tricistronic gene encoding human iNKT TCRα-F2A-TCRβ-P2A-BCAR (BCAR indicates a BCMA-targeting CAR)^24^. The Lenti/iNKT-BCAR-IL15 vector was constructed by inserting a synthetic tetracistronic gene encoding human iNKT TCRα-F2A-TCRβ-P2A-BCAR-T2A-IL15 (IL15 indicates the secreted form of human IL-15). The Lenti/iNKT-CAR19 vector was constructed by inserting a synthetic tricistronic gene encoding human iNKT TCRα-F2A-TCRβ-P2A-CAR19 (CAR19 indicates a CD19-targeting CAR)^25^. The Lenti/iNKT-CAR19-IL15 vector was constructed by inserting a synthetic tetracistronic gene encoding human iNKT TCRα-F2A-TCRβ-P2A-CAR19-T2A-IL15. The Lenti/iNKT-GD2.CAR vector was constructed by inserting a synthetic tricistronic gene encoding human iNKT TCRα-F2A-TCRβ-P2A-GD2.CAR (GD2.CAR indicates a GD2-targeting CAR)^26^. The Lenti/iNKT-GD2.CAR-IL15 vector was constructed by inserting a synthetic tetracistronic gene encoding human iNKT TCRα-F2A-TCRβ-P2A-GD2.CAR-T2A-IL15. The Lenti/iNKT-GPC3.CAR vector was constructed by inserting a synthetic tricistronic gene encoding human iNKT TCRα-F2A-TCRβ-P2A-GPC3.CAR (GPC3CAR indicates a glypican-3-targeting CAR)^27^. The Lenti/iNKT-GPC3.CAR-IL15 vector was constructed by inserting a synthetic tetracistronic gene encoding human iNKT TCRα-F2A-TCRβ-P2A-GPC3.CAR-T2A-IL15. The Lenti/iNKT-ECAR vector was constructed by inserting a synthetic tricistronic gene encoding human iNKT TCRα-F2A-TCRβ-P2A-ECAR (ECAR indicates an epidermal growth factor receptor variant III-targeting CAR)^28^. The Lenti/iNKT-ECAR-IL15 vector was constructed by inserting a synthetic tetracistronic gene encoding human iNKT TCRα-F2A-TCRβ-P2A-ECAR-T2A-IL15. The Lenti/iNKT-IL15-sr39TK vector was constructed by inserting a synthetic tetracistronic gene encoding human iNKT TCRα-F2A-TCRβ-P2A-IL15-T2A-sr39TK (sr39TK indicates an sr39TK suicide and positron emission tomography imaging reporter gene).

The Lenti/FlucGFP vector was constructed by inserting into the pMNDW a synthetic bicistronic gene encoding Fluc-P2A-EGFP (ref. ^23^). The Lenti/CD1d vector was constructed by inserting a synthetic gene encoding human CD1d (ref. ^23^). The Lenti/EGFRVIII vector was constructed by inserting a synthetic gene encoding human EGFRvIII. The Lenti/STAT1C-EGFP vector was constructed by inserting a synthetic bicistronic gene encoding STAT1C-P2A-EGFP (STAT1C indicates a human constitutively active STAT1, ref. ^85^). The Lenti/BCAR vector was constructed by inserting a synthetic gene encoding BCAR. The Lenti/BCAR-IL15 vector was constructed by inserting a synthetic bicistronic gene encoding BCAR-F2A-IL15. The Lenti/CAR19 vector was constructed by inserting a synthetic gene encoding CAR19. The Lenti/GD2.CAR vector was constructed by inserting a synthetic gene encoding GD2.CAR. The Lenti/GPC3.CAR vector was constructed by inserting a synthetic gene encoding GPC3.CAR. The Lenti/ECAR vector was constructed by inserting a synthetic gene encoding ECAR.

The synthetic gene fragments were obtained from GenScript and IDT. Lentiviruses were produced using human embryonic kidney (HEK) 293T cells (American Type Culture Collection (ATCC)), following a standard transfection protocol using the Trans-IT-Lenti Transfection Reagent (Mirus Bio) and a centrifugation concentration protocol using the Amicon Ultra Centrifugal Filter Units, according to the manufacturers’ instructions (MilliporeSigma).

### Cell lines

Human MM cell line MM.1S, chronic myelogenous leukemia cell line K562, Burkitt’s lymphoma cell line Raji, acute lymphoblastic leukemia cell line NALM-6, melanoma cell line A375, glioblastoma cell line T98G and U87MG, hepatocellular carcinoma cell line HEP3B and HEK293T were purchased from the ATCC.

To make stable tumor cell lines overexpressing human CD1d or EGFRvIII, and/or FG dual reporters, the parental tumor cell lines were transduced with lentiviral vectors encoding the intended gene(s). Then 72 h after lentivector transduction, cells were subjected to flow cytometry sorting to isolate gene-engineered cells for making stable cell lines. Nine stable tumor cell lines were generated for this study, including MM-FG, MM-FG-CD1d, K562-FG, Raji-FG, Raji-FG-CD1d, NALM-6-FG, T98G-FG, U87MG-EGFRvIII-FG and Hep3B-FG cell lines. The ^KO^MM-FG cell line was generated by knocking out the BCMA gene from the parental MM-FG cell line using CRISPR–Cas9. The single-guide RNA (sgRNA) targeting the BCMA gene (UAUUAAGCUCAGUCCCAAAC) was purchased from Synthego, and was introduced into MM-FG cells via electroporation using an Amaxa 4D Nucleofection X Unit (Lonza), according to the manufacturer’s instructions.

The artificial antigen-presenting cell (aAPC) was generated by engineering the K562 cell line to overexpress human CD83/CD86/4-1BBL costimulatory receptors^86^. The aAPC-BCMA, aAPC-CD19, aAPC-GD2, aAPC-GPC3 and aAPC-EGFRvIII cell lines were generated by further engineering the parental aAPC line to overexpress human BCMA, CD19, GD2, GPC3 and EGFRvIII, respectively.

### Human CD34^+^ HSPCs, PBMCs and primary BM samples from patients with MM

Purified cord-blood-derived human CD34^+^ cells were purchased from the HemaCare. Healthy donor PBMCs were obtained from the UCLA/CFAR Virology Core Laboratory without identification information under federal and state regulations. Primary samples from patients with MM were collected at the Ronald Reagan UCLA Medical Center from consented patients through an IRB-approved protocol (IRB no. 21-001444) and processed.

### Antibodies and flow cytometry

Fluorochrome-conjugated antibodies specific for human CD45 (Clone H130, cat. no. 304026, 1:500 dilution), TCRαβ (Clone I26, cat. no. 306716, 1:25 dilution), CD3 (Clone HIT3a, cat. no. 300329, 1:500 dilution), CD4 (Clone OKT4, cat. no. 317414, 1:400 dilution), CD8 (Clone SK1, cat. no. 344714, 1:500 dilution), CD45RO (Clone UCHL1, cat. no. 304216, 1:200 dilution), CD45RA (Clone HI100, cat. no. 304105, 1:5,000 dilution), CD161 (Clone HP-3G10, cat. no. 339928, 1:50 dilution), CD69 (Clone FN50, cat. no. 310909, 1:50 dilution), CD56 (Clone HCD56, cat. no. 362545, 1:10 dilution), CD1d (Clone 51.1, cat. no. 350308, 1:50 dilution), BCMA (19F2, cat. no. 357503, 1:50 dilution), CD14 (Clone HCD14, cat. no. 325608, 1:100 dilution), CD19 (Clone HIB19, cat. no. 363005, 1:100 dilution), CD11b (Clone ICRF44, cat. no. 301330, 1:500 dilution), CD15 (W6D3, cat. no. 323021, 1:500 dilution), CD112 (Clone TX31, cat. no. 337409, 1:200 dilution), CD155 (Clone SKII.4, cat. no. 337613, 1:200 dilution), MICA/MICB (Clone 6D4, cat. no. 320908, 1:50 dilution), Ganglioside GD2 (14G2a, cat. no. 357323, 1:50 dilution), NKG2D (Clone 1D11, cat. no. 320812, 1:50 dilution), DNAM-1 (Clone 11A8, cat. no. 338312, 1:50 dilution), CD158 (KIR2DL1/S1/S3/S5) (Clone HP-MA4, cat. no. 339510, 1:50 dilution), NKp30 (Clone P30-15, cat. no. 325207, 1:50 dilution), NKp44 (Clone P44-8, cat. no. 325107, 1:50 dilution), CD16 (Clone 3G8, cat. no. 302011, 1:50 dilution), NKG2A (Clone S19004C, cat. no. 375103, 1:50 dilution), NKG2C (Clone S19005E, cat. no. 375003, 1:50 dilution), CD62L (Clone P44-8, cat. no. 304813, 1:50 dilution), CD134 (Clone Ber-ACT35, cat. no. 350008, 1:50 dilution), LEF1 (Clone W17021C, cat. no. 621051, 1:50 dilution), CD8A (Clone C8/144B, cat. no. 372092, 1:500 dilution), CD8B (Clone QA20A40, cat. no. 387305, 1:500 dilution), IFNγ (Clone B27, cat. no. 506518, 1:50 dilution), Granzyme B (Clone QA16A02, cat. no. 372204, 1:4,000 dilution), Perforin (Clone dG9, cat. no. 308126, 1:50 dilution), TNFα (Clone Mab11, cat. no. 502912, 1:4,000 dilution), IL-2 (Clone MQ1-17H12, cat. no. 500341, 1:200 dilution), β2-microglobulin (B2M) (Clone 2M2, cat. no. 316312, 1:5,000 dilution), HLA-DR (Clone L243, cat. no. 307618, 1:250 dilution), and HLA-DR, double-positive, DQ (Clone Tü 39, cat. no. 361707, 1:250 dilution) were purchased from BioLegend. Fluorochrome-conjugated antibodies specific for mouse CD45 (Clone S18009F, cat. no. 157607, 1:5,000 dilution), GR1 (Clone RB6-8C5, cat. no. 108411, 1:1,000 dilution), CD1d (Clone 1B1, cat. no. 123521, 1:100 dilution), CD11b (Clone M1/70, cat. no. 101205, 1:5,000 dilution), Sca1 (Clone D7, cat. no. 108111, 1:100 dilution), Flt3 (Clone A2F10, cat. no. 135305, 1:100 dilution), SLAM (Clone TC15-12F12.2, cat. no. 115913, 1:50 dilution), c-Kit (Clone 2B8, cat. no. 105825, 1:50 dilution) were purchased from BioLegend. In our study, note the use of antibodies with identical clones but differing conjugated fluorochromes, with one typical antibody listed herein. Fluorochrome-conjugated antibody specific for human Glypican-3 (GPC3; Clone 024, cat. no. ab275695, 1:200 dilution) was purchased from Abcam. Fluorochrome-conjugated antibodies specific for human CD34 (Clone 581, cat. no. 555822, 1:100 dilution) and human iNKT TCR Vɑ24-Jβ18 (Clone 6B11, cat. no. 552825, 1:10 dilution) were purchased from BD Biosciences. Fluorochrome-conjugated antibody specific for human iNKT TCR Vβ11 (Clone C21, cat. no. A66905, 1:50 dilution) was purchased from Beckman-Coulter. Fluorochrome-conjugated antibodies specific for human ULBP-1 (Clone 170818, cat. no. FAB1380P, 1:50 dilution) and ULBP-2,5,6 (Clone 165903, cat. no. FAB1298A5, 1:50 dilution) were purchased from R&D Systems. A goat antimouse IgG F(ab’)2 secondary antibody (cat. no. 31803, 1:50 dilution) was purchased from Thermo Fisher. Fixable Viability Dye eFluor506 (e506, cat. no. 65-0866-14, 1:500 dilution) was purchased from Affymetrix eBioscience; mouse Fc Block (antimouse CD16/32, cat. no. 553142, 1:50 dilution) was purchased from BD Biosciences and human Fc Receptor Blocking Solution (TrueStain FcX, cat. no. 422302, 1:100 dilution) was purchased from BioLegend.

All flow cytometry staining was performed following standard protocols, as well as specific instructions provided by the manufacturer of a particular antibody. Stained cells were analyzed using a MACSQuant Analyzer 10 flow cytometer (Miltenyi Biotech), following the manufacturer’s instructions. FlowJo software v.9 (BD Biosciences) was used for data analysis.

### Enzyme-linked immunosorbent cytokine assays (ELISAs)

Supernatants from cell culture assays were collected and assayed to quantify human IFNγ, TNFα, IL-2, and IL-4. The capture and biotinylated pairs for detecting cytokines were purchased from BD Biosciences. The streptavidin–HRP conjugate was purchased from Invitrogen. Human cytokine standards were purchased from eBioscience. Tetramethylbenzidine (TMB) substrate was purchased from KPL. Human IL-15 was quantified using a Human IL-15 Quantikine ELISA Kit (R&D Systems), following the manufacturer’s instructions. Human IL-17a was quantified using a Human IL-17A ELISA MAX Deluxe Kit (BioLegend), following the manufacturer’s instructions. Mouse IL-6 was quantified with paired purified anti-mouse IL-6 antibody and biotin anti-mouse IL-6 antibody (BioLegend). Mouse SAA-3 was quantified using a Mouse SAA-3 ELISA Kit (MilliporeSigma), per the manufacturer’s instructions. The samples were analyzed for absorbance at 450 nm using an Infinite M1000 microplate reader (Tecan).

### Generation of allogeneic HSPC-engineered NKT (^Allo^NKT) cells and their CAR/IL15-armed derivatives (denoted as ^Allo/15^CAR-NKT cells)

^Allo^NKT and ^Allo/15^CAR-NKT cells were generated by differentiating gene-engineered cord-blood CD34^+^ HSPCs in a five-stage Ex Vivo HSPC-Derived NKT Cell Culture. ^Allo^NKT cells were differentiated from HSPCs engineered to overexpress a transgenic human iNKT TCR, while ^Allo/15^CAR-NKT cells were differentiated from HSPCs engineered to overexpress a human transgenic iNKT TCR, together with a selected CAR and/or the secreted form of human IL-15.

At stage 0, 1 × 10^4^ frozen–thawed human CD34^+^ HSPCs were revived and cultured in 300 µl of X-VIVO 15 Serum-Free Hematopoietic Stem Cell Medium supplemented with 50 ng ml^−1^ Flt3L, 50 ng ml^−1^ stem cell factor, 50 ng ml^−1^ thrombopoietin and 20 ng ml^−1^ IL-3 in non-tissue culture treated 24-well plate for 24 h, then transduced with Lenti/iNKT-(CAR)-(IL15) viruses for another 24 h following an established protocol^18,23^. Briefly, concentrated lentivirus supernatant was mixed with 1:100 Poloxamer Synperonic F108 and 1:1,000 Prostaglandin E2 (PGE2; CAYMAN), and then gently added to the HSPCs culture.

At stage 1, gene-engineered HSPCs collected from stage 0 were cultured in the StemSpan SFEM II Medium supplemented with StemSpan Lymphoid Progenitor Expansion Supplement for 2 weeks. CELLSTAR 24-well Cell Culture Non-treated Multiwell Plates (VWR) were used. The plates were coated with 500 µl per well StemSpan Lymphoid Differentiation Coating Material for 2 h at room temperature or alternatively, overnight at 4 °C. Transduced CD34^+^ HSPCs were suspended at 2 × 10^4^ cells per ml and 500 µl of cell suspension was added into each precoated well. Twice per week, half of the medium from each well was removed and replaced with fresh medium.

At stage 2, cells collected from the stage 1 were cultured in the StemSpan SFEM II Medium supplemented with StemSpan Lymphoid Progenitor Maturation Supplement for 1 week. Non-Treated Falcon Polystyrene six-well Microplates (Thermo Fisher Scientific) were coated with 1 ml per well of StemSpan Lymphoid Differentiation Coating Material. The stage 1 cells were collected and resuspended at 1 × 10^5^ cells per ml; 2 ml of cell suspension was added into each precoated well. Cells were passaged 1–2 times per week to maintain a cell density at 0.5–1 × 10^6^ cells per ml; fresh medium was added at every passage.

At stage 3, cells collected from the stage 2 were cultured in the StemSpan SFEM II Medium supplemented with StemSpan Lymphoid Progenitor Maturation Supplement, CD3/CD28/CD2 T Cell Activator and 20 ng ml^−1^ human recombinant IL-15 for 1 week. Cells were resuspended at 5 × 10^5^ cells per ml; 2 ml cell suspension was added into Non-Treated Falcon Polystyrene six-well Microplates (Thermo Fisher Scientific) precoated with 1 ml per well of StemSpan Lymphoid Differentiation Coating Material. Cells were passaged 2–3 times per week to maintain a cell density at 0.5–1 × 10^6^ cells per ml; fresh medium was added at every passage.

At stage 4, cells collected from the stage 3, now mature ^Allo/15^(CAR)-NKT cells or their derivatives, were expanded using various expansion approaches: (1) an αCD3/αCD28 expansion approach, (2) an αGC–PBMC expansion approach or (3) an aAPC expansion approach. The expansion stage lasted for 2 weeks. At stage 4, cells could be cultured in 150 mm cell culture dishes (Thermo Fisher Scientific) or G-Rex 6M Well Plates (Wilson Wolf). The expansion can happen in a feeder-free, serum-free CTS OpTmizer T Cell Expansion SFM (Thermo Fisher Scientific) or a homemade C10 medium. The resulting ^Allo/15^(CAR)-NKT or derivative cell products were aliquoted and cryopreserved in CryoStor Cell Cryopreservation Media CS10 using a Thermo Scientific CryoMed Controlled-Rate Freezer 7450 (Thermo Scientific) for future use, following the manufacturer’s instructions.

1. The αCD3/αCD28 antibody expansion approach used 150 mm cell culture dishes (Thermo Fisher Scientific), which were coated with 1 µg ml^−1^ (500 µl per well) of Ultra-LEAF Purified Anti-Human CD3 Antibody (Clone OKT3; BioLegend) for 2 h at room temperature or, alternatively, overnight at 4 °C. Mature ^Allo/15^(CAR)-NKT cells or their derivatives collected from the stage 3 culture were resuspended in the expansion medium supplemented with 10 ng ml^−1^ IL-7, 10 ng ml^−^^1^ IL-15 and 1 μg ml^−1^ Ultra-LEAF Purified Anti-Human CD28 antibody (Clone CD28.2; BioLegend) at 5 × 10^5^ cells per ml; 30 ml cell suspension was added into each plate. After 3 days of culture, cells were collected and resuspended in fresh expansion medium supplemented with 10 ng ml^−1^ IL-7 and IL-15, at 0.5–1 × 10^6^ cells per ml. Cells were passaged 2–3 times per week to maintain a cell density at 0.5–1 × 10^6^ cells per ml; fresh medium was added at every passage. As the cell population reaches a high number during the later stages, these cells can be transferred and cultured in G-Rex 6M Well Plates (Wilson Wolf).
2. The αGC–PBMC expansion approach. Healthy donor PBMCs were loaded with α-Galactosylceramide (αGC; Avanti Polar Lipids) at 5 μg ml^−1^ in C10 medium for 1 h following a previously established protocol^23^. The resulting αGC-loaded PBMCs (αGC–PBMCs) were then irradiated at 6,000 rads using a Rad Source RS-2000 X-Ray Irradiator (Rad Source Technologies). Mature ^Allo/15^CAR-NKT cells and derivatives collected from the stage 3 culture were mixed with the irradiated αGC–PBMCs at a 1:5 ratio, resuspended in expansion medium supplemented with 10 ng ml^−1^ IL-7 and IL-15 at 0.5–1 × 10^6^ cells per ml and seeded into the 150 mm cell culture dishes (Thermo Fisher Scientific) at 30 ml per plate. Cells were passaged 2–3 times per week to maintain a cell density at 0.5–1 × 10^6^ cells per ml; fresh medium was added at every passage. As the cell population reaches a high number during the later stages, these cells can be transferred and cultured in G-Rex 6M Well Plates (Wilson Wolf).
3. The aAPC expansion approach. aAPCs were irradiated at 10,000 rads using a Rad Source RS-2000 X-Ray Irradiator (Rad Source Technologies). Mature ^Allo/15^CAR-NKT cells and derivatives collected from the stage 3 culture were mixed with the irradiated aAPCs at a 1:1 ratio, resuspended in expansion medium supplemented with 10 ng ml^−1^ IL-7 and IL-15 at 0.5–1 × 10^6^ cells per ml, and seeded into the 150 mm cell culture dishes (Thermo Fisher Scientific) at 30 ml per plate. Cells were passaged 2–3 times per week to maintain a cell density at 0.5–1 × 10^6^ cells per ml; fresh medium was added at every passage. As the cell population reaches a high number, cells can be transferred to and cultured in G-Rex 6M Well Plates (Wilson Wolf).

### Generation of PBMC-derived conventional αβT, NKT and NK cells

Healthy donor PBMCs were used to generate the PBMC-derived conventional αβ T, NKT and NK cells (denoted as PBMC-Tc, PBMC-NKT and PBMC-NK cells, respectively).

To generate PBMC-Tc cells, PBMCs were stimulated with Dynabeads Human T-Activator CD3/CD28 (Thermo Fisher Scientific) according to the manufacturer’s instructions, followed by culturing in the C10 medium supplemented with 20 ng ml^−1^ IL-2 for 2–3 weeks.

To generate PBMC-NKT cells, PBMCs were sorted with magnetic-activated cell sorting (MACS) via Anti-iNKT Microbeads (Miltenyi Biotech) labeling to enrich NKT cells, following the manufacturer’s instructions. The enriched NKT cells were mixed with donor-matched irradiated αGC–PBMCs at a ratio of 1:1, followed by culturing in C10 medium supplemented with 10 ng ml^−1^ IL-7 and IL-15 for 2–3 weeks. If needed, the resulting cultured cells could be further purified using fluorescence-activated cell sorting (FACS) via human NKT TCR antibody (Clone 6B11; BD Biosciences) staining.

To generate PBMC-NK cells, PBMCs were sorted with FACS using a FACSAria III Sorter (BD Biosciences) via human CD56 antibody (Clone HCD56; BioLegend) labeling or with MACS using a Human NK Cell Isolation Kit (Miltenyi Biotech), following the manufacturers’ instructions.

### Generation of CAR-engineered conventional αβ T (CAR-T) cells

Non-treated tissue culture 24-well plates (Corning) were coated with Ultra-LEAF Purified Anti-Human CD3 Antibody (Clone OKT3; BioLegend) at 1 µg ml^−1^ (500 µl per well), at room temperature for 2 h or at 4 °C overnight. Healthy donor PBMCs were resuspended in the C10 medium supplemented with 1 µg ml^−1^ Ultra-LEAF Purified Anti-Human CD28 Antibody (Clone CD28.2, BioLegend) and 30 ng ml^−1^ IL-2, followed by seeding in the precoated plates at 1 × 10^6^ cells per ml (1 ml per well). On day 2, cells were transduced with lentiviruses for 24 h. The resulting CAR-T cells were expanded for about 2 weeks in C10 medium and cryopreserved for future use.

### Generation of PBMC-derived CAR-engineered NKT (^PBMC^CAR-NKT) cells

Healthy donor PBMCs were sorted with MACS via Anti-iNKT Microbeads (Miltenyi Biotech) labeling to enrich NKT cells, following the manufacturer’s instructions. The enriched NKT cells were mixed with donor-matched irradiated αGC–PBMCs at a ratio of 1:1, followed by culturing in C10 medium supplemented with 10 ng ml^−1^ IL-7 and IL-15. On day 3, NKT cells were transduced with Lenti/BCAR-IL15 viruses for 24 h. The resulting CAR-NKT cells were expanded for about 2 weeks in C10 medium supplemented with 10 ng ml^−1^ IL-7 and IL-15 and cryopreserved for future use.

### In vitro tumor cell killing assay

Tumor cells (1 × 10^4^ cells per well) were cocultured with therapeutic cells (at ratios indicated in figure legends) in Corning 96-well clear bottom black plates for 8–24 h, in C10 medium with or without the addition of αGC (100 ng ml^−1^). At the end of culture, live tumor cells were quantified by adding d-luciferin (150 μg ml^−1^; Caliper Life Science) to cell cultures and reading out luciferase activities using an Infinite M1000 microplate reader (Tecan). In some experiments, 10 μg ml^−1^ LEAF purified antihuman NKG2D (Clone 1D11, BioLegend), antihuman DNAM-1 antibody (Clone 11A8, BioLegend) or LEAF purified mouse lgG2bk isotype control antibody (Clone MG2B-57, BioLegend) was added to cocultures, to study the NK activating receptor-mediated tumor cell killing mechanism.

### In vitro assays using samples from patients with MM

Primary BM samples from patients with MM were collected and subsequently diluted in PBS and subjected to density gradient centrifugation using Ficoll-Paque (Thermo Fisher Scientific) to obtain mononuclear cells following the manufacturer’s instructions. The resulting cells were cryopreserved for future use.

In one assay, the primary samples from patients with MM were analyzed for tumor cell phenotype and the TME composition using flow cytometry. Tumor cells were sorted using a Human Tumor Cell Isolation Kit (Miltenyi Biotec) and/or identified as CD45^−^CD31^−^FAP (fibroblast activation protein)^−^ cells, T cells were identified as CD45^+^CD3^+^ cells, B cells were identified as CD45^+^CD19^+^ cells, NK cells were identified as CD45^+^CD56^+^ cells, monocytes and macrophages were identified as CD45^+^CD11b^+^CD14^+^ cells, granulocytes were identified as CD45^+^CD11b^+^CD15^+^ cells and granulocyte-like MDSCs were identified as CD45^+^CD15^+^HLA-DR^+^ cells. Surface expression of BCMA, CD1d and NK ligands on tumor or/and immune cells were also analyzed using flow cytometry.

In another assay, the primary samples from patients with MM were used to study tumor cell killing by ^Allo15^BCAR-NKT cells. Tumor cells were presorted using a Human Tumor Cell Isolation Kit (Miltenyi Biotec), followed by coculturing with various therapeutic cells (therapeutic cell:tumor cell ratio 1:1) in C10 medium in Corning 96-well Round Bottom Cell Culture plates for 24 h. At the end of culture, cells were collected and live MM tumor cells (identified as CD45^−^CD3^−^6B11^−^) were analyzed using flow cytometry.

In another assay, the primary samples from patients with MM were used to study the TME targeting by ^Allo15^BCAR-NKT cells. Patient samples were directly cocultured with ^Allo15^BCAR-NKT cells (therapeutic cell:tumor cell ratio 1:1) in C10 medium in Corning 96-well Round Bottom Cell Culture plates for 24 h. At the end of culture, cells were collected and the TME targeting of ^Allo15^BCAR-NKT cells was assessed using flow cytometry by quantifying live human monocytes and macrophages (identified as 6B11^−^CD45^hi^CD14^+^CD11b^+^), MDSCs (identified as 6B11^−^CD45^med^CD15^+^CD11b^+^HLA-DR^+^), CD4 T cells (identified as 6B11^−^CD45^hi^CD3^+^CD4^+^), CD8 T cells (identified as 6B11^−^CD45^hi^CD3^+^CD8^+^), B cells (identified as 6B11^−^CD45^hi^CD3^−^CD19^+^), granulocytes (identified as 6B11^−^CD45^med^CD15^+^CD11b^+^HLA-DR^−^) and NK cells (identified as 6B11^−^CD45^hi^CD3^−^CD56^+^).

### In vitro MLR assay for studying GvH response

PBMCs of multiple random healthy donors were irradiated at 2,500 rads and used as stimulators to study the GvH response of ^Allo15^BCAR-NKT cells as responders. BCAR-T cells were included as a responder control. Stimulators (5 × 10^5^ cells per well) and responders (2 × 10^4^ cells per well) were cocultured in 96-well round-bottom plates in C10 medium for 4 days; the cell culture supernatants were then collected to measure IFNγ production using enzyme-linked immunosorbent assay (ELISA).

### In vitro MLR assay for studying T cell-mediated allorejection

PBMCs of multiple healthy donors were used as responders to study the T cell-mediated allorejection of ^Allo15^BCAR-NKT cells as stimulators (irradiated at 2,500 rads). PBMC-derived BCAR-T cells were included as a stimulator control. Stimulators (5 × 10^5^ cells per well) and responders (2 × 10^4^ cells per well) were cocultured in 96-well round-bottom plates in C10 medium for 4 days; the cell culture supernatants were then collected to measure IFNγ production using ELISA.

### In vitro MLR assay for studying NK cell-mediated allorejection

PBMC-derived NK cells obtained from multiple healthy donors were employed to investigate the NK cell-mediated allorejection of ^Allo15^BCAR-NKT cells. Conventional BCAR-T cells were included as an allogeneic subject control. PBMC-NK cells (2 × 10^4^ cells per well) and the corresponding allogeneic subject cells (2 × 10^4^ cells per well) were cocultured in 96-well round-bottom plates with C10 medium for 24 h. Subsequently, the cell cultures were collected to quantify live cells via flow cytometry.

### In vivo BLI

Bioluminescence live animal imaging (BLI) was performed using a Spectral Advanced Molecular Imaging (AMI) HTX imaging system (Spectral instrument Imaging). Live animal images were acquired 5 min after intraperitoneal (i.p.) injection of d-luciferin for total body bioluminescence. To monitor the signal of tumor cells, 1 mg per mouse of d-luciferin was injected. To monitor the signal of therapeutic cells, 3 mg per mouse of d-luciferin was injected. Imaging data were analyzed using AURA imaging software (Spectral Instrument Imaging, v.3.2.0).

### In vivo antitumor efficacy study of ^Allo/15^BCAR-NKT cells: human MM xenograft NSG mouse model

Experimental design is shown in Fig. 3a. Briefly, on Day 0, NSG mice received intravenously (i.v.) inoculation of MM-FG human MM cells (1 × 10^6^ cells per mouse). On day 10, the experimental mice received i.v. injection of vehicle (100 μl PBS per mouse), ^Allo/15^BCAR-NKT cells (10 × 10^6^ CAR^+^ cells in 100 μl PBS per mouse), or control BCAR-T cells (10 × 10^6^ CAR^+^ cells in 100 μl PBS per mouse). Over the experiment, mice were monitored for survival and their tumor loads were measured using BLI.

### In vivo antitumor efficacy study of ^Allo15^CAR19-NKT cells: human B cell leukemia xenograft NSG mouse model

Experimental design is shown in Supplementary Fig. 15h. Briefly, on day 0, NSG mice received i.v. inoculation of Nalm6-FG human B cell leukemia cells (1 × 10^6^ cells per mouse). On day 4, the experimental mice received an i.v. injection of vehicle (100 μl PBS per mouse), ^Allo15^CAR19-NKT cells (5 × 10^6^ CAR^+^ cells in 100 μl PBS per mouse) or control CAR19-T cells (10 × 10^6^ CAR^+^ cells in 100 μl PBS per mouse). Over the experiment, mice were monitored for survival and their tumor loads were measured using BLI.

### In vivo PK–PD study of ^Allo/15^BCAR-NKT/FG cells: human MM xenograft NSG mouse model

Experimental design is shown in Fig. 3f. Briefly, on day 0, NSG mice received i.v. inoculation of MM. 1S cells (1 × 10^6^ cells per mouse; denoted as MM). On day 10, the experimental mice received i.v. injection of ^Allo/15^BCAR-NKT/FG cells (10 × 10^6^ CAR^+^ cells in 100 μl of PBS per mouse) or control BCAR-T/FG cells (10 × 10^6^ CAR^+^ cells in 100 μl PBS per mouse). Over the experiment, mice were monitored for survival and their therapeutic cells were measured using BLI. Note in this study, therapeutic immune cells, but not the tumor cells, were labeled with FG.

### In vivo CRS study

Experimental design is shown in Fig. 5p. Briefly, on day 0, NSG mice received i.v. inoculation of MM-FG cells (5 × 10^6^ cells per mouse). On day 10, the experimental mice received i.v. injection of vehicle (100 μl PBS per mouse), ^Allo15^BCAR-NKT cells (10 × 10^6^ CAR^+^ cells in 100 μl PBS per mouse) or control BCAR-T cells (10 × 10^6^ CAR^+^ cells in 100 μl PBS per mouse). On days 11 and 13, blood samples were collected from the experimental mice, and their serum IL-6 and SAA-3 were measured using ELISA. A Mouse SAA-3 ELISA Kit (Millipore Sigma) was used to measure SAA-3, following the manufacturer’s instructions.

### Single-cell TCR sequencing

PBMC-Tc, PBMC-NKT, ^Allo^NKT and ^Allo/15^BCAR-NKT cells were sorted using a FACSAria III Sorter (BD Biosciences). Sorted cells were immediately delivered to the UCLA Technology Center for Genomics and Bioinformatics (TCGB) Core to perform single-cell TCR sequencing using a 10X Genomics Chromium Controller Single Cell Sequencing System (10X Genomics), following the manufacturer’s instructions and the TCGB Core’s standard protocols. Libraries were constructed using an Illumina TruSeq RNA Sample Prep Kit (Illumina) and sequenced with 150 bp paired-end reads (5,000 reads per cell) on an Illumina NovaSeq 6000 sequencer. The reads were mapped to the human TCR reference genome (hg38) using Cell Ranger VDJ (10X Genomics). The frequencies of the α or β chain recombination were plotted.

### Bulk RNA-seq

A total of 24 cell samples were analyzed, including three ^Allo^NKT, three ^Allo^BCAR-NKT, three ^Allo15^BCAR-NKT, two ^Allo^CAR19-NKT, three PBMC-NKT, eight PBMC-Tc and two PBMC-NK cell samples; the numbers indicate different donors. Note that ^Allo/15^(CAR)-NKT cells displayed a CD4^−^CD8^+/−^ phenotype, therefore the CD4^−^ populations of PBMC-NKT and PBMC-Tc cells were used in this study.

Cell samples were sorted using a FACSAria III Sorter (BD Biosciences). Total RNAs were isolated from each cell sample using a miRNeasy Mini Kit (Qiagen) and delivered to the UCLA TCGB Core to perform bulk RNA-seq using an Illumina HiSeq3000, following the manufacturer’s instructions and the TCGB Core’s standard protocols. Complementary DNAs were synthesized using an iScript cDNA Synthesis Kit (BioRad). Libraries were constructed using an Illumina TruSeq Stranded Total RNA Sample Prep kit and sequenced with 50 bp single-end reads (targeting 20 × 10^6^ reads per sample) on an Illumina HiSeq3000. The raw sequencing data underwent quality control and adapter trimming using fastp (v.0.23.2), and trimmed reads were mapped to the human genome (hg38) using with STAR v.2.7.9a with default parameters. The read counts for each gene were obtained using featureCounts from Subread package (v.2.0.3). Sequencing depth normalized counts (in counts per million) were obtained using edgeR (v.3.38.4), and the log transformation with psuedocount 1 was used for principal component analysis.

### scRNA-seq

In one study, scRNA-seq was used to investigate the lineage commitment of ^Allo/15^BCAR-NKT cells during their in vitro differentiation from gene-engineered HSPCs. Cord-blood CD34^+^ HSPCs of the same donor were transduced with either a Lenti/iNKT-BCAR or Lenti/iNKT-BCAR-IL15 lentivector, followed by differentiating into ^Allo^BCAR-NKT or ^Allo15^BCAR-NKT cell product, respectively, in the Ex Vivo HSPC-Derived CAR-NKT Cell Culture. At days 7, 14, 21, 28 and 42, cell cultures were collected for scRNA-seq.

In another study, scRNA-seq was used to examine the in vivo gene profiles of therapeutic cells, tumor cells and mouse immune cells in an MM-FG human MM xenograft NSG mouse model as shown in Fig. 4a. The three types of therapeutic cell (^Allo15^BCAR-NKT, ^Allo^BCAR-NKT, and BCAR-T cells) were collected at three time points (days 0, 28 and 35). Day 0 samples were collected before infusion, day 28 samples were collected from the BM tumor sites of the experimental mice and day 35 samples were generated by stimulating day 28 samples in vitro with BCMA-expressing aAPCs for 1 week. The tumor cells (MM-FG) and mouse immune cells (mouse CD45^+^ cells) were collected on day 28. Cells isolated from ten mice of each experimental group were combined for scRNA-seq.

Freshly collected samples were immediately delivered to the UCLA TCGB Core for library construction and scRNA-seq. Cells were quantified using a Cell Countess II automated cell counter (Invitrogen/Thermo Fisher Scientific). A total of 10,000 cells from each experimental group were loaded on the Chromium platform (10X Genomics), and libraries were constructed using the Chromium Next GEM Single Cell 3′ Kit v3.1 and the Chromium Next GEM Chip G Single Cell Kit (10X Genomics), according to the manufacturer’s instructions. Library quality was assessed using the D1000 ScreenTape on a 4200 TapeStation System (Agilent Technologies). Libraries were sequenced on an Illumina NovaSeq using the NovaSeq S4 Reagent Kit (100 cycles; Illumina).

For cell clustering and annotation, the merged digital expression matrix generated by Cell Ranger was analyzed using an R package Seurat (v.4.0.0) following the guidelines. Briefly, after filtering the low-quality cells, the expression matrix was normalized using NormalizeData function, followed by selecting variable features across datasets using FindVariableFeatures and SelectIntegrationFeatures functions. To correct the batch effect, FindIntegrrationAnchors and IntegrateData functions were used based on the selected feature genes. The corrected dataset was subjected to standard Seurat workflow for dimension reduction and clustering. In this study, clusters of therapeutic cells were manually merged and annotated based on gene signatures reported from Human Protein Atlas (proteinatlas.org) and previous studies^38–45^, and clusters of mouse immune cells were merged and annotated based on the immune lineage markers. AddModuleScore was used to calculate module scores of each list of gene signatures, and FeaturePlot function was used to visualize the expression of each signature in the UMAP plots.

For gene set enrichment analysis (GSEA), clusterProfiler packages^87,88^ were used to calculate the enrichment scores of each cluster in the signature gene list.

For RNA-velocity analysis, the .LOOM files containing spliced and unspliced expression matrices were generated for each sample. The further analysis was conducted using the Velocyto.R-package (v.0.6)^89^. After loading .LOOM file information through the ReadVelocity function, databases were merged and the RunVelocity function was executed to obtain the velocity vectors. Finally, the velocities were projected into a lower-dimensional embedding using the velocity_graph function and visualized on the UMAP embedding in each intended cell cluster using the show.velocity.on.embedding.cor function. All velocyto functions were used with default parameters.

### Methylation sequencing

Genomic DNA was isolated from experimental samples using a QIAGEN DNeasy Blood & Tissue kit, then sonicated using a Covaris M220 Focused-ultrasonicator, following the manufacturers’ instructions. DNA fragments of around 250 bp were enriched using the Ampure XP beads (Beckman-Coulter), then subjected to DNA library preparation using an NEBNext Ultra II DNA library prep Kit (cat. no. E7645) following the manufacturers’ instructions. The DNA libraries were then subjected to sequencing on Illumina NovaSeq sequencer with 2 × 150 bp configuration (Azenta). The raw bisulfite sequencing reads were trimmed by cutadapt to remove sequencing adapters. The trimmed reads were then aligned to hg19 reference genome by Bismark^90^. Then duplicated reads from PCR amplification were identified and removed by Bismark. The deduplicated reads were then sorted and indexed using Samtools. After that, the methylated and unmethylated cytosines were counted at every CpG site by Bismark. The methylation at a gene promoter region was quantified as the beta value, that is, the ratio between the number of methylated cytosines and the total number of cytosines mapped to the region. The promoter region of a gene was identified by GeneHancer with the highest confidence score. The beta values were then used for heatmap visualization.

### Histology

Tissues (that is, heart, liver and lung) were collected from experimental mice, fixed in 10% Neutral Buffered Formalin for up to 36 h and embedded in paraffin for sectioning (5 μm thickness). Tissue sections were prepared and stained with hematoxylin and eosin by the UCLA Translational Pathology Core Laboratory, following the Core’s standard protocols. Stained sections were imaged using an Olympus BX51 upright microscope equipped with an Optronics Macrofire CCD camera (AU Optronics). The images were analyzed using Optronics PictureFrame software (AU Optronics).

### Western blot

Western blot was used to analyze the IFNγ signaling events in ^Allo/15^BCAR-NKT cells. Conventional BCAR-T cells and quiescent T cells prepared from the same healthy donor PBMCs were included as controls. Quiescent T cells were sorted with MACS from PBMCs using mixed antihuman CD4 and CD8 magnetic beads (Miltenyi Biotec) to avoid the potential CD3 stimulation, following the manufacturer’s instructions.

Cells were stimulated with IFNγ (10 ng ml^−1^) for 15 min (for blotting p-STAT1/STAT1), 18 h (for blotting IRF-1) or 48 h (for blotting CIITA/SP1). Total proteins were extracted using a RIPA lysis buffer (Thermo Fisher Scientific) containing 20 mM HEPES (pH 7.6), 150 mM NaCl, 1 mM EDTA, 1% Tritonx-100 and protease–phosphatase inhibitor cocktail (Cell Signaling Technology). Protein concentration was measured using a Bicinchoninic Acid Assay Kit (Thermo Fisher Scientific). Equal amounts of total protein were resolved on a 4–15% Mini-PROTEAN TGX Precast Protein Gel (BioRad) and then transferred to a polyvinylidene difluoride membrane by electrophoresis. The following antibodies were used to blot for the proteins of interest: antihuman CIITA (Invitrogen, cat. no. PA5-21031, 1:1,000 dilution), antihuman p-STAT1(Y701) (Clone 58D6, Cell Signaling Technology, CST, cat. no. 9167, 1:1,000 dilution), antihuman STAT1 (Clone D1K9Y, CST, cat. no. 14994, 1:1,000 dilution), antihuman IRF-1 (Clone D5E4, CST, cat. no. 8478, 1:1,000 dilution), antihuman SP1 (Clone D4C3, CST, cat. no. 9389, 1:1,000 dilution) and secondary antirabbit IgG (CST, cat. no. 7074, 1:1,000 dilution). β-Actin (Clone D6A8, CST, cat. no. 8457, 1:3,000 dilution) and glyceraldehyde 3-phosphate dehydrogenase (Clone D16H11, CST, cat. no. 5174, 1:3,000 dilution) were used as internal controls. Signals were visualized using a ChemiDoc Imaging Systems (Bio-Rad). The data were analyzed using ImageJ (v.1.53s).

### Generation and analysis of ^Allo15^BCAR-NKT cells overexpressing a constitutively active STAT1 (denoted as ^Allo15^BCAR-NKT/STAT1C cells)

^Allo15^BCAR-NKT/STAT1C cells were generated by further engineering the ^Allo15^BCAR-NKT cells to overexpress a constitutively active human STAT1 (STAT1C)^85^. The Lenti/STAT1C-EGFP vector or a mock control Lenti/Fluc-EGFP vector was added into the ^Allo15^BCAR-NKT cell culture during stage 4 (on day 3 of stage 4), followed by the continuation and completion of stage 4 culture. The resulting ^Allo15^BCAR-NKT/STAT1C cells, as well as their mock control ^Allo15^BCAR-NKT/FG cells, were then analyzed using flow cytometry. The overexpression of transgenic STAT1C was measured by EGFP coexpression in ^Allo15^BCAR-NKT/STAT1C cells. The rescue effect of STAT1C overexpression in EGFP^+ Allo15^BCAR-NKT/STAT1C cells was assessed by comparing these cells with the control EGFP^+ Allo15^BCAR-NKT/FG cells for cell surface expression of HLA-I/II molecules and NK ligands.

### Statistics

Graphpad Prism v.8 software (Graphpad) was used for statistical data analysis. Student’s two-tailed *t*-test was used for pairwise comparisons. Ordinary one-way analysis of variance (ANOVA) followed by Tukey’s or Dunnett’s multiple comparisons test was used for multiple comparisons. A log rank (Mantel–Cox) test adjusted for multiple comparisons was used for Meier survival curves analysis. Data are presented as the mean ± s.e.m., unless otherwise indicated. In all figures and figure legends, *n* represents the number of samples or animals used in the indicated experiments. A *P* value of less than 0.05 was considered significant and NS denotes not significant.

### Reporting summary

Further information on research design is available in the Nature Portfolio Reporting Summary linked to this article.

## Online content

Any methods, additional references, Nature Portfolio reporting summaries, source data, extended data, supplementary information, acknowledgements, peer review information; details of author contributions and competing interests; and statements of data and code availability are available at 10.1038/s41587-024-02226-y.

## Supplementary information

Supplementary information Supplementary Figs. 1–19.

Reporting Summary

Supplementary Data 1 Source data for Supplementary figures.

## Source data

Source Data Fig. 1 Statistical source data.

Source Data Fig. 2 Statistical source data.

Source Data Fig. 3 Statistical source data.

Source Data Fig. 4 Statistical source data.

Source Data Fig. 5 Statistical source data.

Source Data Fig. 6 Statistical source data and unprocessed western blots.

Source Data Fig. 7 Statistical source data.

## Data Availability

All data associated with this study are present in the paper or Supplementary Information. The genomics data generated during this study are available from the public repository Gene Expression Omnibus Database: GSE253982 (scTCR-seq, related to Fig. 1h), GSE245375 (bulk RNA-seq, related to Fig. 1i), GSE241996 (scRNA-seq for the in vitro study related to Fig. 1g), GSE241998 (scRNA-seq for the in vivo study related to Fig. 4), GSE241999 (scRNA-seq for the in vivo study related to Fig. 5a–c), GSE241997 (scRNA-seq for the in vivo study related to Fig. 5h–n) and GSE239648 (Methyl-seq, related to Fig. 6n). Additional information and materials will be made available upon reasonable request. Source data are provided with this paper.
