# Supplementary material for: Generation of allogeneic CAR-NKT cells from hematopoietic stem and progenitor cells using a clinically guided culture method
Source: Nat Biotechnol. 2024 May 14;43(3):329–44. doi: 10.1038/s41587-024-02226-y (PMC11919731; doi:10.1038/s41587-024-02226-y)
Supplement: Supplementary file 1 — Supplementary Figs. 1–19. [file 41587_2024_2226_MOESM1_ESM.pdf]

nature biotechnology

Article https://doi.org/10.1038/s41587-024-02226-y

Generation of allogeneic CAR-NKT cells from hematopoietic stem and progenitor cells using a clinically guided culture method

In the format provided by the authors and unedited

Table of Contents

Supplementary Fig. 1. Generation of ^{Allo15}CAR-NKT cells using various CARs.................... 2

Supplementary Fig. 2. Generation and characterization of ^{Allo}NKT cells and their CAR-armed derivatives (^{Allo}CAR-NKT cells), related to Fig. 1.. 4

Supplementary Fig. 3. Examination of the CD8α/α and CD8α/β forms in CD8^{+} ^{Allo}(CAR)-NKT cells, related to Fig. 1.. 5

Supplementary Fig. 4. Generation of PBMC-derived immune cells utilized in the study, related to Fig. 1j, 1k. 6

Supplementary Fig. 5. Phenotype and functionality of $^{Allo/15}$BCAR-NKT cells, related to Fig. 1.. 7

Supplementary Fig. 6. FACS detection of NKR targets on primary MM patient-derived tumor cells, related to Fig. 2b-2d. 9

Supplementary Fig. 7. In vitro antitumor efficacy and mechanism of action (MOA) study of $^{Allo/15}$BCAR-NKT cells, related to Fig. 2. 10

Supplementary Fig. 8. In vivo PK/PD study of $^{Allo/15}$BCAR-NKT/FG cells, related to Fig. 3.12

Supplementary Fig. 9. In vivo gene profiling of $^{Allo/15}$BCAR-NKT, related to Fig. 4............. 13

Supplementary Fig. 10. Antitumour capacity of Day 35 re-stimulation samples, related to Fig. 4. 14

Supplementary Fig. 11. BCMA expression on MM-FG tumor cells in the indicated samples, related to Fig. 5a-5c. NT, sample collected from tumor-bearing NSG mice receiving no therapeutic cell treatment.. 15

Supplementary Fig. 12. Studying the toxicity of ^{Allo15}BCAR-NKT cells against HSCs, related to Fig. 5. 16

Supplementary Fig. 13. Cell cluster definition using lineage markers, related to Fig. 5j. . 18

Supplementary Fig. 14. Studying the GvH response and allorejection of ^{Allo}NKT cells in comparison with PBMC-derived conventional αβ T and NKT cells, related to Fig. 6a-6h. 19

Supplementary Fig. 15. Characterization of ^{Allo15}CAR19-NKT cells....................................... 20

Supplementary Fig. 16. Solid tumor cell killing by ^{Allo15}CAR-NKT cells.............................. 22

Supplementary Fig. 17. Incorporation of an sr39TK suicide switch in allogeneic NKT cells... 23

Supplementary Fig. 18. Cord blood donor information. 24

Supplementary Fig. 19. CD1d expression on various human tissue cells and cancer types. 25

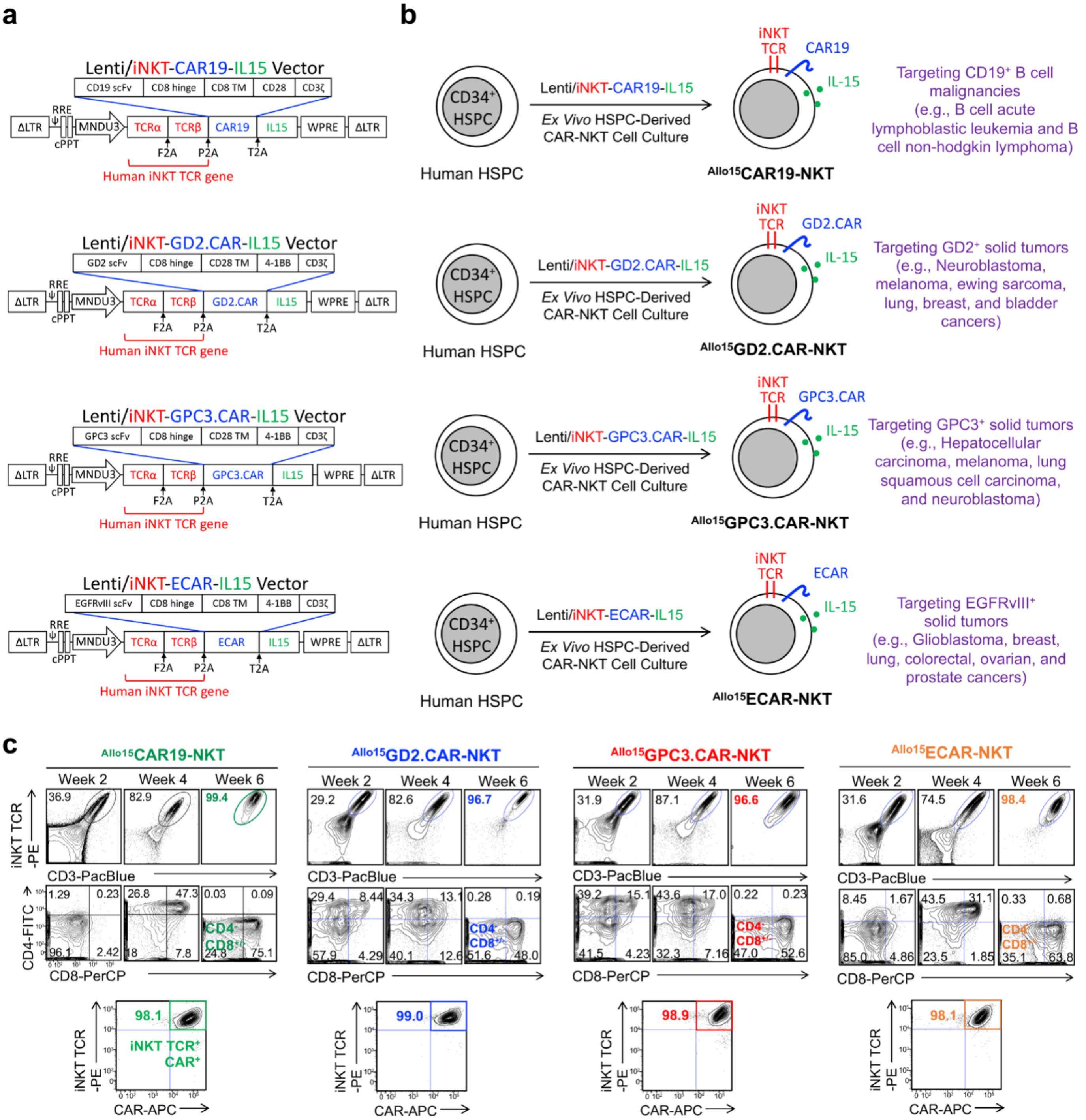

Supplementary Fig. 1. Generation of ^{Allo15}CAR-NKT cells using various CARs.
a. Schematics showing the design of four lentivectors, including Lenti/iNKT-CAR19-IL15, Lenti/iNKT-GD2.CAR-IL15, Lenti/iNKT-GPC3.CAR19, and Lenti/iNKT-ECAR-IL15. CAR19, CD19-targeting CAR; GD2.CAR, disialoganglioside GD2-targeting CAR; GPC3.CAR, glypican 3-targeting CAR; ECAR, epidermal growth factor receptor variant III (EGFRvIII)-targeting CAR.

b. Schematics showing the generation of ^{Allo15}CAR19-NKT, ^{Allo15}GD2.CAR-NKT, ^{Allo15}GPC3.CAR-NKT, and ^{Allo15}ECAR-NKT cells.
c. FACS monitoring of the generation of four ^{Allo15}CAR-NKT cells, and FACS detection of their iNKT TCR and CAR expressions.
Representative of > 3 experiments.

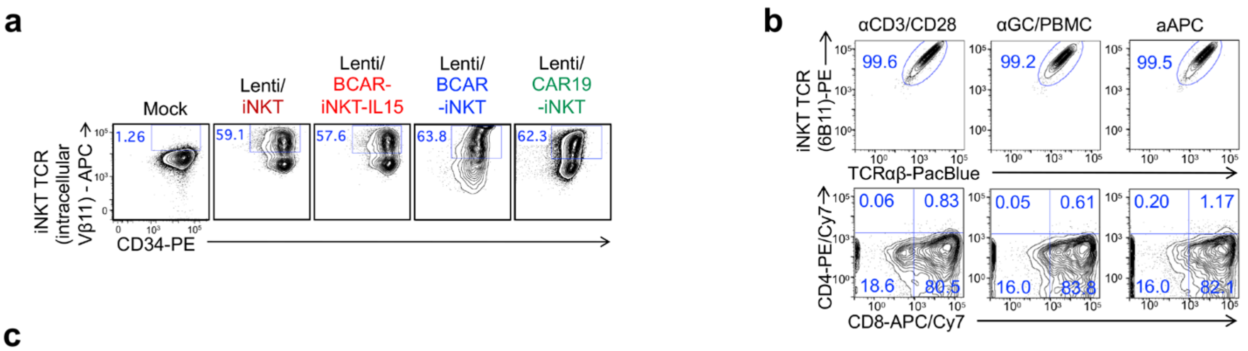

c

Estimated input and output cell numbers for the ^{Allo}(CAR)-NKT cell products at the research scale							
**Stage 1: HSC expansion** (~300-fold expansion during a 14-day culture)		**Stage 2: NKT differentiation** (~10-fold expansion during a 7-day culture)		**Stage 3: NKT deep differentiation** (~15-fold expansion during a 7-day culture)		**Stage 4: NKT expansion** (~150-fold expansion during a 14-day culture)	
Input cell number	Output cell number	Input cell number	Output cell number	Input cell number	Output cell number	Input cell number	Output cell number
1 x 10^4	~3 x 10^6	2 x 10^5	~2 x 10^6	2 x 10^6	~3 x 10^7	3 x 10^7	~4.5 x 10^9

The remaining ~2.8 x 10^6 cells were cryopreserved for future cultures

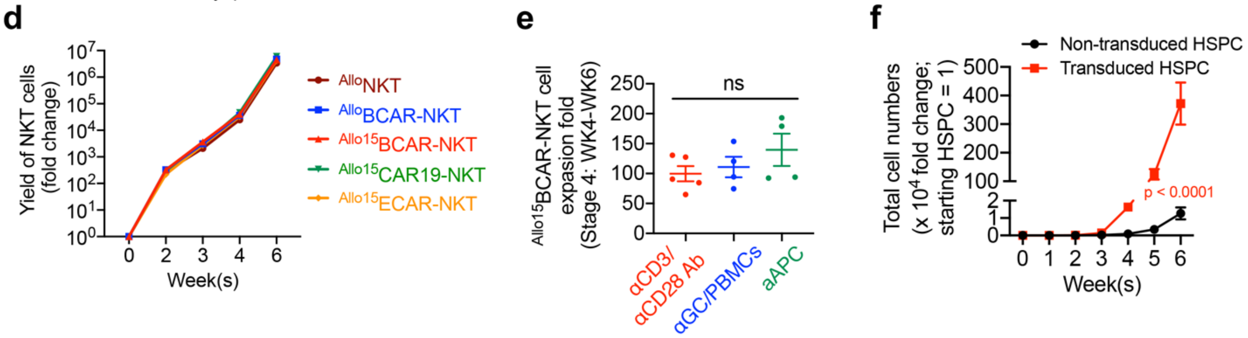

Supplementary Fig. 2. Generation and characterization of ^{Allo}NKT cells and their CAR-armed derivatives (^{Allo}CAR-NKT cells), related to Fig. 1.

a. FACS detection of intracellular iNKT TCR expression in CD34$^+$ CB HSPCs. At Stage 0, the indicated lentivector was added to HSPC cultures. Three days later, the transduced HSPCs were collected and analyzed for intracellular expression of iNKT TCR (identified as Vβ11$^+$) using flow cytometry.

b. FACS monitoring of ^{Allo}NKT cells cultured under the indicated expansion conditions. FACS data collected at day 7 post expansion are presented. αGC/PBMC, αGC-loaded PBMCs.

c. Table showing the actual input and output cell numbers of the ^{Allo}(CAR)-NKT cell products at the research scale.

d. Time course expansion of 5 ^{Allo}(CAR)-NKT cell products during the 6-week culture. Note that no significant differences were observed between cell products with/out IL-15 engineering (e.g., ^{Allo}BCAR-NKT vs. ^{Allo15}BCAR-NKT), or between IL-15-engineered ^{Allo}CAR-NKT cells expressing different CARs (e.g, ^{Allo15}BCAR-NKT vs. ^{Allo15}CAR19-NKT vs. ^{Allo15}ECAR-NKT).

e. Comparison of ^{Allo15}BCAR-NKT cell expansion fold using the three expansion methods (αCD3/αCD28 Ab, n = 5; αGC/PBMCs and aAPC, n = 4).

f. Growth curve of HSPCs either non-transduced or transduced with Lenti/iNKT in the culture (n = 5).

Representative of > 5 experiments. Data are presented as the mean ± SEM and were analyzed by two-tailed Student's *t* test (**f**) or one-way ANOVA (**e**). ns, not significant.

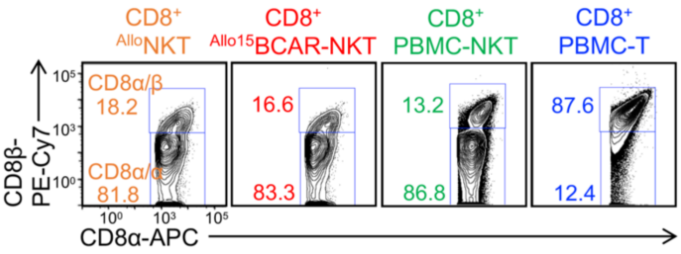

Supplementary Fig. 3. Examination of the CD8α/α and CD8α/β forms in CD8+ Allo(CAR)-NKT cells, related to Fig. 1.

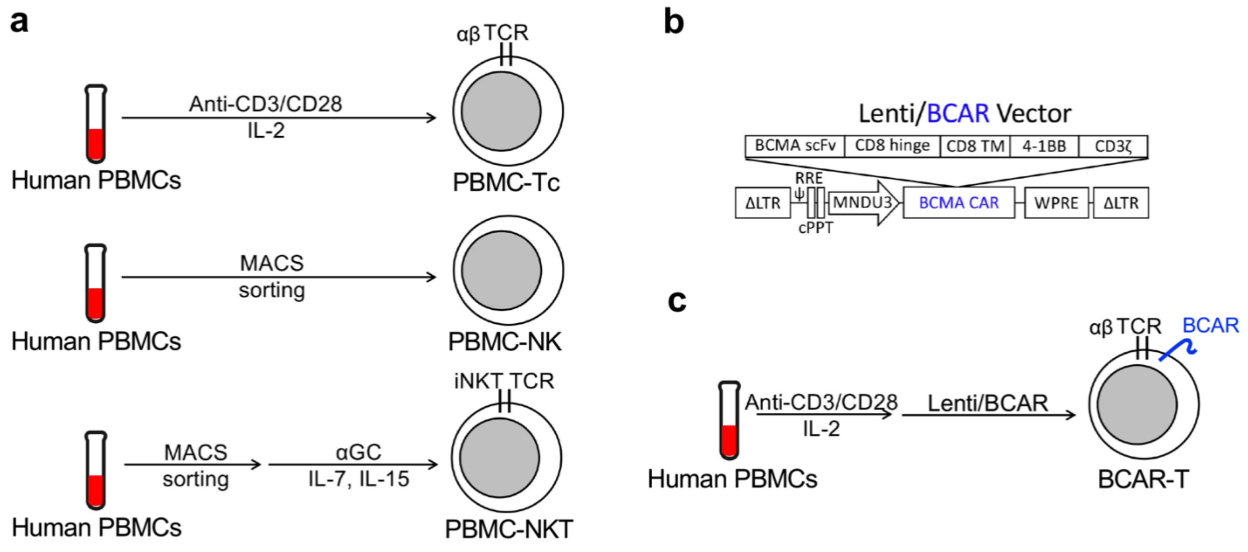

Supplementary Fig. 4. Generation of PBMC-derived immune cells utilized in the study, related to Fig. 1j, 1k.

a. Diagram showing the generation of PBMC-derived conventional αβ T (PBMC-Tc), NK (PBMC-NK), and NKT (PBMC-NKT) cells. αGC, α-galactosylceramide.

b. Schematics showing the design of indicated lentivectors. ΔLTR, long-terminal repeat; RRE, rev-responsive element; cPPT, central polypurine tract; MNDU3, LTR promoter for U3 region of myeloproliferative sarcoma retrovirus MND; WPRE, woodchuck hepatitis virus posttranscriptional regulatory element; scFv, single-chain variable fragment; CD8 TM, CD8 transmembrane domain; CD3ζ, CD3 zeta chain; BCMA, B cell maturation antigen; BCAR, BCMA-targeting CAR.

c. Diagram showing the generation of CAR-engineered conventional αβ T (CAR-T) cells.

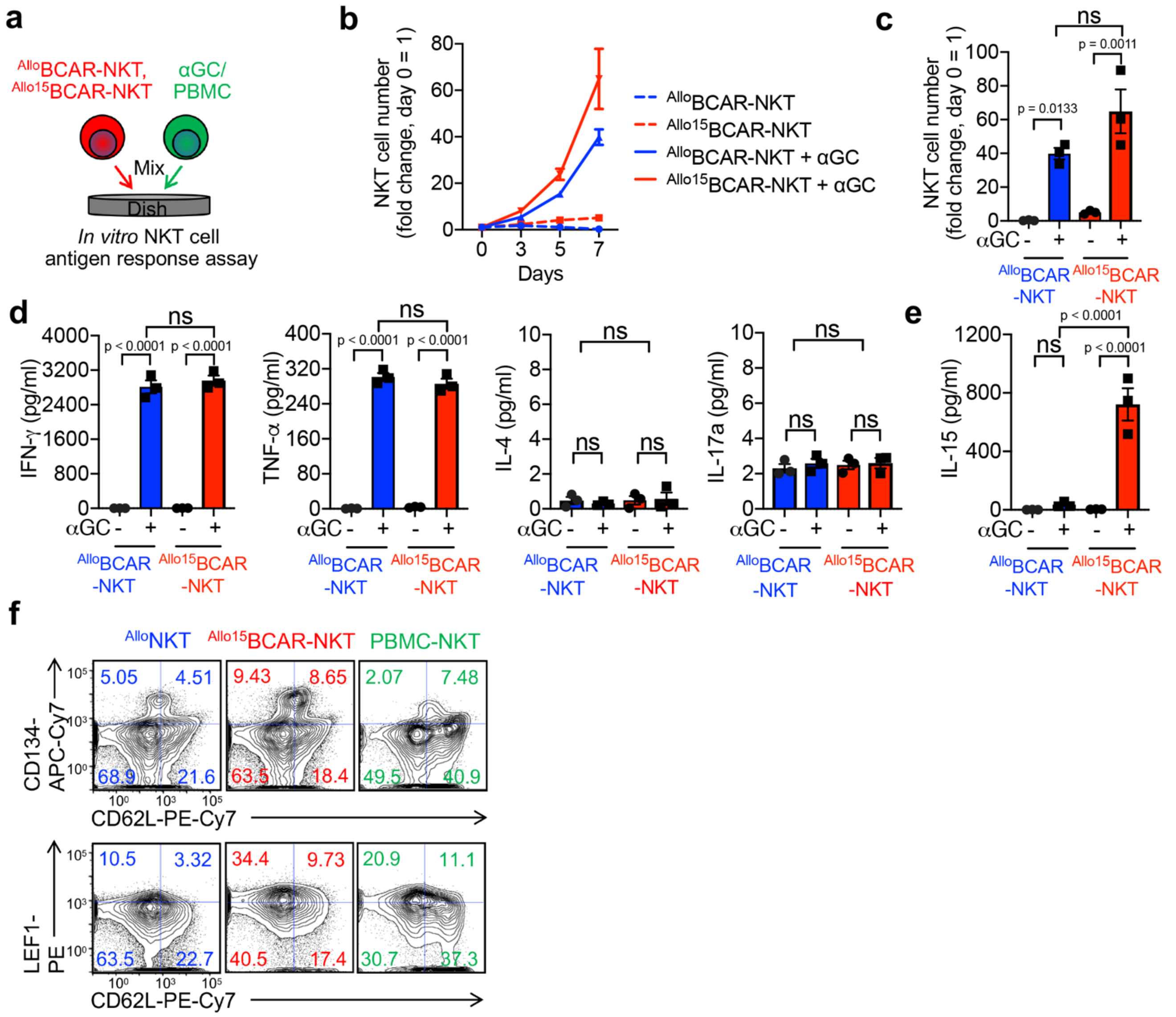

Supplementary Fig. 5. Phenotype and functionality of $^{Allo/15}$BCAR-NKT cells, related to Fig. 1.

a-d. Antigen responses of $^{Allo/15}$BCAR-NKT cells. $^{Allo/15}$BCAR-NKT cells were stimulated with/out αGC/PBMC for one week. **a.** Experimental design. **b.** Growth curve of $^{Allo/15}$BCAR-NKT cells (n = 3). **c.** Quantification of (**b**) on day 7. **d.** ELISA analyses of effector cytokine (IFN-γ, IL-2, IL-4, and IL-17a) production on day 7 (n = 3).

e. ELISA analyses of IL-15 production by $^{Allo/15}$BCAR-NKT cells cultured in the presence or absence of αGC stimulation for 48 hours (n = 3). Note the successful incorporation and expression of *IL-15* transgene in ^{Allo15}BCAR-NKT cells. Also note that IL-15 transgene is controlled by a constitutive promoter and therefore should be constitutively expressed; the seemly no production of IL-15 from ^{Allo15}BCAR-NKT cells without αGC stimulation likely attributes to a combination of low cell number and short half-life time of IL-15 in supernatants, resulting in a measurement below the ELISA detection level.

f. FACS detection of central memory markers in ^{Allo}(CAR)-NKT cells. Notably, IL15 engineering increased the central memory features.

Representative of three experiments. Data are presented as the mean ± SEM and were analyzed by one-way ANOVA (**c, d, e**).

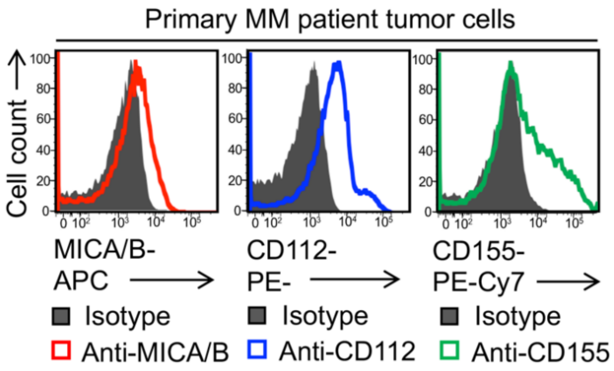

Supplementary Fig. 6. FACS detection of NKR targets on primary MM patient-derived tumor cells, related to Fig. 2b-2d.

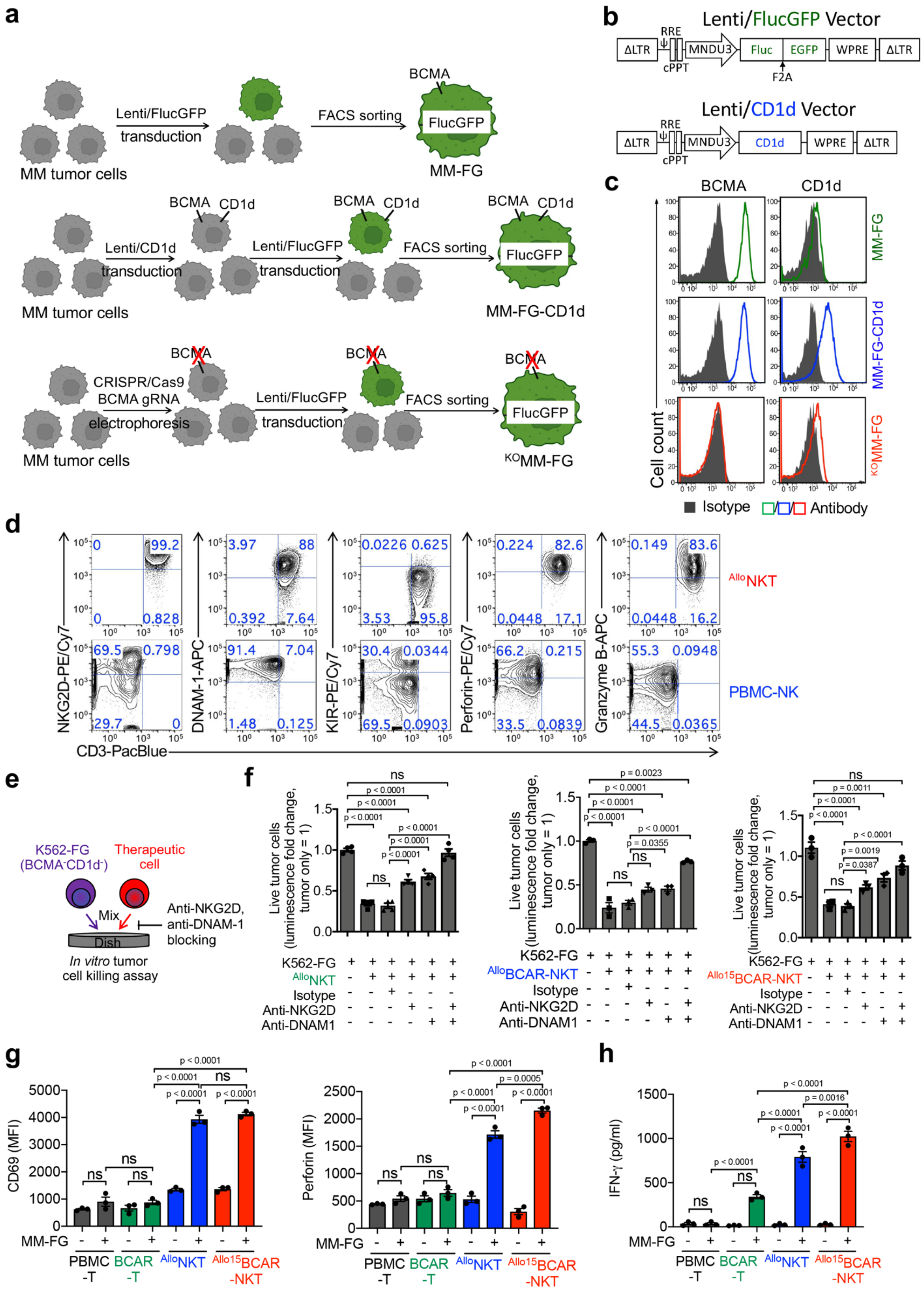
a
Lenti/FlucGFP transduction
FACS sorting
BCMA
FlucGFP
MM tumor cells
MM-FG
CD1d
Lenti/CD1d transduction
MM-FG-CD1d
CRISPR/Cas9 BCMA gRNA electrophoresis
KOMM-FG
b
Lenti/FlucGFP Vector
ΔLTR
RRE
ψ
cPPT
MNDU3
Fluc
EGFP
F2A
WPRE
Lenti/CD1d Vector
c
Cell count
MM-FG
MM-FG-CD1d
Isotype
Antibody
d
NKG2D-PE/Cy7
DNAM-1-APC
KIR-PE/Cy7
Perforin-PE/Cy7
Granzyme B-APC
CD3-PacBlue
AlloNKT
PBMC-NK
e
K562-FG (BCMA-CD1d-)
Therapeutic cell
Mix
Dish
Anti-NKG2D, anti-DNAM-1 blocking
In vitro tumor cell killing assay
f
Live tumor cells (luminescence fold change, tumor only = 1)
K562-FG
AlloNKT
AlloBCAR-NKT
Allo15BCAR-NKT
Isotype
Anti-NKG2D
Anti-DNAM1
ns
p < 0.0001
p = 0.0023
p = 0.0355
p = 0.0011
p = 0.0019
p = 0.0387
g
CD69 (MFI)
Perforin (MFI)
MM-FG
PBMC -T
BCAR -T
AlloNKT
Allo15BCAR -NKT
p = 0.0005
h
IFN-γ (pg/ml)
p = 0.0016

Supplementary Fig. 7. *In vitro* antitumor efficacy and mechanism of action (MOA) study of $^{Allo/15}$BCAR-NKT cells, related to Fig. 2.

a. Schematics showing the generation of indicated tumor cell lines, related to **Fig. 2g**.
b. Design of the Lenti/FlucGFP and Lenti/CD1d vectors.
c. FACS plots showing the expression of BCMA and CD1d on the indicated cells, related to **Fig. 2g**.
d. FACS plots showing the expression of NK-related molecules on ^{Allo}NKT cells. PBMC-derived NK (PBMC-NK) cells were included as a control.
e, **f.** Studying the NK killing mechanism, related to **Fig. 2i** and **2l**. **e.** Experimental design. A BCMA^{-} CD1d^{-} human K562 myelogenous leukemia cell line engineered with FG dual reporter was used (denoted as K562-FG). **f.** Tumor cell killing data at 12 h (^{Allo}NKT, n = 4; $^{Allo/15}$BCAR-NKT, n = 3).
g. FACS analyses of surface activation marker (i.e., CD69) and intracellular cytotoxic molecule (i.e., Perforin) in the indicated cells after coculturing with MM-FG cells for 24 hours (n = 3).
h. ELISA detection of IFN-γ secreted by the indicated cells after coculturing with MM-FG cells for 24 hours (n = 3).
Representative of 3 experiments. Data are presented as the mean ± SEM and were analyzed by 1-way ANOVA (**f, g, h**). ns, not significant.

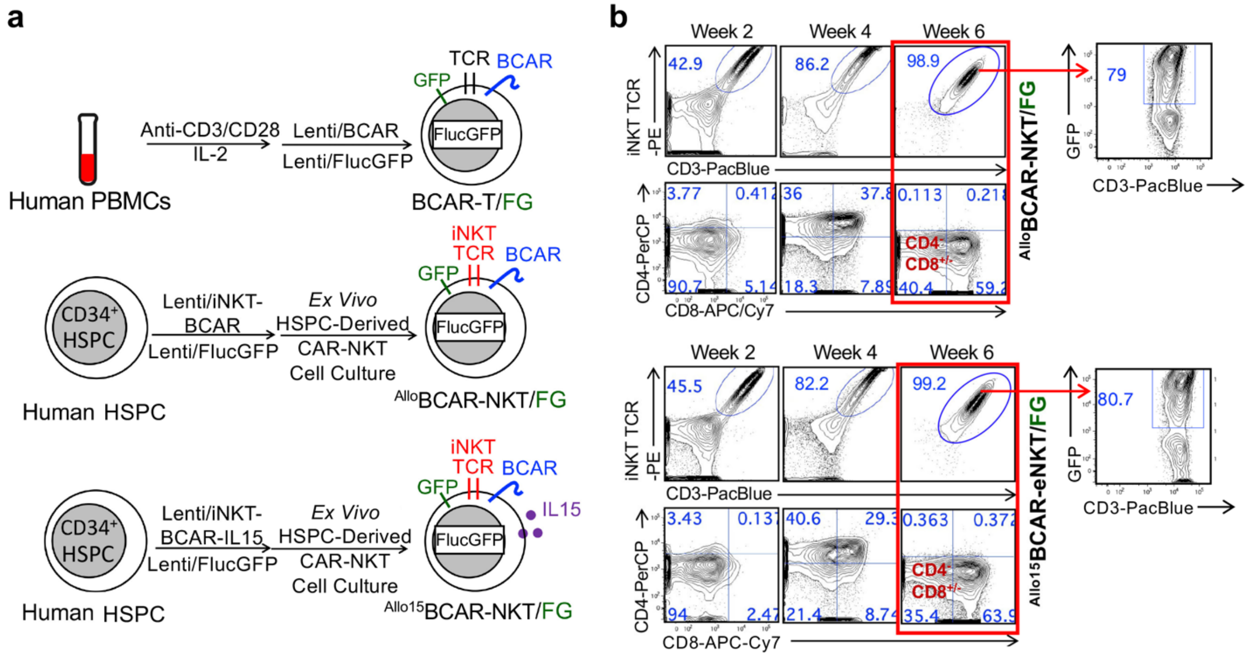

Supplementary Fig. 8. *In vivo* PK/PD study of $^{Allo/15}$BCAR-NKT/FG cells, related to Fig. 3.

a. Schematics showing the generation of indicated cells. FG, firefly luciferase and green fluorescence protein dual reporters.

b. FACS plots showing the development of ^{Allo}BCAR-NKT/FG and ^{Allo15}BCAR-NKT/FG cells. Representative of 3 experiments.

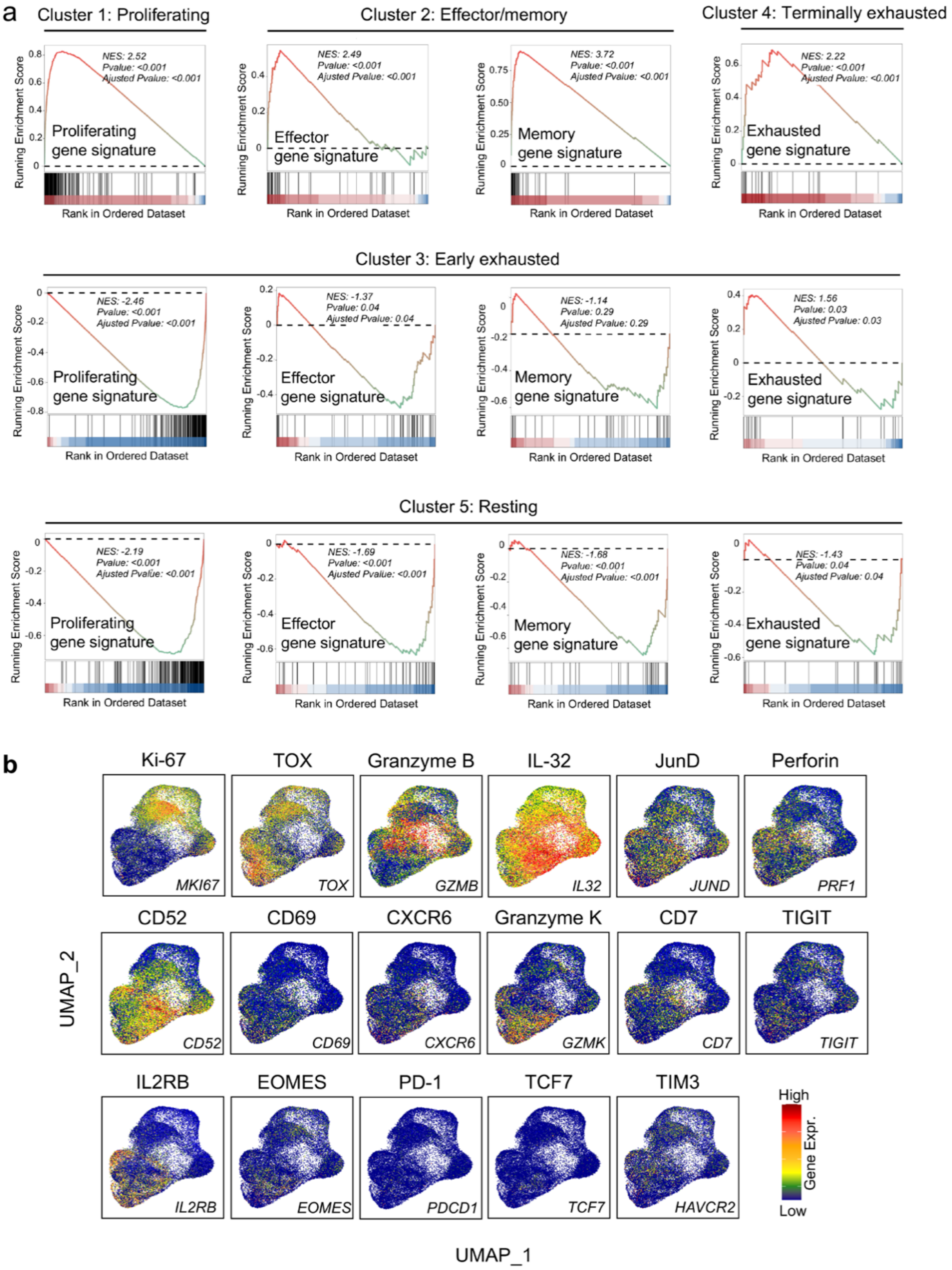

Supplementary Fig. 9. *In vivo* gene profiling of $^{Allo/15}$BCAR-NKT, related to Fig. 4.

a. Gene Set Enrichment Analysis (GSEA) plots showing the enrichment of gene signatures of proliferating, effector, memory, and exhausted cells in the indicated cell clusters. Nominal p value, calculated as two-tailed *t*-test, no adjustment since only one gene set was tested

b. UMAP plots showing the expression of the indicated signature genes.

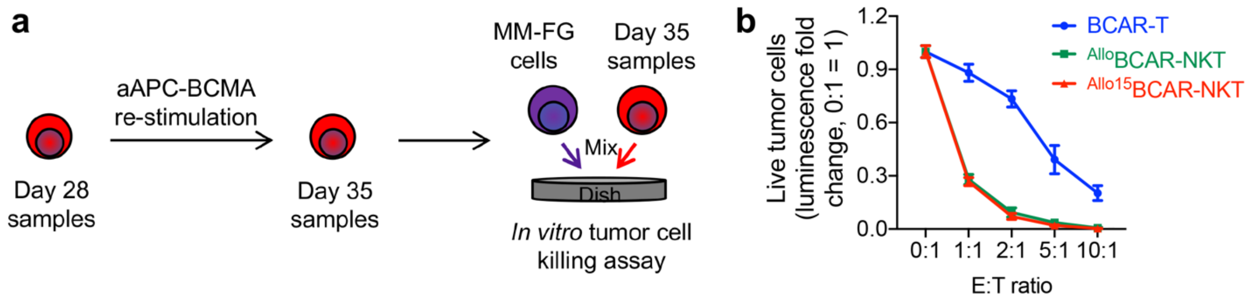

Supplementary Fig. 10. Antitumour capacity of Day 35 re-stimulation samples, related to Fig. 4.

a. Experimental design. Day 35 samples represented therapeutic cells derived from Day 28 samples and subsequently stimulated *in vitro* using BCMA-expressing aAPCs for one week. The Day 35 samples were then co-cultured with MM-FG tumor cells and tested for their antitumor capacity.

b. Tumor cell killing data at 24 h (n = 4, n indicates the Day 35 samples cultured from different batches).

Representative of 3 experiments.

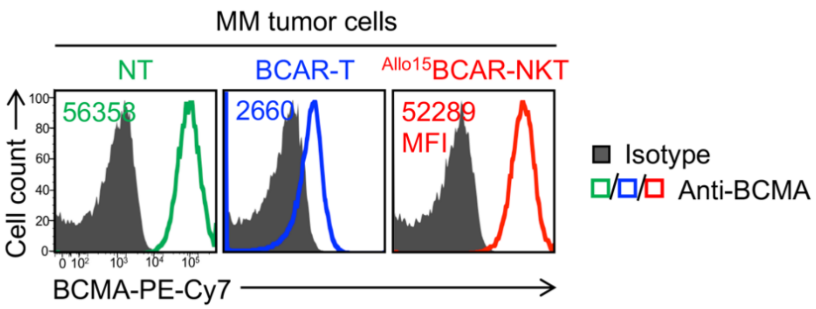

Supplementary Fig. 11. BCMA expression on MM-FG tumor cells in the indicated samples, related to Fig. 5a-5c. NT, sample collected from tumor-bearing NSG mice receiving no therapeutic cell treatment.

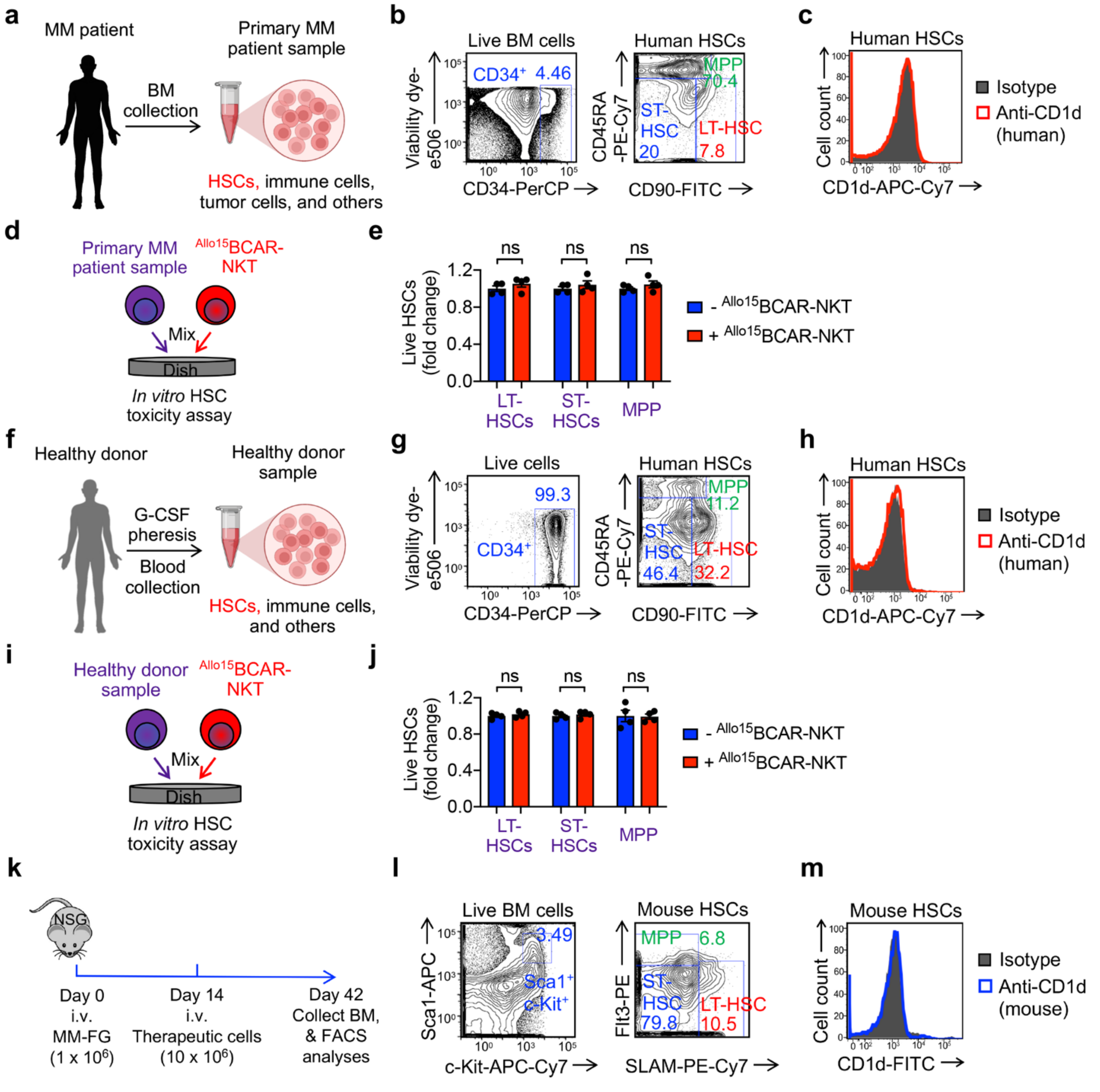

Supplementary Fig. 12. Studying the toxicity of ^{Allo15}BCAR-NKT cells against HSCs, related to Fig. 5.

a-e. Studying the toxicity of ^{Allo15}BCAR-NKT cells against human HSCs using primary MM patient BM samples. **a.** Diagram showing the BM collection from primary MM patients. **b**. FACS detection of long term HSCs (LT-HSCs; gated as CD34$^+$CD90$^+$CD45RA$^-$ cells) and short term HSCs (ST-HSCs; gated as CD34$^+$CD90$^-$CD45RA$^-$ cells) in the MM patient bone marrow (BM) cells. MPP, multi-potent progenitor cells (gated as CD34$^+$CD90$^-$CD45RA$^+$ cells). **c.** FACS plots showing the lack of CD1d expression on MM patient BM CD34$^+$ cells. **d**. Experimental design to study HSC killing by ^{Allo15}BCAR-NKT cells. MM patient BM cells were mixed with ^{Allo15}BCAR-NKT cells at 1:1 ratio and cultured *in vitro*. **e.** HSC killing data at 24 h (n = 4).

f-j. Studying the toxicity of ^{Allo15}BCAR-NKT cells against CD34$^+$ cells purified from G-CSF-mobilized healthy donor leukopak. **f.** Diagram showing the blood collection from G-CSF-mobilized

healthy donor leukopak. **g.** FACS detection of LT-HSCs and ST-HSCs in the G-CSF-mobilized healthy donor CD34$^+$ cells. **h.** FACS plots showing the lack of CD1d expression on G-CSF-mobilized healthy donor CD34$^+$ cells. **i**. Experimental design to study HSC killing by ^{Allo15}BCAR-NKT cells. G-CSF-mobilized healthy donor CD34$^+$ cells were mixed with ^{Allo15}BCAR-NKT cells at 1:1 ratio and cultured *in vitro*. **j.** HSC killing data at 24 h (n = 4).

k-m. Studying the toxicity of ^{Allo15}BCAR-NKT cells against mouse HSCs using a human MM xenograft NSG mouse model. **k**. Experimental design; related to **Fig. 5i**. On day 42 (28 days after ^{Allo15}BCAR-NKT cell injection), BM cells were collected from experimental mice and subjected to flow cytometry. **l.** FACS detection of mouse LT-HSCs (gated as Sca1$^+$c-Kit$^+$Flt3$^-$SLAM$^+$ cells) and ST-HSCs (gated as Sca1$^+$c-Kit$^+$Flt3$^-$SLAM$^-$ cells) in BM cells collected from the tumor-bearing experimental mice receiving ^{Allo15}BCAR-NKT cell treatment. **m.** FACS plots showing the lack of mouse CD1d expression on mouse HSCs.

Representative of 3 experiments. Data are presented as the mean ± SEM and were analyzed by two-tailed Student's *t* test (**e** and **j**). ns, not significant.

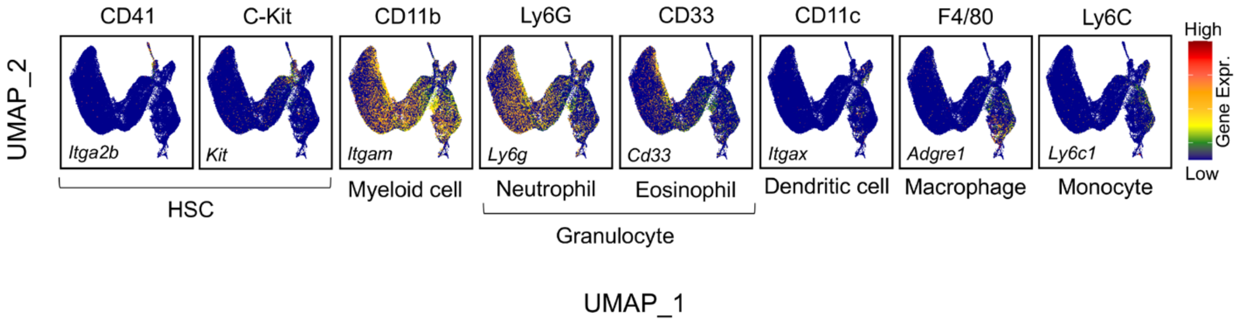

Supplementary Fig. 13. Cell cluster definition using lineage markers, related to Fig. 5j. UMAP plots are presented, showing the expression distribution of the indicated lineage markers.

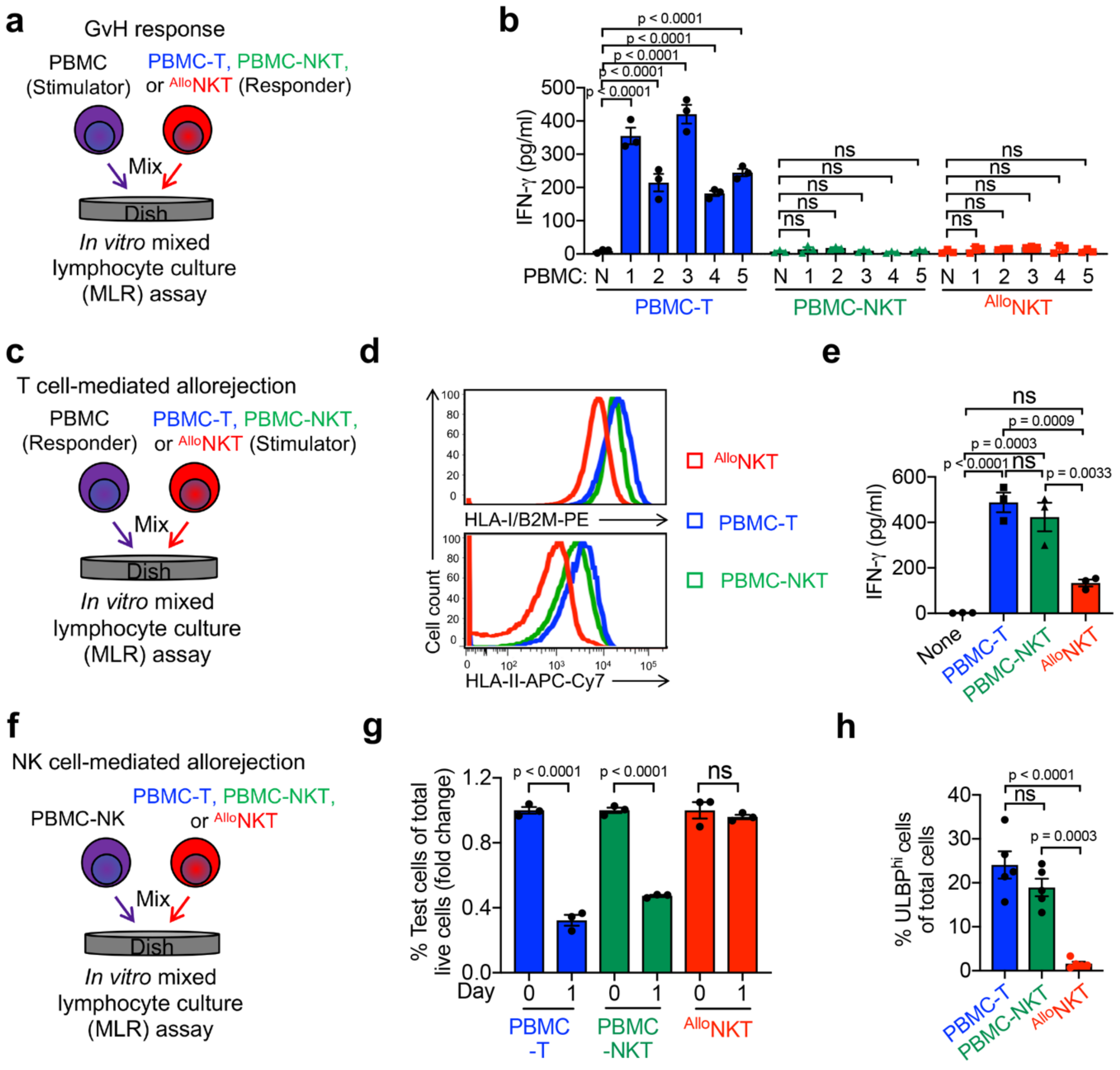

Supplementary Fig. 14. Studying the GvH response and allorejection of AlloNKT cells in comparison with PBMC-derived conventional αβ T and NKT cells, related to Fig. 6a-6h.

a, b. Studying the GvH response using an *in vitro* MLR assay. **a.** Experimental design. PBMCs from five different healthy donors were irradiated and used as stimulator cells. **b.** ELISA analyses of IFN-γ production on day 4 (n = 3). N, no addition of stimulator PBMCs.

c-e. Studying the T cell-mediated allorejection using an *in vitro* MLR assay. **c.** Experimental design. **d.** FACS measurement of cell surface HLA-I and HLA-II expression levels on the indicated cells. **e.** ELISA analyses of IFN-γ production on day 4 (n = 3).

f-h. Studying the NK cell-mediated allorejection using an *in vitro* MLR assay. **f.** Experimental design. **g.** FACS quantification of the indicated live cells on day 0 and day 1 (n = 3). **h.** FACS quantification of ULBP^{hi} cells on day 0 (n = 5, n indicates different donors).

Representative of 3 experiments. Data are presented as the mean ± SEM and were analyzed by two-tailed Student's *t* test (**g**), or 1-way ANOVA (**b, e, h**). ns, not significant.

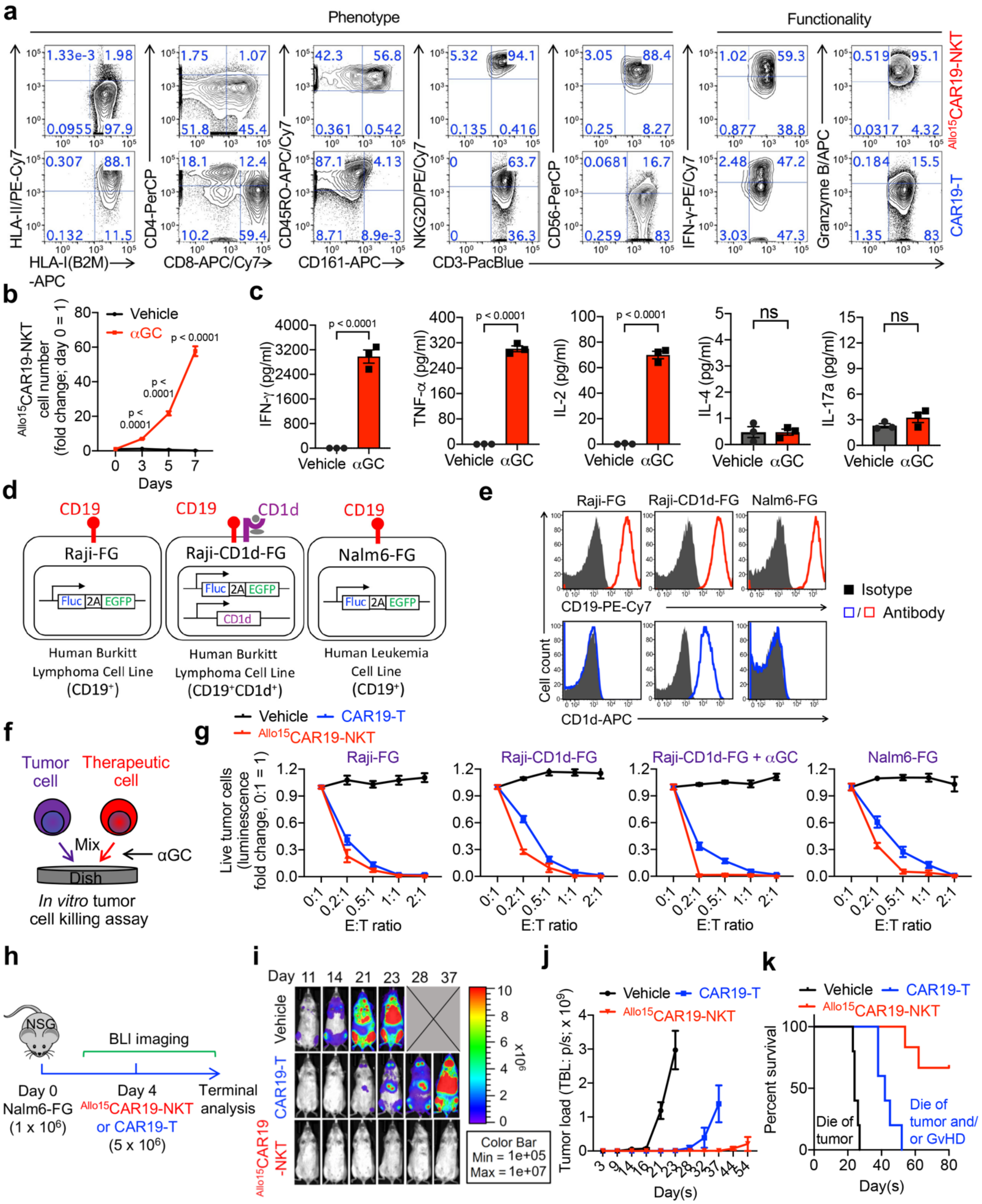

Supplementary Fig. 15. Characterization of ^{Allo15}CAR19-NKT cells.
a. FACS analyses of surface markers, and intracellular cytokines and cytotoxic molecules in ^{Allo15}CAR19-NKT cells. Healthy donor PBMC-derived conventional T cells engineered with the same CAR19 (denoted as CAR19-T cells) were included as a control.

b, c. Antigen response of ^{Allo15}CAR19-NKT cells. ^{Allo15}CAR19-NKT cells were stimulated with/out αGC/PBMC for one week (n = 3). **b.** Growth curve. **c.** ELISA analyses of effector cytokine production on day 7.
d. Schematics showing the indicated human tumor cell lines. Raji, human Burkitt's lymphoma cell line; Raji-FG, parental Raji cell line engineered to express the FG dual reporters; Raji-FG-CD1d, Raji-FG cell line further engineered to overexpress human CD1d; NALM-6, human acute lymphoblastic leukemia cell line; NALM-6-FG, parental NALM-6 cell line engineered to express the FG dual reporters.
e. FACS detection of CD19 and CD1d expression on the indicated tumor cell lines.
f, g. *In vitro* antitumor efficacy of ^{Allo}CAR19-NKT cells. **f.** Experiment design. **g.** Tumor cell killing data at 24 h (n = 4 from 4 different cell product donors).
h-k. Studying the *in vivo* antitumor efficacy of ^{Allo15}CAR19-NKT cells in a NALM-6-FG xenograft NSG mouse model. CAR19-T cells were included as a therapeutic cell control. **h.** Experimental design. **i.** BLI images showing tumor loads in experimental mice over time. **j.** Quantification of (**i**) (Vehicle and CAR19-T, n = 5; ^{Allo15}CAR19-NKT, n = 6). **k.** Kaplan-Meier survival curves of experimental mice over time (Vehicle and CAR19-T, n = 5; ^{Allo15}CAR19-NKT, n = 6).
Representative of 3 experiments. Data are presented as the mean ± SEM and were analyzed by two-tailed Student's *t* test (**b, c**). ns, not significant.

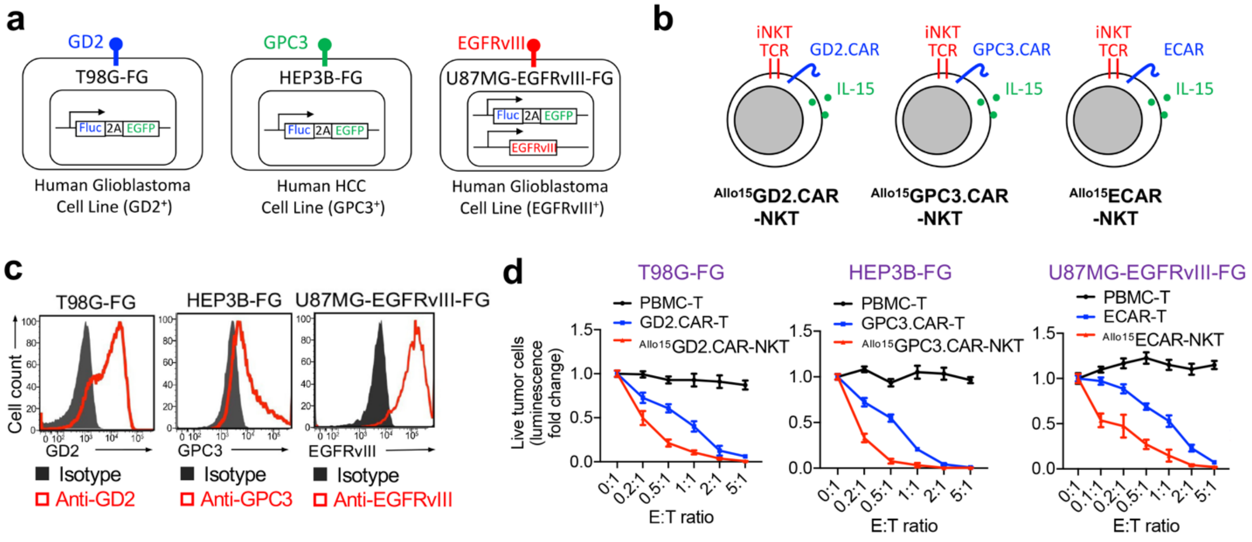

Supplementary Fig. 16. Solid tumor cell killing by ^{Allo15}CAR-NKT cells.

a. Schematics showing the tumor cell lines used in the study. T98G-FG, a T98G human glioblastoma cell line engineered to express FG; Hep3B-FG, a Hep3B human hepatocellular carcinoma cell line engineered with FG; U87MG-FG, a U87MG human glioblastoma cell line engineered with FG.

b. Schematics showing the ^{Allo15}CAR-NKT cells used in the study.

c. FACS measurements of antigen expression on the indicated solid tumor cell lines.

d. Tumor cell killing data at 24 h (n = 4). PBMC-T, and PBMC-derived conventional T cells engineered with the same GD2.CAR, GPC3.CAR, or ECAR (denoted as GD2.CAR-T, GPC3.CAR-T, or ECAR-T cells, respectively) were included as therapeutic cell controls.

Representative of 3 experiments.

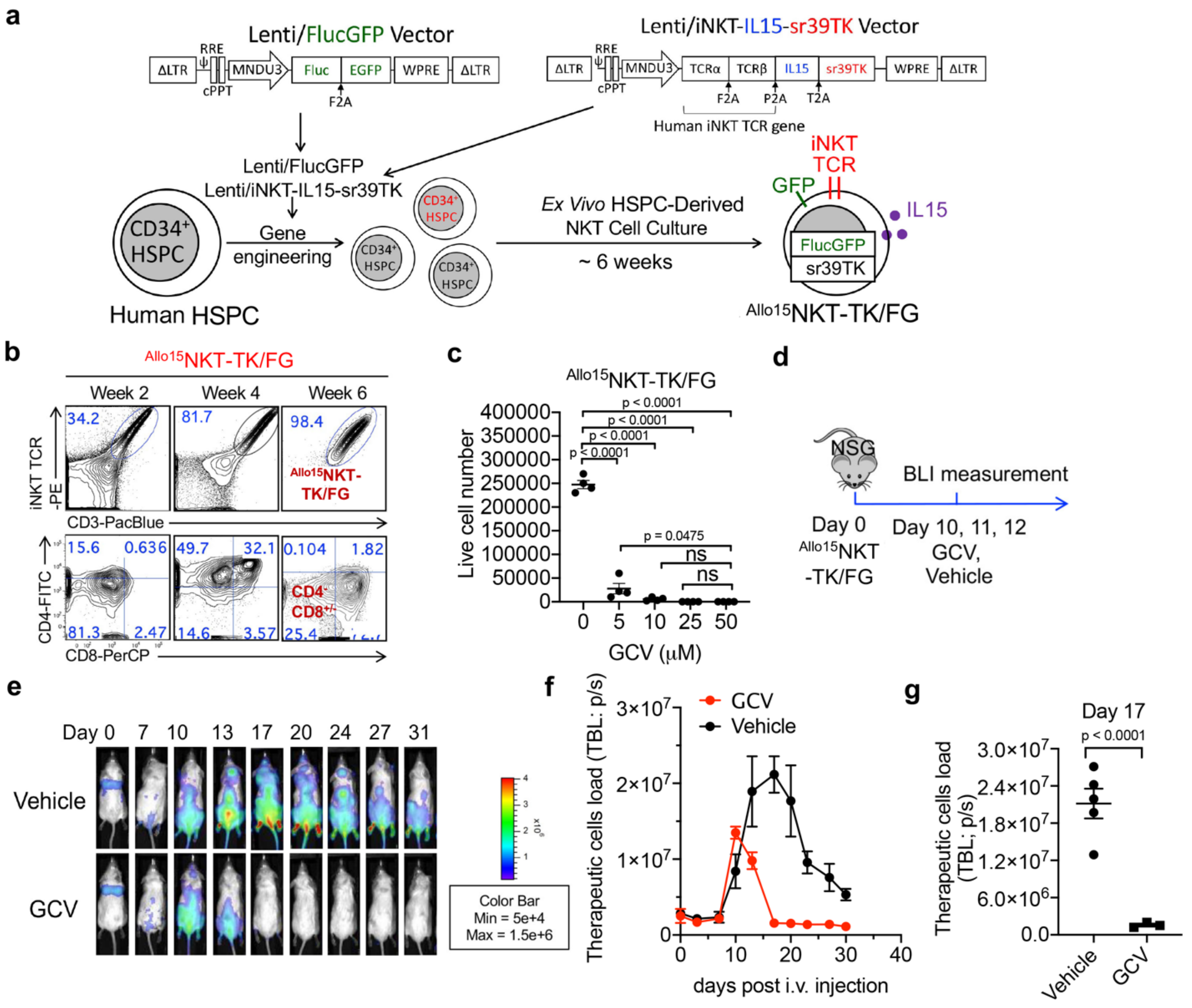

Supplementary Fig. 17. Incorporation of an sr39TK suicide switch in allogeneic NKT cells.
a. Schematic showing the experimental design to generate allogeneic HSPC-engineered NKT cells equipped with an IL-15 gene, an sr39TK suicide gene, and the FG dual-reporter genes (denoted as ^{Allo15}NKT-TK/FG cells). Lenti/iNKT-IL15-sr39TK, lentivector encoding iNKT TCR, IL-15, and sr39TK. Lenti/FlucGFP, lentivector encoding firefly luciferase and enhanced green fluorescence protein dual-reporters
b. FACS monitoring of ^{Allo15}NKT-TK/FG cell development.
c. *In vitro* depletion of ^{Allo15}NKT-TK/FG cells via GCV treatment. GCV, ganciclovir that specifically depleting cells expressing the sr39TK gene. ^{Allo15}NKT-TK/FG cells were cultured *in vitro* in the presence of gradient concentrations of GCV for 3 days, followed by quantification of live cells via FACS analysis (n = 4 from 4 different cell product donors).
d-g. *In vivo* depletion of ^{Allo15}NKT-TK/FG cells via GCV administration. **d.** Experimental design. **e.** BLI images showing the presence and dynamics of ^{Allo15}NKT-TK/FG cells in experimental animals over time. **f.** Quantification of (**e**). **g.** Quantification of ^{Allo15}NKT-TK/FG cells on day 17 (Vehicle, n = 5; GCV, n = 3).
Representative of 3 experiments. Data are presented as the mean ± SEM and were analyzed by 1-way ANOVA (**c**), or two-tailed Student's *t* test (**g**). ns, not significant.

CB donors	Gender	Race	ABO/Rh
1	Unknown	Black	A+
2	Male	Caucasian	O-
3	Male	Hispanic	O+
4	Male	Mixed	O+
5	Male	Caucasian	A+
6	Female	Caucasian	A+
7	Male	Caucasian	B+
8	Female	Caucasian	O-
9	Male	Caucasian	B-
10	Male	Mixed	O+
11	Female	Hispanic	O-
12	Male	Black	A-
13	Male	Asian	B+
14	Female	Unknown	O+
15	Female	Hispanic	A+

Supplementary Fig. 18. Cord blood donor information.

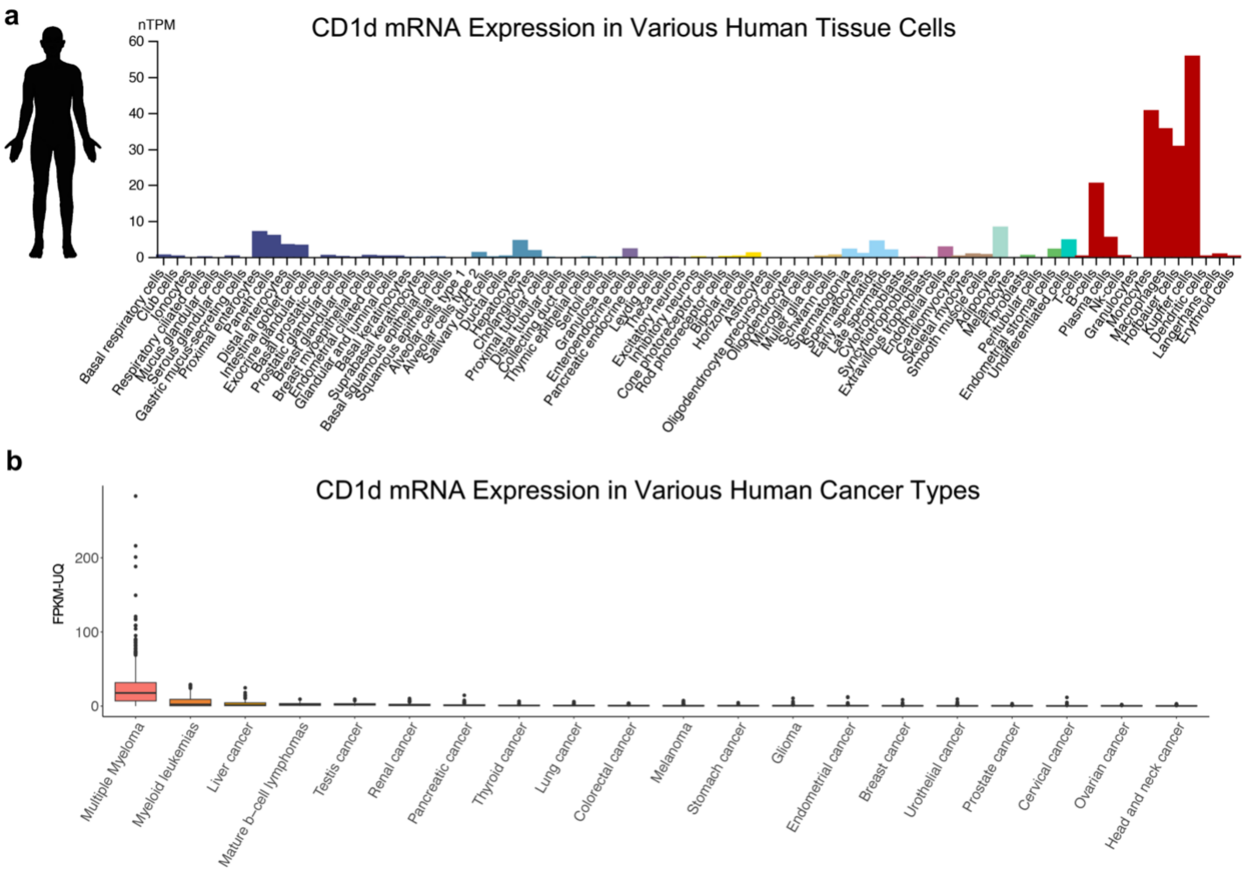

Supplementary Fig. 19. CD1d expression on various human tissue cells and cancer types.
a. CD1d mRNA expression in various human tissue cells. Data were adapted from The Human Protein Atlas (https://www.proteinatlas.org/ENSG00000158473-CD1D/summary/rna). nTPM, normalized transcripts per million.
b. CD1d mRNA expression in various human cancer types. Raw data were collected from The Cancer Genome Atlas (TCGA) and Multiple Myeloma Research Foundation (MMRF), and analyzed using the SummarizedExperiment and TCGAbiolinks packages in R. FPKM-UQ, fragments per kilobase of transcript per million mapped reads - upper quartile. Box and whisker plots encapsulating within exhibit the minimum, lower quartile, median, upper quartile, and maximum expression levels of each type of cells. n indicates the biologically independent sample sizes as follows: Multiple Myeloma: 859; Myeloid leukemias: 151; Liver cancer: 424; Mature b-cell lymphomas: 48; Testis cancer: 156; Renal cancer: 1028; Pancreatic cancer: 183; Thyroid cancer: 572; Lung cancer: 1153; Colorectal cancer: 701; Melanoma: 473; Stomach cancer: 448; Glioma: 175; Endometrial cancer: 589; Breast cancer: 1231; Urothelial cancer: 431; Prostate cancer: 554; Cervical cancer: 309; Ovarian cancer: 429; Head and neck cancer: 566.
