# Supplementary material for: Generation of allogeneic CAR-NKT cells from hematopoietic stem and progenitor cells using a clinically guided culture method
Source: Nat Biotechnol. 2024 May 14;43(3):329–44. doi: 10.1038/s41587-024-02226-y (PMC11919731; doi:10.1038/s41587-024-02226-y)
Supplement: Supplementary file 2 — Reporting Summary [file 41587_2024_2226_MOESM2_ESM.pdf]

nature portfolio

Corresponding author(s): Lili Yang

Last updated by author(s): Mar 7, 2024

Reporting Summary

Nature Portfolio wishes to improve the reproducibility of the work that we publish. This form provides structure for consistency and transparency in reporting. For further information on Nature Portfolio policies, see our Editorial Policies and the Editorial Policy Checklist.

Statistics

For all statistical analyses, confirm that the following items are present in the figure legend, table legend, main text, or Methods section.

n/a	Confirmed	
☐	☒	The exact sample size (*n*) for each experimental group/condition, given as a discrete number and unit of measurement
☐	☒	A statement on whether measurements were taken from distinct samples or whether the same sample was measured repeatedly
☐	☒	The statistical test(s) used AND whether they are one- or two-sided<br>*Only common tests should be described solely by name; describe more complex techniques in the Methods section.*
☐	☒	A description of all covariates tested
☐	☒	A description of any assumptions or corrections, such as tests of normality and adjustment for multiple comparisons
☐	☒	A full description of the statistical parameters including central tendency (e.g. means) or other basic estimates (e.g. regression coefficient) AND variation (e.g. standard deviation) or associated estimates of uncertainty (e.g. confidence intervals)
☐	☒	For null hypothesis testing, the test statistic (e.g. *F*, *t*, *r*) with confidence intervals, effect sizes, degrees of freedom and *P* value noted<br>*Give P values as exact values whenever suitable.*
☒	☐	For Bayesian analysis, information on the choice of priors and Markov chain Monte Carlo settings
☒	☐	For hierarchical and complex designs, identification of the appropriate level for tests and full reporting of outcomes
☒	☐	Estimates of effect sizes (e.g. Cohen's *d*, Pearson's *r*), indicating how they were calculated

Our web collection on statistics for biologists contains articles on many of the points above.

Software and code

Policy information about availability of computer code

Data collection	MACSQuant Analyzer 10 Flow Cytometer, Illumina NovaSeq 6000 sequencer, Infinite M1000 microplate reader (Tecan), Spectral Advanced Molecular Imaging (AMI) HTX imaging system (Spectral instrument Imaging), ChemiDoc™ Imaging Systems (BIO-RAD)
Data analysis	FlowJo software (version 9), AURA imaging software (version 3.2.0), Graphpad Prism (version 8), Image J (version 1.53s), R package Seurat (v.4.0.0)

For manuscripts utilizing custom algorithms or software that are central to the research but not yet described in published literature, software must be made available to editors and reviewers. We strongly encourage code deposition in a community repository (e.g. GitHub). See the Nature Portfolio guidelines for submitting code & software for further information.

Data

Policy information about availability of data

All manuscripts must include a data availability statement. This statement should provide the following information, where applicable:

- Accession codes, unique identifiers, or web links for publicly available datasets
- A description of any restrictions on data availability
- For clinical datasets or third party data, please ensure that the statement adheres to our policy

All data associated with this study are present in the paper or Supplemental Information. The genomics data generated during this study are available from the public repository Gene Expression Omnibus Database: GSE (scTCR-seq, related to Fig. 1h), GSE245375 (Bulk RNA-seq, related to Fig. 1i), GSE241996 (scRNA-seq for

the in vitro study related to Fig. 1g), GSE241998 (scRNA-seq for the in vivo study related to Fig. 4), GSE241999 (scRNA-seq for the in vivo study related to Fig. 5a-5c), GSE241997 (scRNA-seq for the in vivo study related to Fig. 5h-5n), GSE239648 (Methy-seq, related to Fig. 6n). Source data are provided with this paper. Additional information and materials will be made available upon reasonable request.

Human research participants

Policy information about studies involving human research participants and Sex and Gender in Research.

Reporting on sex and gender	Sex and gender are not considered in the design of this study.
Population characteristics	Patient #1<br>Collection data: 2/13/2023<br>Age: 64<br>Diagnosis: Multiple meyloma<br>FISH: gain of 1q, gain of 5p, monosomy 13, and loss of 17p (high-risk cytogenetics)<br>Number of prior lines of therapy: 6<br><br>Patient #2<br>Collection data: 3/06/2023<br>Age: 65<br>Diagnosis: Multiple meyloma<br>FISH: gain of 1q and CCND1-IGH fusions [t(11;14)].<br>Number of prior lines of therapy: 4<br><br>Patient #3<br>Collection data: 3/29/2023<br>Age: 61<br>Diagnosis: Multiple meyloma<br>FISH: +5, +11q, and 16q-.<br>Number of prior lines of therapy: 5
Recruitment	Healthy donor PBMCs were obtained from the UCLA/CFAR Virology Core Laboratory. Primary MM patient bone marrow samples were collected at the Ronald Reagan UCLA Medical Center from consented patients and processed.
Ethics oversight	Healthy donor PBMCs were obtained from the UCLA/CFAR Virology Core Laboratory without identification information under federal and state regulations. Primary MM patient bone marrow samples were collected at the Ronald Reagan UCLA Medical Center from consented patients through an IRB-approved protocol (IRB#21-001444).

Note that full information on the approval of the study protocol must also be provided in the manuscript.

Field-specific reporting

Please select the one below that is the best fit for your research. If you are not sure, read the appropriate sections before making your selection.

☒ Life sciences ☐ Behavioural & social sciences ☐ Ecological, evolutionary & environmental sciences

For a reference copy of the document with all sections, see nature.com/documents/nr-reporting-summary-flat.pdf

Life sciences study design

All studies must disclose on these points even when the disclosure is negative.

Sample size	For all studies samples size were defined on the basis of previous studies in the laboratory (PMID: 34841295, 37938576, and 34112755). For in vivo studies, between 3 to 10 mice per group were used dependent on the experimental design and were able to reach a significant level of 0.05. For in vitro studies, at least 3 biological group were used as cell donors with technical replicates. For studies involving primary MM patient samples, at least 3 patient samples per group were used.
Data exclusions	No data were excluded from the analyses.
Replication	All experiments were performed independently. Replicates of each individual experiment was stated in its figure legends. The experimental findings were reproduced with similar results. For single cell RNA sequencing, a mixture of 10 experimental mice were combined for each group.
Randomization	For in vivo studies, all mice received tumor cells at Day 0 then were randomized to split into different groups prior to therapeutic cells injection. All the in vitro and in vivo experiments each group was selected randomly.
Blinding	Experiments were not performed in a blinded fashion. All data were analyzed by software with objective standard.

Reporting for specific materials, systems and methods

We require information from authors about some types of materials, experimental systems and methods used in many studies. Here, indicate whether each material, system or method listed is relevant to your study. If you are not sure if a list item applies to your research, read the appropriate section before selecting a response.

Materials & experimental systems

n/a	Involved in the study
☐	☒ Antibodies
☐	☒ Eukaryotic cell lines
☒	☐ Palaeontology and archaeology
☐	☒ Animals and other organisms
☒	☐ Clinical data
☒	☐ Dual use research of concern

Methods

n/a	Involved in the study
☒	☐ ChIP-seq
☐	☒ Flow cytometry
☒	☐ MRI-based neuroimaging

Antibodies

Antibodies used

Fluorochrome-conjugated antibodies specific for human CD45 (Clone H130, CAT#304026, 1:500 dilution), TCRαβ (Clone I26, CAT#306716, 1:25 dilution), CD3 (Clone HIT3a, CAT#300329, 1:500 dilution), CD4 (Clone OKT4, CAT#317414, 1:400 dilution), CD8 (Clone SK1, CAT#344714, 1:500 dilution), CD45RO (Clone UCHL1, CAT#304216, 1:200 dilution), CD45RA (Clone HI100, CAT#304105, 1:5000 dilution), CD161 (Clone HP-3G10, CAT#339928, 1:50 dilution), CD69 (Clone FN50, CAT#310909, 1:50 dilution), CD56 (Clone HCD56, CAT#362545, 1:10 dilution), CD1d (Clone 51.1, CAT#350308, 1:50 dilution), BCMA (19F2, CAT#357503, 1:50 dilution), CD14 (Clone HCD14, CAT#325608, 1:100 dilution), CD19 (Clone HIB19, CAT#363005, 1:100 dilution), CD11b (Clone ICRF44, CAT#301330, 1:500 dilution), CD15 (W6D3, CAT#323021, 1:500 dilution), CD112 (Clone TX31, CAT#337409, 1:200 dilution), CD155 (Clone SKII.4, CAT#337613, 1:200 dilution), MICA/MICB (Clone 6D4, CAT#320908, 1:50 dilution), Ganglioside GD2 (14G2a, CAT#357323, 1:50 dilution), NKG2D (Clone 1D11, CAT#320812, 1:50 dilution), DNAM-1 (Clone 11A8, CAT#338312, 1:50 dilution), CD158 (KIR2DL1/S1/S3/S5) (Clone HP-MA4, CAT#339510, 1:50 dilution), NKp30 (Clone P30-15, CAT#325207, 1:50 dilution), NKp44 (Clone P44-8, CAT#325107, 1:50 dilution), CD16 (Clone 3G8, CAT#302011, 1:50 dilution), NKG2A (Clone S19004C, CAT#375103, 1:50 dilution), NKG2C (Clone S19005E, CAT#375003, 1:50 dilution), CD62L (Clone P44-8, CAT#304813, 1:50 dilution), CD134 (Clone Ber-ACT35, CAT#350008, 1:50 dilution), LEF1 (Clone W17021C, CAT#621051, 1:50 dilution), CD8A (Clone C8/144B, CAT#372092, 1:500 dilution), CD8B (Clone QA20A40, CAT#387305, 1:500 dilution), IFN-γ (Clone B27, CAT#506518, 1:50 dilution), Granzyme B (Clone QA16A02, CAT#372204, 1:4000 dilution), Perforin (Clone dG9, CAT#308126, 1:50 dilution), TNF-α (Clone Mab11, CAT#502912, 1:4000 dilution), IL-2 (Clone MQ1-17H12, CAT#500341, 1:200 dilution), β2-microglobulin (B2M) (Clone 2M2, CAT#316312, 1:5000 dilution), HLA-DR (Clone L243, CAT#307618, 1:250 dilution), and HLA-DR, DP, DQ (Clone Tü 39, CAT#361707, 1:250 dilution) were purchased from BioLegend. Fluorochrome-conjugated antibodies specific for mouse CD45 (Clone S18009F, CAT#157607, 1:5000 dilution), GR1 (Clone RB6-8C5, CAT#108411, 1:1000 dilution), CD1d (Clone 1B1, CAT#123521, 1:100 dilution), CD11b (Clone M1/70, CAT#101205, 1:5000 dilution), Sca1 (Clone D7, CAT#108111, 1:100 dilution), Flt3 (Clone A2F10, CAT#135305, 1:100 dilution), SLAM (Clone TC15-12F12.2, CAT#115913, 1:50 dilution), c-Kit (Clone 2B8, CAT#105825, 1:50 dilution) were purchased from BioLegend. Fluorochrome-conjugated antibody specific for human Glypican 3 (GPC3; Clone 024, CAT#ab275695, 1:200 dilution) was purchased from Abcam. Fluorochrome-conjugated antibodies specific for human CD34 (Clone 581, CAT#555822, 1:100 dilution) and human iNKT TCR Vα24-Jβ18 (Clone 6B11, CAT#552825, 1:10 dilution) were purchased from BD Biosciences. Fluorochrome-conjugated antibody specific for human iNKT TCR Vβ11 (Clone C21, CAT#A66905, 1:50 dilution) was purchased from Beckman-Coulter. Fluorochrome-conjugated antibodies specific for human ULBP-1 (Clone 170818, CAT#FAB1380P, 1:50 dilution) and ULBP-2,5,6 (Clone 165903, CAT#FAB1298A5, 1:50 dilution) were purchased from R&D Systems. A goat anti-mouse IgG F(ab')2 secondary antibody (CAT#31803, 1:50 dilution) was purchased from ThermoFisher. Fixable Viability Dye eFluor506 (e506, CAT#65-0866-14, 1:500 dilution) was purchased from Affymetrix eBioscience; mouse Fc Block (anti-mouse CD16/32, CAT#553142, 1:50 dilution) was purchased from BD Biosciences; and human Fc Receptor Blocking Solution (TrueStain FcX, CAT#422302, 1:100 dilution) was purchased from BioLegend.

The following antibodies were used to blot for the proteins of interest: anti-human CIITA (Invitrogen, CAT#PA5-21031, 1:1000 dilution), anti-human p-STAT1(Y701) (Clone 58D6, Cell Signaling Technology, CST, CAT#9167, 1:1000 dilution), anti-human STAT1 (Clone D1K9Y, CST, CAT#14994, 1:1000 dilution), anti-human IRF-1 (Clone D5E4, CST, CAT#8478, 1:1000 dilution), anti-human SP1 (Clone D4C3, CST, CAT#9389, 1:1000 dilution), and secondary anti-rabbit IgG (CST, CAT#7074, 1:1000 dilution). Beta-Actin (Clone D6A8, CST, CAT#8457, 1:3000 dilution) and GAPDH (Clone D16H11, CST, CAT#5174, 1:3000 dilution) were used as internal controls.

Mouse IL-6 was quantified by ELISA with paired purified anti-mouse IL-6 antibody (CAT#504501, 1:250 dilution) and biotin anti-mouse IL-6 antibody (CAT#504601, 1:500 dilution). These antibodies were purchased from BioLgend.

Validation

All the antibodies used are from commercial sources and have been validated by the vendors. Validation data are available on the manufacturer's website. Each antibody purchased for this study has relevant citation and profile.

Eukaryotic cell lines

Policy information about cell lines and Sex and Gender in Research

Cell line source(s)

Human multiple myeloma cell line MM.1S (ATCC #CRL-2974), chronic myelogenous leukemia cell line K562 (ATCC #CCL-243), Burkitt's lymphoma cell line Raji (ATCC #CCL-86), acute lymphoblastic leukemia cell line NALM-6 (ATCC #CRL-3273), melanoma cell line A375 (ATCC #CRL-1619), glioblastoma cell line T98G (ATCC #CRL-1690) and U87MG (ATCC #HTB-14),

	hepatocellular carcinoma cell line HEP3B (ATCC #HB-8064), and HEK 293T (ATCC #CRL-3216) were purchased from the American Type Culture Collection (ATCC). All cell lines used in this study were tested mycoplasma free.
Authentication	MM.1S, K562, Raji, NAML-6, A375, T98G, U87MG, HEP3B, HEK 293T cells were authenticated by ATCC. All the FG engineered cells and BCMA knockout cells were not authenticated.
Mycoplasma contamination	All cell lines were tested negative for mycoplasma contamination.
Commonly misidentified lines (See ICLAC register)	No misidentified lines were used.

Animals and other research organisms

Policy information about studies involving animals; ARRIVE guidelines recommended for reporting animal research, and Sex and Gender in Research

Laboratory animals	NOD.Cg-PrkdcSCIDIl2rgtm1Wjl/SzJ (NOD/SCID/IL-2Rγ-/-, NSG) mice were maintained in the animal facilities of the University of California, Los Angeles (UCLA) under the following housing conditions: temperature ranging from 68°F to 79°F, humidity maintained at 30% to 70%, a light cycle of On at 6:00 am and Off at 6:00 pm, and room pressure set to negative. 6-10 weeks old female mice were used for all experiments unless otherwise indicated.
Wild animals	No wild animals were used.
Reporting on sex	6-10 weeks old female mice were used for all experiments unless otherwise indicated.
Field-collected samples	This study did not involve field-collected samples.
Ethics oversight	All animals were maintained at the UCLA animal facilities and all animal experiments were approved by the Institutional Animal Care and Use Committee of UCLA.

Note that full information on the approval of the study protocol must also be provided in the manuscript.

Flow Cytometry

Plots

Confirm that:

- [x] The axis labels state the marker and fluorochrome used (e.g. CD4-FITC).
- [x] The axis scales are clearly visible. Include numbers along axes only for bottom left plot of group (a 'group' is an analysis of identical markers).
- [x] All plots are contour plots with outliers or pseudocolor plots.
- [x] A numerical value for number of cells or percentage (with statistics) is provided.

Methodology

Sample preparation	Purified cord blood derived human CD34+ cells were purchased from the HemaCare. Healthy donor PBMCs were obtained from the UCLA/CFAR Virology Core Laboratory without identification information under federal and state regulations. Primary MM patient bone marrow samples were collected at the Ronald Reagan UCLA Medical Center from consented patients through an IRB-approved protocol (IRB#21-001444) and processed. AlloNKT and Allo/15CAR-NKT cells were generated by differentiating gene-engineered cord blood CD34+ HSCs in a 5-stage Ex Vivo HSC-Derived NKT Cell Culture. Healthy donor PBMCs were used to generate the PBMC-derived conventional αβ T, NKT, and NK cells (denoted as PBMC-Tc, PBMC-NKT, and PBMC-NK cells, respectively). In mouse study, cells were collected from mouse organs (e.g., bone marrow, spleen, liver, lung, blood, and peritoneal cavity),
Instrument	MACSQuant Analyzer 10 flow cytometer (Miltenyi Biotech)
Software	FlowJo software version 9 (BD Biosciences)
Cell population abundance	For cell sorting, highest purity method was used in FACSAria II Flow Cytometer and ?98% post-sorting purity was verified through flow cytometry analysis.
Gating strategy	SSC-A/FSC-A, SSC-W/SSC-H and FSC-W/FSC-H were used to identify singlets. Fixable Viability Dye eFluor 506 (Biolegend) was used to exclude dead cells in flow analysis. DAPI (Thermo Fisher Scientific) was included to exclude dead cells in FACS sorting. Gating strategy for each cell subtype in each experiment was described in the article and provided as panels in supplementary figures.

- [x] Tick this box to confirm that a figure exemplifying the gating strategy is provided in the Supplementary Information.
